# Supplementary material for: A DBHS family member regulates male determination in the filariasis vector Armigeres subalbatus
Source: Nat Commun. 2023 Apr 21;14:2292. doi: 10.1038/s41467-023-37983-y (PMC10121658; doi:10.1038/s41467-023-37983-y)
Supplement: Supplementary file 1 — Supplementary Information [file 41467_2023_37983_MOESM1_ESM.pdf]

SUPPLEMENTARY INFORMATION

A novel DBHS gene family member regulates male determination in the filariasis vector, *Armigeres subalbatus*

Peiwen Liu[1]*, Wenqiang Yang[1]*, Ling Kong[1]*, Siyu Zhao[1], Zhensheng Xie[1], Yijie Zhao[1], Yang Wu[1], Yijia Guo[1], Yugu Xie[1], Tong Liu[1], Binbin Jin[1], Jinbao Gu[1], Zhijian Jake Tu[2]†, Anthony A. James[3]†, Xiao-Guang Chen[1]†

Affiliations:

[1] Department of Pathogen Biology, Institute of Tropical Medicine, School of Public Health, Southern Medical University, Guangzhou, Guangdong 510515, China.

[2] Department of Biochemistry and the Fralin Life Sciences Institute, Virginia Tech, Blacksburg, VA 24061, US.

[3]Department of Microbiology & Molecular Genetics, University of California, Irvine, CA 92697, US.

*These authors contributed equally to this work.

†Corresponding authors: xgchen@smu.edu.cn, jaketu@vt.edu, aajames@uci.edu.

This document contains:

Supplementary Figures 1 to 10
Supplementary Tables 1 to 5
Supplementary Text 1 to 3

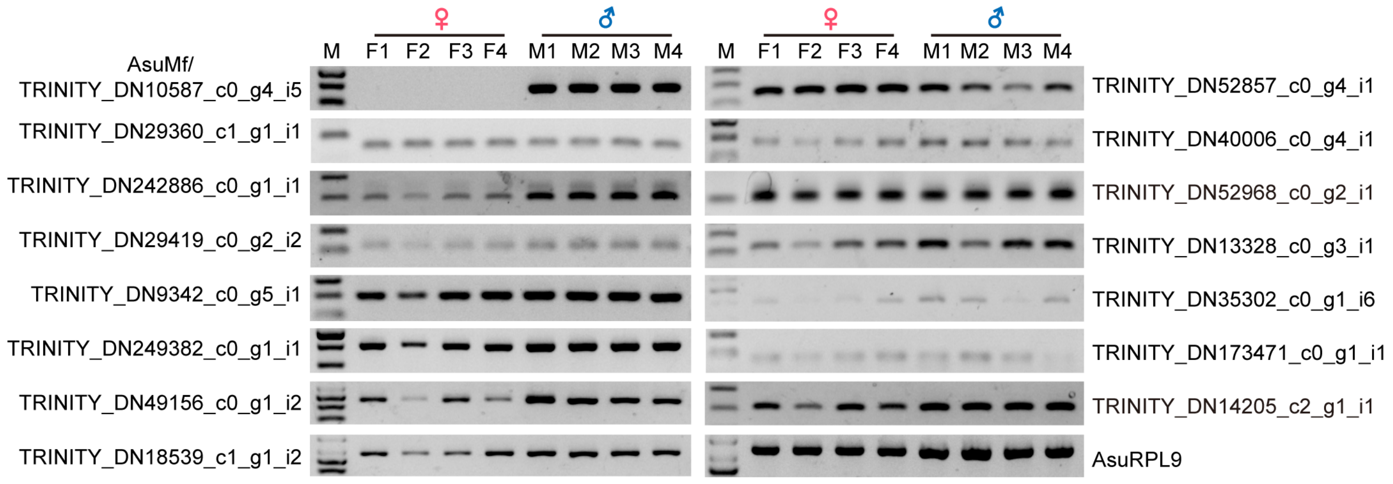

Supplementary Figure 1. Genomic DNA PCR to validate male-specific candidate genes in *Ar. subalbatus*. For each sample, genomic DNA was extracted from a pool of five male (♂) or female (♀) mosquitoes, respectively, and used for PCR amplification. Primers for the 14 candidate genes (representing 15 candidate transcripts) amplified a PCR product in at least one female sample. Primers for the positive control *AsuRPL9* amplified a PCR product in female and male DNA. Primers for *AsuMf* amplified a PCR product limited to male DNA (Fig. 1b). Similar results were obtained in three independent experiments. Source data are provided as a Source Data file.

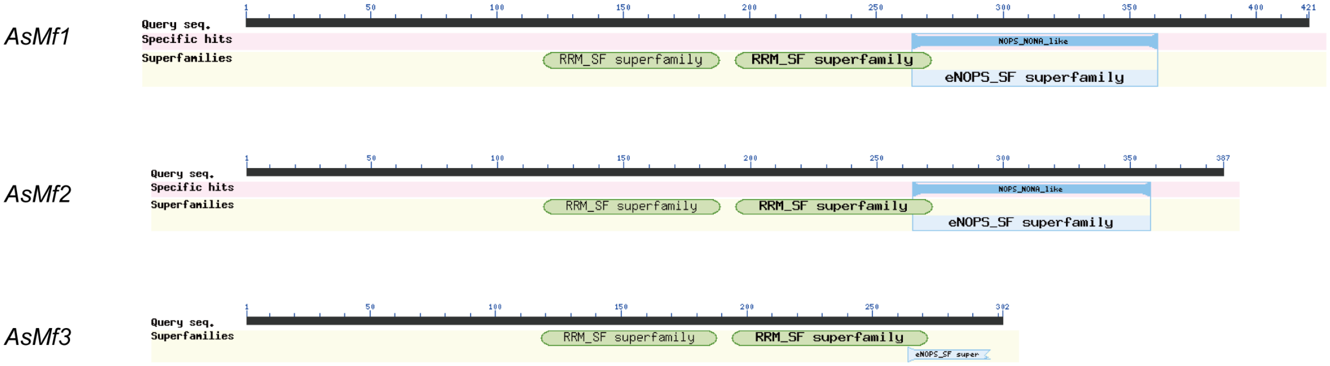

Supplementary Figure 2. The *AsuMf* polypeptide contains two RNA recognition motifs (RRM) and a NOPS domain. This figure is generated using the NCBI conserved domain search. Three major hits show low e-value (< 1e-10) in *AsuMf1-3* (Table S3). *AsuMf3* contains a partial NOPS domain.

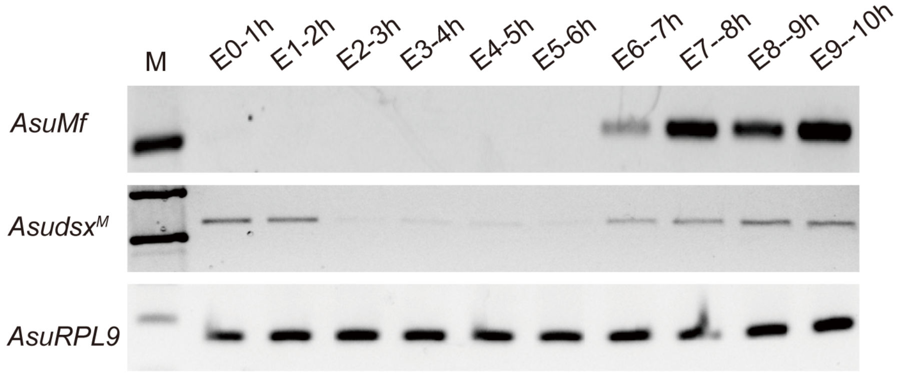

Supplementary Figure 3. Reverse transcription PCR expression profiles of *AsuMf* from 0–10 hour embryo cDNA samples starting at 0 to 1 hour (E0-1h) in 1-hour increments. Approximately 200 embryos were the source of RNA for RT-PCR. *AsuMf* transcripts are first evident in E6-7 h embryos, and throughout all subsequent developmental stages in males (see Fig. 1b). The length of *AsuMf* PCR product is 286 bp. Maternally deposited $Asudsx^{M}$ disappears by approximately 2-3 h, and the zygotic $Asudsx^{M}$ reappears at 6–7 h, following *AsuMf* expression. The length of $Asudsx^{M}$ amplicon is 315 bp. The positive control AsuRPL9 amplifies a 164 bp PCR product. Similar results were obtained in three independent experiments. Source data are provided as a Source Data file.

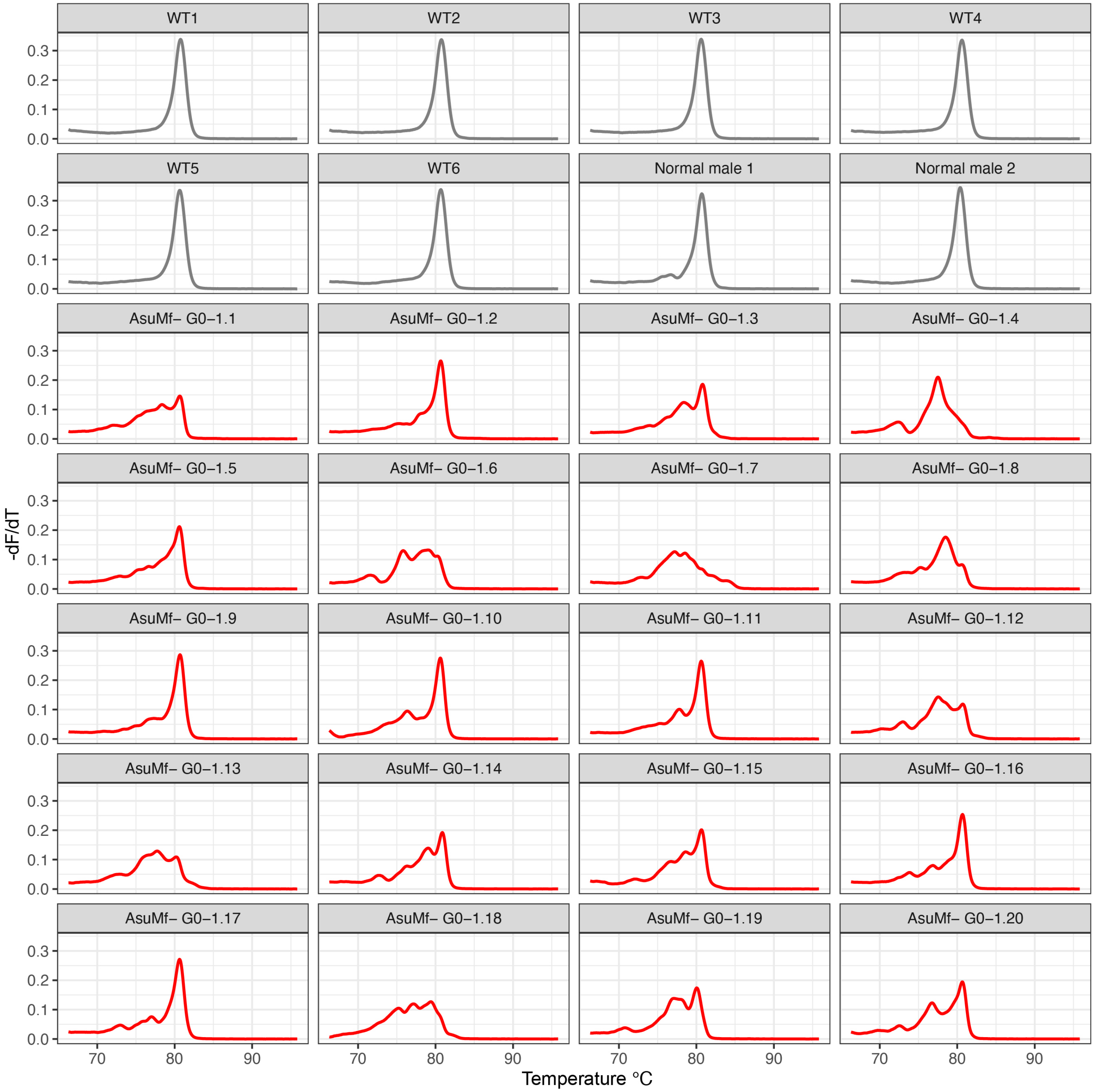

Supplementary Figure 4. AsuMf-sgRNA guided CRISPR/Cas9 induces mutations at the cleavage site. High resolution melt analysis (HRMA) was performed on 22 males from the Cas9 protein and AsuMf-sgRNA co-injection experiment (Supplementary Table 7) to detect mutations. While two of the 22 individuals with normal male phenotypes show similar melt curves (grey, Normal male 1 and 2) compared to the six wild-type males (grey, WT1-6), the 20 partially-feminized or malformed males show different melt curves (red) compared to the WT males. In all cases, genomic DNA was extracted from whole bodies.

	AsMf-sgRNA PAM	
AsuMf WT	AATGAAAAATTCCTCCCCGGGACCACTGCGGCGGCGCT	
AsuMf-G0-1.2-2	AATGAAAAATTCCTCCCCGGGACC**TC**CTGCGGCGGCGCT	-1,+2
AsuMf-G0-1.2-3	AATGAAAAATTCCTCCCCGGGAC---TGCGGCGGCGCT	-3
AsuMf-G0-1.2-4	AATGAAAAATTCCTCCCCGGGAC---TGCGGCGGCGCT	-3
AsuMf-G0-1.3-4	AATGAAAAATTCCTCC-----------CCGGCGGCGCT	-11
AsuMf-G0-1.3-5	AATGAAAAATTCCTCCCC--------TGCGGCGGCGCT	-8
AsuMf-G0-1.3-8	AATGAAAAATTCCTCCC--------------CGGCGCT	-14
AsuMf-G0-1.4-2	AATGAAAAATTCTTCCCCGGGAC----GCGGCGGCGCT	-4
AsuMf-G0-1.4-3	AATGAAAAATTCCTCCCCGGGACCA**GCG**GCGGCGGCGCT	-2,+3
AsuMf-G0-1.4-4	AATGAAAAATTCCTCCCC--------TGCGGCGGCGCT	-8
AsuMf-G0-1.4-10	AATGAAAAATTCCTCCCCGGGTCC--TGCGGCGGCGCT	-2
AsuMf-G0-1.6-3	AATGAAAAATTCCTCCCCGGGACC**GCT**--------GCT	-11,+3
AsuMf-G0-1.6-6	AATGAAAAATTCCTCCCCGGGAC---------GGCGCT	-9
AsuMf-G0-1.6-7	AATGAAAAATTCCTCCCCGGGACCA**TTAACGG**GGCGGCGCT	-4,+7
AsuMf-G0-1.6-9	AATGAAAAATTCCTCCCCGGGACC--TGCGGCGGCGCT	-2
AsuMf-G0-1.6-11	AATGAAAAA----------------CTGCGGCGGCGCT	-16
AsuMf-G0-1.7-2	AATGAAAAATTCCTCCCCGGGACCA**TACGTAGCTTTTTGTT**CTGCGGCGCGCGT	+16
AsuMf-G0-1.7-3	AATGAAAAATTCCTCCCCGGGAC---**G**GCGGCGGCGCT	-4,+1
AsuMf-G0-1.7-6	AATGAAAAATTCCTCCCC--------TGCGGCGGCGCT	-8
AsuMf-G0-1.7-7	AATGAAAAATTCCTCCCCGGGACC**CAAACAC**CTGCGGCGGCGCT	-1,+7
AsuMf-G0-1.7-12	AATGAAAAATTCCTCCCCGGGACC**CAAACAC**CTGCGGCGGCGCT	-1,+7
AsuMf-G0-1.7-15	AATGAAAAATTCCTCCCCGGGACCA**TACGTAGCTTTTTGTT**CTGCGGCGGCGCT	+16
AsuMf-G0-1.8-13	AATGAAAAATTCCTCCCCGGGACC**TAAATTCGGACCCCGGGAC**CTGCGGCGGCGCT	-1,+19
AsuMf-G0-1.10-1	AATGAAAAATTCCTCCCCGGGACC**CCCCC**CGGCGGCGCT	-4,+5
AsuMf-G0-1.10-4	AATGAAAAATTCCTCCCCGGGAC---TGCGGCGGCGCT	-3
AsuMf-G0-1.11-2	AATGAAAAATTCCTCCCCGGGAC-----CGGCGGCGCT	-5
AsuMf-G0-1.11-3	AATGAAAAATTCCTCCCCGGGAC--CTGCGGCGGCGCT	-2
AsuMf-G0-1.11-11	AATGAAAAATTCCTCCCCGGGAC**GGCGG**TGCGGCGGCGCT	-3,+5
AsuMf-G0-1.16-2	AATGTTTTTGTTTTTGT---------TGCGGCGGCGCT	-22,+13
AsuMf-G0-1.16-9	AATGAAAAATTCCTCCCCGGGACC--TGCGGCGGCGCT	-2
AsuMf-G0-1.16-14	AATGAAAAATTCCTCCCCGGGACC**GG**CTGCGGCGGCGCT	-1,+2
AsuMf-G0-1.19-2	AATGAAAAATTCCTCCCCGGG-------CGGCGGCGCT	-7
AsuMf-G0-1.19-5	AATGAAAAATTCCTCCCCGGGAC**GGCGC**CGGCGGCGCT	-5,+5
AsuMf-G0-1.19-4	AATGAAAAATTCCTCCCCGGGACC**GGC**GCGGCGGCGCT	-3,+3
AsuMf-G0-1.19-6	AATGAAAAATTCCTCCCCGGGACCA**AA**TGCGGCGGCGCT	-1,+2
AsuMf-G0-1.19-7	AATGAAAAATTCCTCCCCGGGACCA**AA**TGCGGCGGCGCT	-1,+2
AsuMf-G0-1.19-8	AATGAAAAATTCCTCCTGCC-------GCGGCGGCGCT	-8
AsuMf-G0-1.19-10	AATGAAAAATTCCTCCCCGGGACC--TGCGGCGGCGCT	-2
AsuMf-G0-1.20-1	AATGAAAAATTCC-----------**GTA**GCGGCGGCGCT	-14,+3
AsuMf-G0-1.20-5	AATGAAAAACTCCTCCCCGGGACC--TGCGGCGGCGCT	-2
AsuMf-G0-1.20-6	AATGAAAAATTCCTCCCCGGGAC--CTGCGGCGGCGCT	-2
AsuMf-G0-1.20-7	AATGAAAAATTCCTCCCCGG--------CGGCGGCGCT	-8
AsuMf-G0-1.20-8	AATGAAAAATTCCTCCCCGGGAC**GCTGCGGGCGG**ACTGCGGCGGCGCT	-1,+11
AsuMf-G0-1.20-9	AATGAAAAATTCCTCCCCGGGACCA**A**CTGCGGCGGCGCT	+1
AsuMf-G0-1.20-11	AATGAAAAATTCCTCCCCGGGA------CGGCGGCGCT	-6

Supplementary Figure 5. Cas9/sgRNA-induced insertion and deletion mutations in *AsuMf*. PCR products spanning the sgRNA target site were analyzed for indel mutations. The first line represents the wild-type sequence, and subsequent lines show individual mutant clones. Deleted bases are marked with dashes and inserted or substituted bases are indicated in blue. The protospacer adjacent motif (PAM) sequences were marked with red. We use *AsuMf*$^{-}$ G0-x.y–z to specify the particular mutant sequence: x refers to the experiment number; y is the individual number; z is the clone number.

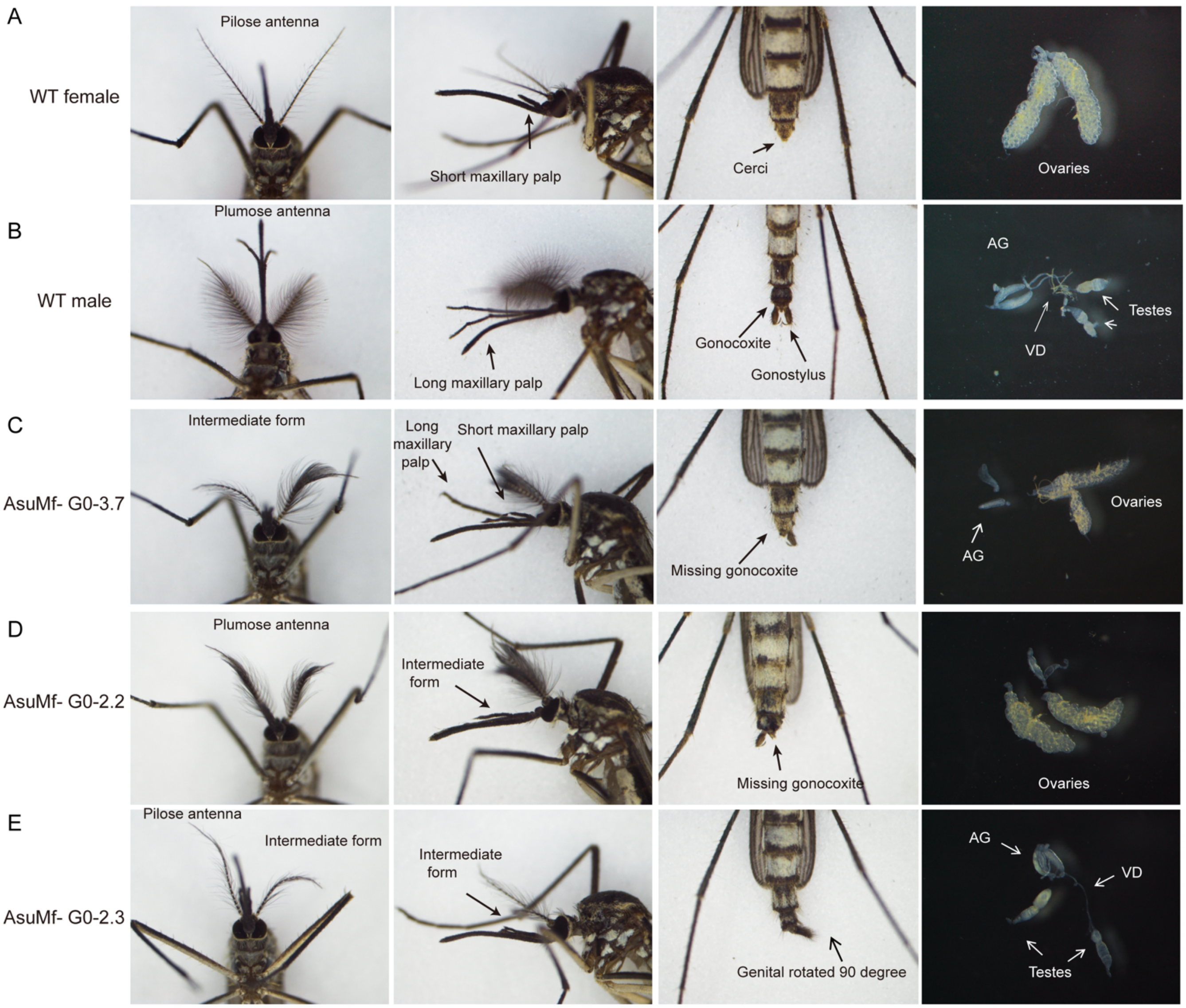

Supplementary Figure 6. Representative morphological phenotypes of wild-type male and female and *AsuMf*$^{-}$ individuals as well as mosaically-feminized. (A) WT female: pilose antenna, short maxillary palp, genitalia with cerci, and internal reproductive with ovaries. (B) WT male: plumose antenna, long maxillary palp, genitalia with gonocoxite and gonostyli, internal reproductive organs with AG, VD, and testes. (C) *AsuMf*$^{-}$ G0-3.7: both sides have fewer setae than WT males. One side of the maxillary palp was shorter than WT males. One side lacks a gonocoxite, and ovaries are present. (D) *AsuMf*$^{-}$ G0-2.2: plumose antenna; both side maxillary palp was shorter than WT male. One side lacks a gonocoxite. Internal reproductive organs with ovaries. (E) *AsuMf*$^{-}$ G0-2.3: both sides have fewer setae than WT males. Two sides of the maxillary palp were shorter than WT males. Genitalia rotated 90 degrees. Internal reproductive organs with AG, VD, and testes. AG, accessory glands; VD, vas deferens. Similar results were obtained in three independent experiments. Source data are provided as a Source Data file.

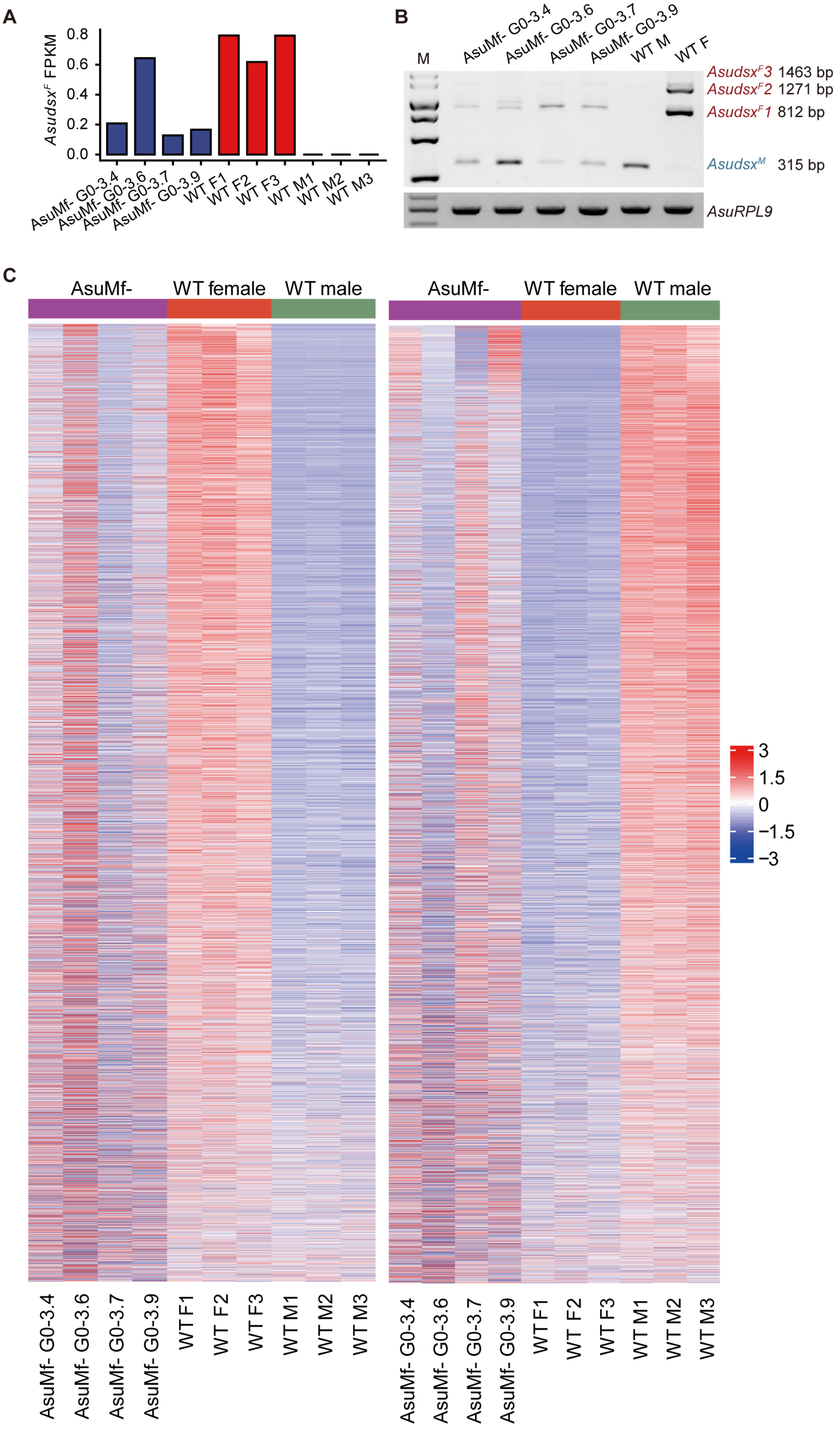
A
B
C
Asudsx^F FPKM
0.8
0.6
0.4
0.2
0.0
AsuMf- G0-3.4
AsuMf- G0-3.6
AsuMf- G0-3.7
AsuMf- G0-3.9
WT F1
WT F2
WT F3
WT M1
WT M2
WT M3
M
WT M
WT F
Asudsx^F3 1463 bp
Asudsx^F2 1271 bp
Asudsx^F1 812 bp
Asudsx^M 315 bp
AsuRPL9
AsuMf-
WT female
WT male
3
1.5
0
−1.5
−3

Supplementary Figure 7. Sex-biased gene expression in partially feminized *AsuMf*$^-$ mosaic males and wild-type *Ar. subalbatus*. (A) The RPKM of RNA-seq alignments to *Asudsx* female-specific from wild-type (WT) males (M) and females (F) and partially feminized *AsuMf*$^-$ mosaic males. *AsuMf*$^-$ G0-3.x: x is the numbering used to indicate the individual mosquito in experiment 3 of knock-out with CRISPR/Cas9. (B) Primers complementary to the *Asudsx* sex-specific exon 5 and exon 8 could amplify female *Asudsx* in partially feminized *AsuMf*$^-$ mosaic males, but not in WT males. (C) A heat map of log2 RPKM sex-biased transcript accumulation for all male and female-biased genes in wild-types (WT) males and females, and four *AsuMf*$^-$ individuals. Many male-biased genes are downregulated and female-biased gene are up-regulated in the partially feminized *AsuMf*$^-$ mosaic males. *AsuMf*$^-$ G0-3.x: x is the numbering used to indicate the individual mosquito in experiment 3 of knock-out with CRISPR/Cas9. WT, wild type. M, male. Similar results were obtained in three independent experiments. Source data are provided as a Source Data file.

E-value: 9e-176; Identities: 232/281(83%); Positives: 255/281(90%); Gaps: 2/281(0%)

```
AsuMf    64   RGNHRNIDDDKDQVFKRRRSGPGEEFFINKKLRMLQGPLTDISPIETEENKFFGRNRLFI  123
              RGN R +  D DQ F RRRSGPGE++FIN+KLRMLQGPL DI PIE+EE KF GRNRL+I
AsuHrp65 133  RGNRRTMGGDNDQGFDRRRSGPGEQYFINEKLRMLQGPLMDIPPIESEEAKFSGRNRLYI  192

AsuMf    124  GNLTNDATEDELIELFRPFGDISEIFMNKDKNYAFVRVDYFSNAVKAKRELEGTLHKNRM  183
              GNLTND TEDEL ELFRP+GDISEIFMNKDKNYAFVRVDYFSNA KAKRELEGT+ KNRM
AsuHrp65 193  GNLTNDVTEDELTELFRPYGDISEIFMNKDKNYAFVRVDYFSNAEKAKRELEGTMRKNRM  252

AsuMf    184  LRLRFAPSATIIRVRNLTPWVSDELLFKSFEVFGSVERAFVHVDERGKSTGKGIVEFKNK  243
              LR+RFAP+AT IRVRNLTPWVS+ELLFKSFEVFG VERA VHVDERGKSTG+GIVEFKNK
AsuHrp65 253  LRVRFAPNATAIRVRNLTPWVSNELLFKSFEVFGPVERASVHVDERGKSTGEGIVEFKNK  312

AsuMf    244  PAALVALRYCTDKCYFLTASLRPVIVEPYTYKDDS--VPEKSMNKKHPDFYKARQKGPRF  301
              P A+VALRYCT+KCYFLTASLRPVIVEPYTY+DD+  +PEKSMNKK PDF KARQ GPRF
AsuHrp65 313  PGAMVALRYCTEKCYFLTASLRPVIVEPYTYQDDTDGLPEKSMNKKIPDFQKARQHGPRF  372

AsuMf    302  AEYSSFEHEYGQRWKQLYELYRQKAEALKREMIMEEEKLEA  342
              A++ SFEHEYGQRWKQ++ELY+QKAE+LKREMIMEEEKLE
AsuHrp65 373  ADHGSFEHEYGQRWKQMHELYKQKAESLKREMIMEEEKLEG  413
```

Supplementary Figure 8. The central regions of *AsuMf* and *AsuHrp65* are highly conserved. Alignment was performed with Blastp using the default parameters. The two proteins show high similarity in their center regions with 83% identities (E-value: 9e-176). "+", a frequent substitution.

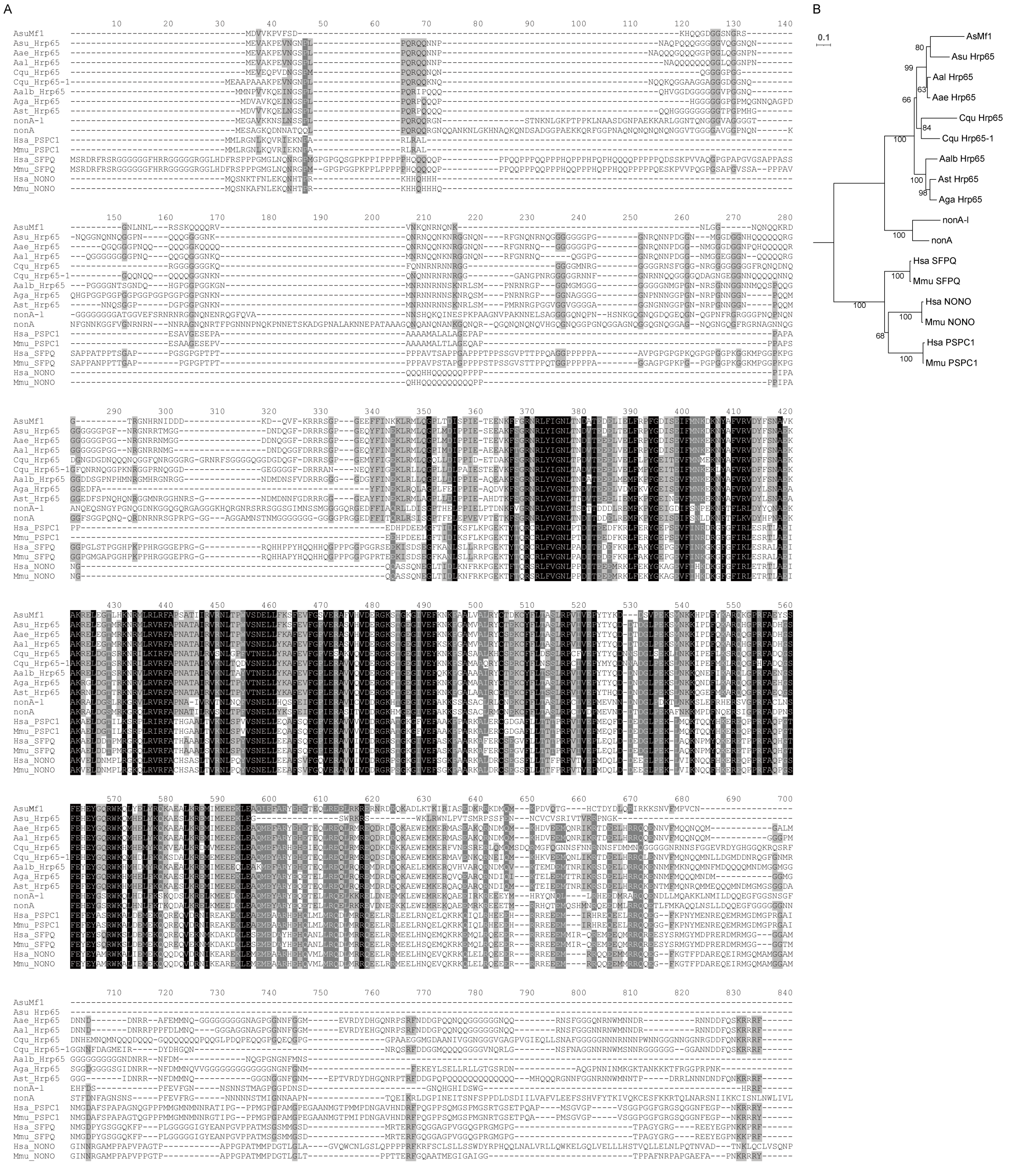

Supplementary Figure 9. ***AsuMf*** **alignment with DBHS homologs and phylogenetic inference.** (A) MUSCLE protein alignment of *AsuMf1* with its paralog *AsuHrp65*, and 15 other DBHS homologs, which were identified by Blastp and hmmsearch (All protein sequences attached in Supplementary Text3: DBHS Protein Sequences). (B) The gaps of alignment were trimmed by TrimAl with a parameter of "-gt 0.6 -cons 60" for

further phylogenetic inference (*37*). The phylogenetic tree of the seventeen trimmed sequences was analyzed using a Maximum-likelihood inference by IQ-TREE 2 (v2.0.3) program (see Fig. 4b) and a Neighbor-joining inference with Jukes-Cantor Neighbor-Joining method by Mega X. These two phylogenetic trees were resampled with 1000 Bootstrap replications.

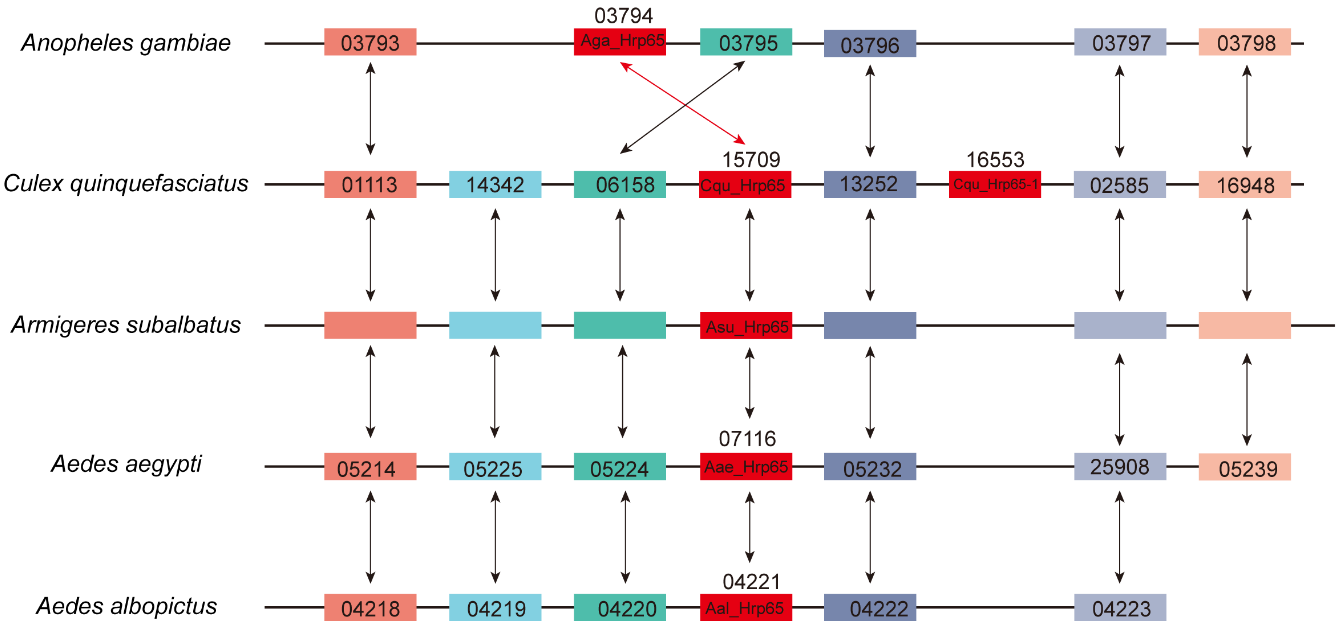

Supplementary Figure 10. Synteny analysis supports the conclusion that the autosomal *AsuHrp65* gene is ancestral compared to its *AsuMf* paralog. Synteny of genes flanking *AsuHrp65*, the paralog of *AsuMf*, is nearly perfectly maintained in *An. gambiae*, *Cx. quinquefasciatus*, *Ar. subalbatus*, *Ae. aegypti* and *Ae. albopictus,* supporting the conclusion that *AsuHrp65* is ancestral while *AsuMf* is derived. Genes of the same color connected by double-headed arrows are orthologs assigned by OrthoFinder (*40*). Gene names are preceded by the VectorBase convention, *An. gambiae* as AGAP0*,* *Cx. quinquefasciatus* as CQUJHB0, *Ae. aegypti* as AAEL0, and *Ae. albopictus* as AALF0, respectively.

Supplementary Table 1. CQs of the 21 transcripts sequences in three replicates of *Ar. subalbatus*.[1]

Transcript ID[2]	Length[3]	F1	M1	CQ 1	F2	M2	CQ 2	F3	M3	CQ 3	CQ mean	M mean	F mean
TRINITY_DN10587_c0_g4_i5[4]	1769	0	50	0.00	4	126	0.03	3	76	0.04	0.02	84.00	2.33
TRINITY_DN242886_c0_g1_i1	625	4	33	0.12	0	17	0.00	0	20	0.00	0.04	23.33	1.33
TRINITY_DN29360_c1_g1_i1	476	0	17	0.00	0	13	0.00	8	32	0.25	0.08	20.67	2.67
TRINITY_DN29419_c0_g2_i2	299	2	15	0.13	2	19	0.11	1	29	0.03	0.09	21.00	1.67
TRINITY_DN9342_c0_g5_i1	961	11	37	0.30	0	40	0.00	0	35	0.00	0.10	37.33	3.67
TRINITY_DN249382_c0_g1_i1	541	10	70	0.14	1	10	0.10	4	40	0.10	0.11	40.00	5.00
TRINITY_DN49156_c0_g1_i2	1302	9	86	0.10	3	37	0.08	8	39	0.21	0.13	54.00	6.67
TRINITY_DN60815_c1_g1_i1	663	14	43	0.33	4	42	0.10	0	23	0.00	0.14	36.00	6.00
TRINITY_DN18539_c1_g1_i2	805	21	95	0.22	6	74	0.08	16	114	0.14	0.15	94.33	14.33
TRINITY_DN11729_c0_g1_i3	952	16	49	0.33	9	73	0.12	0	85	0.00	0.15	69.00	8.33
TRINITY_DN52857_c0_g4_i1	360	1	9	0.11	5	28	0.18	8	44	0.18	0.16	27.00	4.67
TRINITY_DN40006_c0_g4_i1	472	4	28	0.14	4	11	0.36	0	44	0.00	0.17	27.67	2.67
TRINITY_DN52968_c0_g2_i1	755	21	54	0.39	1	56	0.02	6	55	0.11	0.17	55.00	9.33
TRINITY_DN116469_c0_g3_i3	550	0	13	0.00	7	31	0.23	9	30	0.30	0.18	24.67	5.33
TRINITY_DN21178_c1_g1_i2	399	2	44	0.05	4	25	0.16	9	27	0.33	0.18	32.00	5.00
TRINITY_DN9342_c0_g5_i2	1302	19	48	0.40	0	56	0.00	6	37	0.16	0.19	47.00	8.33
TRINITY_DN13328_c0_g3_i1	779	0	20	0.00	6	18	0.33	6	26	0.23	0.19	21.33	4.00
TRINITY_DN35302_c0_g1_i6	727	7	27	0.26	0	28	0.00	7	22	0.32	0.19	25.67	4.67
TRINITY_DN173471_c0_g1_i1	402	9	32	0.28	4	29	0.14	4	25	0.16	0.19	28.67	5.67
TRINITY_DN101511_c0_g1_i1	677	10	17	0.59	0	24	0.00	0	33	0.00	0.20	24.67	3.33
TRINITY_DN14205_c2_g1_i1	609	8	32	0.25	5	35	0.14	5	25	0.20	0.20	30.67	6.00

1 All transcripts were identified as having CQs < 0.2 in three replicates (CQ1, blue shade; CQ2, pink shade; CQ3. gray shade).
2 The transcripts were generated by Trinity assembly.
3 Length in nucleotides.
4 *AsuMf*
Abbreviations: F, female; M, male; CQ, chromosome quotients; CQ mean; M mean, mean CQ of three male replicates; F mean, mean CQ of three female replicates.

Supplementary Table 2. Male candidate genes mapped to the NCBI non-redundant database using Blastp. Five candidates were excluded based on similarities with transposases and reverse transcriptases.

Transcript ID	Description	Query Cover	E-value	Identity	Accession
TRINITY_DN116469_c0_g3_i3	putative RNA-directed DNA polymerase from transposon BS [*Blattella germanica*]	42%	0.004	41.56	PSN45577.1
TRINITY_DN101511_c0_g1_i1	hypothetical protein RP20_CCG000451 [*Aedes albopictus*]	63%	5e-11	32.21	KXJ69026.1
TRINITY_DN11729_c0_g1_i3	RNA-directed DNA polymerase from mobile element jockey [*Trichonephila clavipes*]	57%	2e-04	26.96	GFW33140.1
TRINITY_DN60815_c1_g1_i1	hypothetical protein RP20_CCG023641 [*Aedes albopictus*]	57%	4e-05	35.43	KXJ70435.1
TRINITY_DN21178_c1_g1_i2	endonuclease-reverse transcriptase [*Bombyx mori*]	66%	9e-07	28.57	ADI61823.1

Supplementary Table 3. *AsuMf* mapped to NCBI non-redundant database using Blastp. *AsuMf* peptide is the query, NCBI non-redundant database as the reference. Hrp65 and a splicing factor show the lowest e-values and highest query cover and identity.

Description	Query Cover	E-value	Identity	Accession
hrp65 protein isoform X3 [*Aedes aegypti*]	82%	3.00E-179	76.99	XP_021705282.1
protein no-on-transient A isoform X3 [*Aedes albopictus*]	81%	1.00E-177	76.7	XP_029728793.1
hrp65 protein isoform X2 [*Aedes albopictus*]	81%	1.00E-177	76.7	XP_029728788.1
hrp65 protein-like isoform X2 [*Anopheles stephensi*]	84%	4.00E-149	64.82	XP_035890879.1
AGAP003794-PB-like protein [*Anopheles sinensis*]	78%	3.00E-148	68.06	KFB41515.1
hrp65 protein-like isoform X2 [*Anopheles arabiensis*]	84%	4.00E-147	64.72	XP_040175595.1
hrp65 protein-like isoform X2 [*Anopheles merus*]	84%	1.00E-146	64.44	XP_041762709.1
hrp65 protein-like isoform X3 [*Anopheles albimanus*]	78%	4.00E-146	68.66	XP_035779301.1
hrp65 protein-like [*Anopheles coluzzii*]	84%	2.00E-145	64.44	XP_040219649.1
hrp65-1 [*Anopheles darlingi*]	78%	2.00E-142	67.46	ETN64374.1
AGAP003794-PB [*Anopheles gambiae* str. PEST]	78%	3.00E-142	65.59	XP_003436621.1
NONA protein [*Culex quinquefasciatus*]	75%	2.00E-136	65.11	EDS27331.1
protein no-on-transient A-like [*Culex pipiens pallens*]	74%	1.00E-133	65.09	XP_039432198.1
protein no-on-transient A [*Culex quinquefasciatus*]	74%	1.00E-133	65.09	XP_001847991.2
splicing factor [*Culex quinquefasciatus*]	74%	2.00E-133	65.09	EDS27332.1

Supplementary Table 4. NCBI conserved domains found in *AsuMf1-3*. Top matches are to the NOPS domain and RNA recognition motif (RRM), and contain the RRM2_SXL, an RNA recognition motif 2 (RRM2) found in *Drosophila* sex-lethal (SXL) and similar proteins.

Name	Accession	Description	Interval	E-value
NOPS_NONA_like	cd12945	NOPS domain, including C-terminal coiled-coil region, in p54nrb/PSF/PSP1 homologs from invertebrate species; The family contains a DBHS domain (for Drosophila behavior, human splicing), which comprises two conserved RNA recognition motifs (RRMs), also termed RBDs (RNA binding domains) or RNPs (ribonucleoprotein domains), and a charged protein-protein interaction NOPS (NONA and PSP1) domain.	264-361	1.96E-55
RRM2_p54nrb_like	cd12333	RNA recognition motif 2 (RRM2) found in the p54nrb/PSF/PSP1 family;	194-272	1.03E-36
RRM1_p54nrb_like	cd12332	RNA recognition motif 1 (RRM1) found in the p54nrb/PSF/PSP1 family;	118-188	2.77E-25
NOPS	pfam08075	NOPS (NUC059) domain; This domain is found at the C-terminus of NONA and PSP1 proteins adjacent to 1 or 2 pfam00076 domains.	266-316	6.58E-21
RRM	smart00360	RNA recognition motif;	120-186	1.83E-14
PABP-1234	TIGR01628	polyadenylate binding protein, human types 1, 2, 3, 4 family;	121-251	1.64E-13
RRM_1	pfam00076	RNA recognition motif. (a.k.a. RRM, RBD, or RNP domain); The RRM motif is probably diagnostic of an RNA binding protein.	121-185	3.41E-13
RRM_1	pfam00076	RNA recognition motif. (a.k.a. RRM, RBD, or RNP domain); The RRM motif is probably diagnostic of an RNA binding protein.	195-251	6.39E-10
RRM	smart00360	RNA recognition motif;	195-253	1.70E-08
RRM	COG0724	RNA recognition motif (RRM) domain [Translation, ribosomal structure and biogenesis];	111-192	2.21E-06
rad50	TIGR00606	rad50;rad50; All proteins in this family for which functions are known are involved in recombination, recombinational repair, and/or non-homologous end joining.	313-391	8.67E-03
RRM3_TIA1_like	cd12354	RNA recognition motif 2 (RRM2) found in granule-associated RNA binding proteins (p40-TIA-1 and TIAR), and yeast nuclear and cytoplasmic polyadenylated RNA-binding protein PUB1; This subfamily corresponds to the RRM3 of TIA-1, TIAR, and PUB1.	122-185	1.75E-12
RRM2_SXL	cd12651	RNA recognition motif 2 (RRM2) found in Drosophila sex-lethal (SXL) and similar proteins; This subfamily corresponds to the RRM2 of the sex-lethal protein (SXL) which governs sexual differentiation and X chromosome dosage compensation in Drosophila melanogaster.	121-189	2.83E-03

Supplementary Table 5. Sequence of the sgRNA used in this study		
**sgRNA Name**	**sgRNA sequence**	**Cleavage site in *AsuMf* cDNA**
AsuMf-sgRNA1	ATTCCTCCCCGGGACCACTG	364-365

Supplementary Text1. DBHS Nucleotide Sequences.

>***AsuMf1***
GGTGCGAATATTGAATTCAAATTTTGGGTTGATTTATTTGTCCGTGTACCGTGCCCGGTTCGATTTTTTCTGTCCCCTCGTAAATTTTTTCGTCCGGTTCACAATTTACGAT
CGCAATGGATGTTGTGAAGCCAGTGTTTAGTGATAAACATCAACAAGGTGACGGCGGCAGCAATGGCCGAAGTGGAAACCTGAATAACCTTCGAAGCTCTAAACAGCAGCAG
CAGAGAGTAGTAAATAAGCAAAACAGAAATCAAAATAAAAATCTTGGCGGAAATCAAAACCAGCAAAAGCGCGATGGCACTCGAGGAAACCATCGAAATATCGACGACGACA
AGGACCAAGTTTTTAAGCGCCGCCGCAGTGGTCCCGGGGAGGAATTTTTCATTAACAAAAAGCTACGTATGCTCCAAGGACCGCTGACTGACATTTCTCCCATTGAAACTGA
GGAAAACAAATTCTTCGGCCGTAATCGTCTGTTCATAGGCAATCTTACCAACGATGCTACAGAAGATGAGCTTATTGAACTGTTTAGACCGTTCGGTGACATAAGTGAAATC
TTTATGAATAAAGACAAGAATTACGCCTTTGTGCGCGTAGATTATTTCTCTAATGCCGTGAAGGCCAAACGGGAGCTTGAAGGAACCTTGCACAAGAACCGCATGCTTCGAT
TGCGCTTTGCTCCAAGTGCTACTATTATTCGCGTTCGCAATCTAACTCCATGGGTCAGCGACGAGTTGCTGTTCAAGTCTTTTGAGGTGTTTGGCTCCGTAGAACGTGCTTT
CGTGCATGTTGATGAGCGTGGAAAATCAACTGGTAAAGGTATCGTCGAATTCAAGAACAAACCTGCAGCCTTGGTCGCTTTAAGGTATTGCACGGATAAATGTTATTTCTTG
ACGGCATCCCTACGACCAGTCATTGTTGAACCCTACACCTATAAGGATGACTCTGTTCCGGAGAAATCCATGAATAAGAAACATCCGGATTTTTATAAGGCCAGACAGAAAG
GACCGCGCTTTGCGGAGTATAGCTCCTTTGAGCATGAGTACGGCCAGCGCTGGAAGCAGTTGTACGAATTGTACAGGCAGAAAGCAGAAGCATTGAAGCGCGAAATGATAAT
GGAAGAGGAAAAATTAGAAGCTCAGATAGAATTCGCCCGTTACGAGCACGAAACCGAGCAACTTCGAGAAGAATTACGTAAGCGGGAGCGGAACCGTGACCGTCAGAAGGCC
GACTTGAAAACAAAGATACGGATCGCTTCTGAAGATAAACGACGTAAAGATATGCAGATGAAACCAGATGTCCAAACAGGGCACTGCACTGATTATGATCTCCAGCGTATTC
GAAAGAAAAGCAACGTTTTTATGCCGGTTTGTAATTAAAATAAAGTTAGCACGGCGACTTATTTTTTTTAATAGTTAGATTTAATAACATTATTGTTCTCACAACAGAATCA
GAATTCAGCTTTCCGCGAGCGGTAACGGCCCCCAGCTTGCCTGTGGGCTAGTCTTTGTTTTGACGTTTTTGGAGATCAACTGCACCCACCGTTCCGAGCATTCTTATCAATG
TGGTATATAAGAAGGCTTGAATTCACTTCATCAGCACCTTCTTATATGCTACATGGGTCAGAATACTTGGAATATATACACATGGGTCAGAATATACCGCTCGTGCGGTGGG
TAAGGTTTGTTGAATTGCTACGATATTTGTGCACTTGCATCCTTTTGCTGGAGTTTTGGCCCACGCTCCCCTCACCATAGATGCATAAAGTGAAAACAATAGAAAACCGTAA
ATGTATTCAGTTTACATCCAATCGATTACCAATAAACAGTGGTTTTATTGACGGG

>***AsuMf2***
GGTGCGAATATTGAATTCAAATTTTGGGTTGATTTATTTGTCCGTGTACCGTGCCCGGTTCGATTTTTTCTGTCCCCTCGTAAATTTTTTCGTCCGGTTCACAATTTACGAT
CGCAATGGATGTTGTGAAGCCAGTGTTTAGTGATAAACATCAACAAGGTGACGGCGGCAGCAATGGCCGAAGTGGAAACCTGAATAACCTTCGAAGCTCTAAACAGCAGCAG
CAGAGAGTAGTAAATAAGCAAAACAGAAATCAAAATAAAAATCTTGGCGGAAATCAAAACCAGCAAAAGCGCGATGGCACTCGAGGAAACCATCGAAATATCGACGACGACA
AGGACCAAGTTTTTAAGCGCCGCCGCAGTGGTCCCGGGGAGGAATTTTTCATTAACAAAAAGCTACGTATGCTCCAAGGACCGCTGACTGACATTTCTCCCATTGAAACTGA
GGAAAACAAATTCTTCGGCCGTAATCGTCTGTTCATAGGCAATCTTACCAACGATGCTACAGAAGATGAGCTTATTGAACTGTTTAGACCGTTCGGTGACATAAGTGAAATC
TTTATGAATAAAGACAAGAATTACGCCTTTGTGCGCGTAGATTATTTCTCTAATGCCGTGAAGGCCAAACGGGAGCTTGAAGGAACCTTGCACAAGAACCGCATGCTTCGAT
TGCGCTTTGCTCCAAGTGCTACTATTATTCGCGTTCGCAATCTAACTCCATGGGTCAGCGACGAGTTGCTGTTCAAGTCTTTTGAGGTGTTTGGCTCCGTAGAACGTGCTTT
CGTGCATGTTGATGAGCGTGGAAAATCAACTGGTAAAGGTATCGTCGAATTCAAGAACAAACCTGCAGCCTTGGTCGCTTTAAGGTATTGCACGGATAAATGTTATTTCTTG
ACGGCATCCCTACGACCAGTCATTGTTGAACCCTACACCTATAAGGATGACTCTGTTCCGGAGAAATCCATGAATAAGAAACATCCGGATTTTTATAAGGCCAGACAGAAAG
GACCGCGCTTTGCGGAGTATAGCTCCTTTGAGCATGAGTACGGCCAGCGCTGGAAGCAGTTGTACGAATTGTACAGGCAGAAAGCAGAAGCATTGAAGCGCGAAATGATAAT
GGAAGAGGAAAAATTAGAAGCTCAGATAGAATTCGCCCGTTACGAGCACGAAACCGAGCAACTTCGAGAAGGTATGTTTTTTTTCAGTTTCAAAAGGTATGTTTTTGCCCTT
CTCGTACAGAAAGTTCCCAGTCAACATTTTGGTACCTATAACTCTTGATATAGGTTATTATATAAGAACCAACTTAATCGTATATAATGCACAGATTGCCTATATACGTACC
AAAGTGGAGGCGATATAGGTACATACGAGTTTTATTTTACGATTTTTCCCGACATCAAAAGAACATTATTACGATTAAACATTAAACATG

>***AsuMf3***
GGTGCGAATATTGAATTCAAATTTTGGGTTGATTTATTTGTCCGTGTACCGTGCCCGGTTCGATTTTTTCTGTCCCCTCGTAAATTTTTTCGTCCGGTTCACAATTTACGAT
CGCAATGGATGTTGTGAAGCCAGTGTTTAGTGATAAACATCAACAAGGTGACGGCGGCAGCAATGGCCGAAGTGGAAACCTGAATAACCTTCGAAGCTCTAAACAGCAGCAG
CAGAGAGTAGTAAATAAGCAAAACAGAAATCAAAATAAAAATCTTGGCGGAAATCAAAACCAGCAAAAGCGCGATGGCACTCGAGGAAACCATCGAAATATCGACGACGACA
AGGACCAAGTTTTTAAGCGCCGCCGCAGTGGTCCCGGGGAGGAATTTTTCATTAACAAAAAGCTACGTATGCTCCAAGGACCGCTGACTGACATTTCTCCCATTGAAACTGA
GGAAAACAAATTCTTCGGCCGTAATCGTCTGTTCATAGGCAATCTTACCAACGATGCTACAGAAGATGAGCTTATTGAACTGTTTAGACCGTTCGGTGACATAAGTGAAATC
TTTATGAATAAAGACAAGAATTACGCCTTTGTGCGCGTAGATTATTTCTCTAATGCCGTGAAGGCCAAACGGGAGCTTGAAGGAACCTTGCACAAGAACCGCATGCTTCGAT
TGCGCTTTGCTCCAAGTGCTACTATTATTCGCGTTCGCAATCTAACTCCATGGGTCAGCGACGAGTTGCTGTTCAAGTCTTTTGAGGTGTTTGGCTCCGTAGAACGTGCTTT
CGTGCATGTTGATGAGCGTGGAAAATCAACTGGTAAAGGTATCGTCGAATTCAAGAACAAACCTGCAGCCTTGGTCGCTTTAAGGTATTGCACGGATAAATGTTATTTCTTG
ACGGCATCCCTACGACCAGTCATTGTTGAACCCTACACCTATAAGGATGACTCTGTTCCGGAGAAATCCATGAATAAGAAACATCCGGATTTTTATAAGGCCAGACAGGTAA
GCTATCTTTATAAATAATGTACGTTTGATTATTCTACTGGTTATTGTTCTCGTTTTGTTTCCGTATTTCATAACAGAAAGGACCGCGCTTTGCGGAGTATAGCTCCTTTGAG
CATGAGTACGGCCAGCGCTGGAAGCAGTTGTACGAATTGTACAGGCAGAAAGCAGAAGCATTGAAGCGCGAAATGATAATGGAAGAGGAAAAATTAGAAGCTCAGATAGAAT
TCGCCCGTTACGAGCACGAAACCGAGCAACTTCGAGAAGGTATGTTTTTTTTCAGTTTCAAAAGGTATGTTTTTGCCCTTCTCGTACAGAAAGTTCCCAGTCAACATTTTGG
TACCTATAACTCTTGATATAGGTTATTATATAAGAACCAACTTAATCGTATATAATGCACAGATTGCCTATATACGTACCAAAGTGGAGGCGATATAGGTACATACGAGTTT
TATTTTACGATTTTTCCCGACATCAAAAGAACATTATTACGATTAAACATTAAACATG

>***AsuMf4***
GGTGCGAATATTGAATTCAAATTTTGGGTTGATTTATTTGTCCGTGTACCGTGCCCGGTTCGATTTTTTCTGTCCCCTCGTAAATTTTTTCGTCCGGTTCACAATTTACGAT
CGCAATGGATGTTGTGAAGCCAGTGTTTAGTGATAAACATCAACAAGGTGACGGCGGCAGCAATGGCCGAAGTGGAAACCTGAATAACCTTCGAAGCTCTAAACAGCAGCAG
CAGAGAGTAGTAAATAAGCAAAACAGAAATCAAAATAAAAATCTTGGCGGAAATCAAAACCAGCAAAAGCGCGATGGCACTCGAGGAAACCATCGAAATATCGACGACGACG
TAAGTACCTGGTTTTGTCCAGAGTATATGTAATTTTGTAATAGTACACTGGTTTTGGCGTGCACGCTTAAGCCAATCTGTTACAGTCTCTCGTCTCTCGGGAACAAGAAAAG
GTGCATGCACGGAGAAATAGGAACACTCAGAAGTGGGTATTTTTTCACCCACCTTCGAGATAAGTGGACTAACCCATAAGGTGGGTAAAGTGGCTTTACCCATAAATGGGAA
GAAGGCTTGTACGTCAAAACAGATGTGTATTTTTTACTCACCTTCTCGAATAAAAAAATTCTTTTGGATGGGTGAAAAACACCCATCTGCCGTCAAATAATTTCAGCGCTGA
TGATTGTTGCGAAATAAATAATTTTAAAGAAATGGGCGTACAACATTAAACGTATGTATTTATTACTCCTCTTATTGTATACATGACACAAGTTTAATTAACTAAACCAGCT
CAACTTATTGAAAATCACATGAAATAGTTAGCTATTTTTTGGAAATTCCAAAGAAGTTCTATTCGAGGTTCTAGTAGCATGCAATTTGATCGATCGAGGTCGTTCAGGTCTC
AATCCAGGCATCCAAATTACTTTACATTGTCCTCTGCTGTACTGTTTTTATGCACGAGCAATCCATCACGGAGTGATCTG

>***AsuHrp65***
GATTTCTGCGAAACGGGACTAGCATTTTCCTCATTCTGCTTTTGACTGTCGGCCGTATAACACGCTGTCACTGATTTTTGTAAAGTGAGTGAAAAATTAGAAGGGGAAAATT
TTTTAGAGGAAGTGCAGGAAAATCTAGTGCGTGAAGTGTCGTTATTATATAACGTAAATCCTCAACTGGTCATCAAATTACGATCGCAATGGAAGTTGCTAAGCCAGAAGTT
AACGGTAACCCACTGCCCCAGCGGCAGCAGAATAATCCCAATGCCCAGCCACAGCAGCAGGGCGGCGGCGGCGGTGTCCAAGGTGGAAATCAGAATAATCAAGGTGGAAACC
AGAATAACCAAGGCGGCCCCAATCAACAGCAGGGTGGAGGCGGAAACAAGCAGAACCGCAATCAACAAAATAAGAACCGTGGCGGAAATCAAAATCGCTTCGGCAATCAGAA
CCGCAATCAAGGCGGCGGTGGCGGCGGCGGCCCCGGCGGAAATCGTCAGAACAATCCAGACGGTGGCAATATGGGCGGCGACGGCGGTAATCACCAGCAACAGCAGCAGCAG
CGCGGTGGTGGCGGCGGCGGTGGCCCCGGTTTCAATCGTGGCAATCGTCGGACAATGGGCGGCGACAACGACCAAGGCTTCGACCGACGTCGCAGTGGGCCCGGAGAGCAGT
ACTTCATAAACGAGAAGCTGCGTATGCTGCAGGGACCGTTGATGGATATCCCCCCAATTGAATCGGAGGAGGCCAAATTCTCCGGTCGTAATCGTTTGTACATCGGCAATCT
GACCAACGATGTCACTGAAGATGAGCTAACCGAACTGTTCAGACCGTACGGTGACATCAGCGAGATCTTCATGAATAAGGACAAGAACTACGCCTTTGTGCGCGTAGATTAT
TTCTCGAATGCCGAGAAAGCCAAGCGCGAGCTTGAGGGAACCATGCGCAAGAACCGCATGCTTCGAGTGCGCTTTGCTCCAAATGCCACCGCTATTCGCGTTCGCAATCTCA

CTCCATGGGTCAGCAACGAGTTGCTGTTCAAGTCCTTTGAGGTGTTTGGTCCCGTGGAACGTGCCTCCGTGCATGTTGATGAACGTGGAAAATCGACCGGCGAAGGTATTGT
CGAATTCAAGAACAAACCAGGTGCCATGGTCGCGTTACGGTATTGCACAGAGAAGTGTTACTTCTTGACAGCGTCCCTAAGACCAGTCATTGTTGAACCCTACACCTATCAA
GATGACACCGATGGATTGCCAGAGAAATCCATGAACAAGAAAATTCCAGACTTCCAGAAAGCTAGACAGCACGGGCCACGCTTTGCGGATCATGGCTCCTTTGAGCATGAGT
ACGGGCAGCGCTGGAAGCAAATGCACGAGTTATACAAGCAGAAGGCTGAATCATTGAAGCGCGAAATGATCATGGAGGAAGAGAAGCTGGAAGGATCATGGAGGAAGAGAAG
CTGGAAGCTCAGATGGAATTTGCCCGTTACGAGCATGAGACCGAGCAGCTTCGAGAACAATTGCGTATGCGTGAGCAGGATCGTGACCGTCAGAAGGCCGAATGGGAAATGA
AGGAACGGATGGCCTCGGAAGCTAAACAACGTAATGATATACAGATGAAGCACGACGTCGAGGAAATGCAAAGCCGCATCAAGCGTACTGATGAGGAACTCCACCGTCGTCA
ACAGGAAAACAATGTTTTTATGCAGCAAAATCAGCAAATGGGTGGTCTAATGGATAACGATGACAATCGACGGCCATTTGAAATGAACCAAGGAAATGCAGGTGGTAACAGC
AATGCTGGCCCTGGTAACAACTTTGGCGGAATGGAGGTTCGCGATTATGATCACGGTCAGAACAGACCATCCCGCTTCAACAACGAAGATGGCCCACAGCAGAACCAACAAG
GAGGAGGCGGCGGCGGCGGCGCTGGTGGCAATCAGCAGCGTAACTTTGGAGGTGGAAACAATCGCAACTGGATGAACAATGACCGTCGTAACAATGACGACTTCCAGAGCAA
ACGAAGACGCTTCTAAAATGCTGATAACTGTTAAGGATCTCACTCATTACGTACCATTCTTCACAGACACAAGACAGTATTGATACTTGTGTTGCGCCTAGAGACGGGCGGA
TTTCAAATTTTACCAGCCCGAACCCAGATTTATTTTTAAATCATTAAACCCGGACCAGAAACGG

>**AaeHrp65 [AAEL017116-*RB] [Aedes aegypti***]
ATTCTGCTTTTGACTGTCGGCTGTGTTGCACGCTGTTAAAATTTCTCTGCAAGGTGTGTGTGAAAATTGGCAGTGAAAAGAAGGTGAAGTTTCTCAAGAGGAAAGTGGAAAA
AGTATTAATTCAGTGAACTGTGGAAGTGTCTTGTTATATAACGGAAATCCGCAGCTGGTAAACAGATTCAAACAACAATGGAAGTAGCAAAGCCGGAAGTCAATGGAAGCCC
ACTGCCACAGCGGCAGCAGAATAACCCTAATGCCCAACAGCAGGGACAGCAGCAGGGTGGCCCCGGCGGTCTTCAGGGAGGAAATCCCAACCAGGGCCAAGGTGGTGGCCCC
AATCAGCAGCAGCAGCAGGGTGGCAAACAACAACAGCAGCAGAACCGGAACCAGCAGAATAAGAATCGGGGCGGAAACCAGAATCGGTTCGGAAATCGCAACCAAGGAGGCG
GCGGCGGAGGCCCCGGCGGAAACCGTCAGAACAATCCAGATGGCGGAAATATGGGCGGCGGTGACGGTGGCAATCCGCAACATCAGCAGCAGCAACAGCGTGGAGGTGGCGG
AGGAGGCCCCGGTGGCAACCGTGGAAACCGCCGGAACATGGGTGGCGATGATAACCAAGGTGGTTTCGACCGCCGTCGCAGTGGCCCTGGAGAGCAGTACTTCATCAACGAG
AAGCTGCGCATGCTGCAGGGTCCCTTGATGGACATTCCCCCGATTGAAACCGAGGAGGTCAAATTCTCCGGCCGCAACCGGTTGTACATCGGTAACCTGACCAACGACATTA
CCGAAGAGGAGTTGGTTGAACTGTTCCGGCCGTACGGCGACATCAGTGAAGTATTCATGAACAAGGAGAAGAACTACGCCTTTGTGCGTGTAGATTACTTCTCCAATGCCGA
GAAGGCCAAGCGCGAGCTCGAGGGCACAATGCGCAAGAACCGCATGTTGCGAGTGCGCTTTGCACCCAATGCTACTGCCATTCGCGTCCGTAATCTCACTCCATGGGTCAGC
AACGAGCTGCTGTTCAAGGCTTTTGAAGTGTTTGGACCGGTAGAACGCGCCTCCGTGCATGTTGACGAACGCGGAAAATCAACTGGAGAAGGCATCGTCGAGTTCAAGAACA
AACCAGGAGCGATGGTCGCATTGCGGTATTGCAGCGAGAAATGCTTCTTCTTGACGGCGTCCCTGCGGCCAGTCATTGTTGAACCTTACACCTACCAGGATGACACCGATGG
TCTGCCAGAGAAATCCATGAACAAGAAAATTCCAGACTTCCAGAAGGCCAGACAGCAAGGACCACGTTTCGCTGATCACGGATCCTTCGAGCATGAGTACGGTCAACGGTGG
AAGCAAATGCACGAGTTGTACAAGCAGAAGGCCGAGGCTCTGAAGCGCGAAATGATCATGGAGGAGGAGAAATTGGAAGCTCAGATGGAATTCGCCCGCTACGAGCACGAGA
CCGAGCAACTCCGAGAACAGTTGCGCATGCGCGAGCAGGACCGTGACCGTCAAAAGGCCGAATGGGAAATGAAGGAACGCATGGCGTCGGAAGCCAAACAGCGCAATGACAT
GCAGATGAAGCACGACGTCGAGGAAATGCAAAACCGCATCAAGCGCACCGATGAGGAACTGCACCGTCGTCAGCAGGAGAACAACGTATTTATGCAGCAGAACCAACAGATG
GGTGCCCTAATGGACAACAACGATGACAACCGGCGGGCATTCGAAATGATGAACCAGGGTGGCGGTGGAGGAGGCGGCGGTGGCAACGCCGGACCCGGTGGTAACAACTTTG
GCGGCATGGAGGTACGCGATTATGACCATGGTCAGAACAGACCGTCCCGCTTCAACGACGATGGCCCGCAACAGAACCAGCAGGGAGGAGGCGGCGGTGGCAACCAGCAGCG
CAATTCCTTCGGCGGTGGACAAAATCGCAACTGGATGAACAATGATCGTCGTAACAATGATGATTTCCAGAGCAAACGAAGACGCTTCTAAAAAGCTTAAGGAATAACGTTA
AGTTCTCACTCACCGCCAACCAATCATTCTATACACACAAGCCAGCTCGTGGATGTGCTCTTGTGATGTGCGTCGTGGGCTGCGCTTTCTCCGTTAATAGCAGTAACATCCC
CATATTGGTTGGGAGGATTATCTCGTCAGTACAATGAACAAATTGTACTTCATCCTAGTTTCGTTTTATGTCCGTGACTCGTGAGACGCGACAGAATGTAAATCGTCTCGTC
GATAAATTAGTTCAAGAGGGGGAGAAAGAAAAGTATCAGGGTTATAAATCGAGAGAATTTCACGTTTATGGCTTATCTGACAAATCAATAACTATGAGAAAACAACCAATGA
AATTCTCAAGTGCGATTTTTGATTCCAATTGCAGAAAAAATTCTCAAACTATATTTCCTATTTCATAAACCTACTTTAAAAACTCCTATGAATACCACACATATATCCTACA
ATTTCCTTTGCTATTTCGTTCTAAAAAAATCTGTCGCCGAAAACGTTCTCGAATAAGTCAGTATCGCCAGTAATATGTCAGATAGTGTCTCTGGCAGAGAAATAATTTCCGA
AGCGTTTCATTTGTTTTGTCGTCAAAGAAAGTAAAAGGCAAGAATTTTGCATTATGAATACAAAGGGAGAAACACGGATACGCGCGGCACTGTAAACGGTGCGCGACTGACT
GTACCTTCCTTCCAATTCAATTTTAACTGAGAGAAAAATGAGAGAAACTGATTGTGTATTTGATGATCTAGAGATATGTAACACATATTATTTATAACCCTGATTTGGCTGA
TGGACAGAGGTAGGTTTTGCCTACCGTTGGCTCTGTGCGCACGCATACACAACTTGTATTTGCAGCAGTACTATTTATAAGGACAGAAAAATGGAGTTTTTTTTTAATCACC
AAGATTAAATCCTACGCGTAAGGCTTAAAGATTATATCTTTTTACAAATCACTGTGTAATAATTATAATAATATAAATGATATTCATTGGACACAATAA

>**AalHrp65 [AALF004221-RA]** ***[Aedes albopictus***]
ATGGAAGTAGCCAAGCCAGAGGTGAATGGAAGCCCACTGCCACAGCGGCAGCAGAATAACCCTAATGCCCAGCAACAGCAGCAGCAGCAAGGTGGCCTCCAAGGAGGTAACC
CTAACCAAGGTGGCGGCGGCGGCGGCGGTGGTCCCAACCAGCAGCAGGGACAGGGTGGTGGCAACAAGCAGATGAACCGCAACCAGCAGAACAAGAACCGCGGTGGAAACCA
GAACCGGTTCGGAAACCGCAACCAGCAGGGTGGACCCGGCGGAAACCGTCAGAATAACCCGGATGGCGGCAATATGGGCGGAGAGGGTGGCGGCAACCAGCAGCAGCAGCAA
CGCGGCGGTGGTGGCGGCGGCGGCCCCGGTGGCAACCGAGGAAACCGCCGGAACATGGGCGATAACGATCAGGGTGGTTTCGATCGCCGTCGCAGTGGCCCCGGAGAGCAGT
ACTTCATCAACGAGAAGCTGCGCATGCTGCAGGGACCAATCATGGACATTCCTCCGATTGAAGCCGAAGAGGCCAAATTCACCGGCCGCAATCGGTTGTACATCGGTAATCT
GACCAACGATGTAACGGAGGAAGAACTGGTCGAACTGTTCCGGCCGTACGGCGACATCAGCGAAATCTTCATGAACAAGGAGAAGAACTACGCCTTTGTGCGTGTAGACTAC
TTCTCCAACGCCGAGAAGGCCAAGCGCGAGTTGGAGGGAACGATGCGCAAGAACCGCATGCTGCGAGTGCGCTTTGCCCCCAACGCCACCGCCATTCGCGTTCGTAATCTCA
CTCCCTGGGTCAGCAACGAGCTGCTGTACAAAGCATTTGAGGTGTTCGGTCCGGTGGAACGTGCCTCTGTGCATGTTGACGAACGTGGAAAATCTACCGGCGAAGGCATCGT
CGAGTTCAAGAGCAAGCCTGGTGCAATGGTTGCACTGCGGTACTGTACCGAGAAATGCTTCTTCCTGACGGCGTCCCTGCGCCCGGTCATTGTTGAACCCTACACCTACCAG
GATGACACGGATGGTCTGCCAGAAAAGTCGATGAACAAGAAAATCCCAGACTTCCAGAAGGCCAGACAGCATGGACCACGTTTCGCCGACCATGGTTCGTTCGAGCATGAGT
ACGGTCAACGCTGGAAGCAAATGCACGAACTGTACAAGCAGAAGGCCGAGGCTCTGAAGCGCGAAATGATCATGGAGGAGGAGAAGCTGGAAGCTCAGATGGAATTCGCCCG
CTACGAGCACGAGACCGAGCAGCTCCGAGAACAGTTGCGCATGCGCGAGCAAGACCGTGACCGTCAGAAGGCCGAATGGGAAATGAAGGAACGTATGGCGTCGGAAGCCAAA
CAGCGCAACGACATGCAGATGAAGCACGACGTCGAGGAAATGCAGAACCGCATCAAGCGTACCGACGAGGAACTGCACCGCCGTCAGCAGGAGAACAATGTCTTTATGCAGC
AAAACCAGCAAATGGGTGGTGGTCCAATGGACAACAATGATGACAACCGGCGGCCGCCTCCGTTCGACTTGATGAACCAGGGCGGTGGTGCCGGCGGCAATGCCGGACCCGG
TGGTAACAACTTTGGCGGAATGGAGGTTCGCGATTATGAACATGGTCAGAACAGACCGTCCCGCTTCAACGACGATGGCCCGCAGCAGAACCAGCAAGGAGGAGGCGGCGGT
GGCAACCAGCAGCGTAATTCCTTCGGCGGTGGAAACAATCGCAACTGGATGAACAATGATCGTCGTAACAATGATGATTTCCAAAGCAAGCGAAGACGCTTCTAA

>**AgaHrp65 [AGAP003794-RB] [*Anopheles gambiae*]**
GCTCGTGGGAAACGGAAGCAAGTTGTTCGCTCATCGTGCGGTCGCTGATCGATTGGTGTGAGCAAGTGAGTGGCTGACAGTGAAGTGTAATTTGAGAATTACATAATTTTGG
ATTGTGTATCGGTGTTTAAAAGCTTCCCGAACGCAAAAGAAACAAGCGAAAATGGATGTTGCTGTGAAGCAAGAAATTAATGGGTCCCCGTTGCCGCAGCGTCCACAGCAGC
AACCGCATGGTGGTGGTGGTGGGGGTCCCGGCCCTATGCAAGGCAATAATCAAGCTGGGCCCGATCAGCACGGCCCAGGCGGCCCGGGCGGACCGGGTGGACCCGGCGGACC
AGGCGGCCCGGGTGGACCGGGTGGACCGGGAAACAAGAACATGAACAGAAATAATCGCAACAATAGCAAGAATCGCCAGAGCATGCCGAATCGCAATCGCGGTCCGGGTGGT
AATGCTGGTGGCGGCGGTGGCAATCCCAACAATAACGGTGGTCCCGGAAACAATCGTCCCGGCAACAATGGAGGAAACCCTCAACAAATGGGAGGAGAGGATTTCGCTTACT
TTATCAACGAGAAGCTTCGCCAGCTGGCCGGACCGCTGTTCGAAATTCCACCGATCGAGGCACAGGATGTGAAATTTTCTGGCCGCAATCGGCTCTACGTCGGCAACCTGAC
CAACGATGTGACGGAGGAAGAGCTGGTTGATATGTTCAAAGTGTACGGCGAGATCAGCGAAGTGTTCATGAACAAGGAGAAAAATTACGCCTTTGTGCGCGTAGATTATCTC
TCGAATGCTGAGGCGGCAAAGCGCGGCCTCGACGGTACCACGCGCAAGAACCGTGTGCTACGCGTGCGATTTGCCCCGAATGCAACAGCGATCCGCGTCAAAAATCTGACAC
CGTACGTCACGAACGAGCTGCTGTACAAAGCGTTTGAGGTGTTCGGTCCGCTCGAGCGAGCCGTCGTGCAGGTGGACGAGCGTGGTAAACCGACCGGCGAGGGCATTGTGGA
GTTTAAGAACAAGCCCGGCGCAATGGCCGCTATACGATACTGTACGGAGAAATGCTATTTCCTGACCGCGTCACTGCGGCCAGTCATCGTGGAGCCGTACACGTACCAGGAT
GACAACGATGGGCTGCCGGAAAAGTCGCTCAACAAGAAGCAAAACGATTTCTTGAAGGCACGCCAGCAAGGGCCGCGCTTTGCCGAGCACGGTTCGTTCGAGTTCGAGTATG
GCCAACGCTGGAAGCACATGCACGAACTGTTCAAGCAGAAGGCCGAATCGTTGAAGCGCGAGATGATCATGGAGGAGGAAAAGCTCGAAGCACAGATGGAATACGCTCGATA
CGAGCAGGAAACAGAGTTGCTGCGAGAGCAACTGCGCCAACGCGAAATGGACCGCGATCGTCAGAAGGCTGAGTGGGAAATGAAGGAGCGCCAGGTGCAGGAGGCCCGTCAA
CGCAATGACATTCAGATTAAAACGGAACTCGAGGAAATGACTACCCGTATCAAACGATCGGATGAAGAATTGCATCGCCGACAAAAAGAAAACAACATGTTCATGCAGCAGC
AAATGAACGATATGGGTGGTATGGGTAGTGGTGGTGATGGGGGTGGCGGCAGTGGCATCGATAACCGCCGTAACTTTGATATGATGAATCAAGTTGTCGGTGGTGGTGGTGG
CGGCGGCGGCGGCGGTGGTGGCGGCAATGGTAACTTCGGCAACATGTTTGAAAAAGAATACCTAAGCGAACTGCTAAGACTGCTCGGAACTGGATCAAGGGATAATGCGCAG

GGTCCAAATAACATCAACATGAAAGGAAAGACAGCGAATAAAAAGAAAACATTCAGAGGAGGACCTAGACCGAATAAATGAACTTTTTTCTTCAATTCAGTTGACACTGTAA
ATAGAGAAACTTTTTGGATCCTAAAATGCCTCTGCGTGTAATTTCAAAGATTTCCGAAGGATCACACAAGACATCCGGAAACAGTGGATCCACCAACTGAAAAGGATTTCCG
AAGGATCACACAAGACATCCGGAAACAGTGGATCCACCAACTGAAAAGGATTTCCGAAGGATTACACAAGACATCCGGAAACAGTGGATCCACCAACTGAAAAGGATTTCCG
AAGGATTACACAAGACATCCGGAAACAGTGGATCCACCAACTGAAAAGGATTTCCGAAGGATCACACAAGACATCCGGAAACAGTGGATCCACCAACTGAAAAGGTTATCTG
ATTTCATGAGTTTCAACTTAGGTTCTGGATGAATTTATCCGGGAAGATCTGTTTGTTGTGGACAATGGAACACGTGTTTGATACAGATTACTACCAAGTGCGTCAATTTATC
TTATCAGCGTCCGGATCTTATCTGCTTATGCTATATACAAAAATAAGCTATAGGCGTTGATAACAAGAAAAGAAAGACAACTTTATGAAATGTGATGTGGTATAAATGACCG
AAATCTACAAAAGTACAGTGCAGGTTTCCTCGATATCTCTTGGCGCTGATCATGCTTCCTGTATCATGGCGAAGCGAAGGAGATGTGTTGCATGTAAAATGAGCAGCTATCG
GAAAGGATTGGACACGACCTGGTGTGTAAAAAAAAACGCATTTCGTTTGTGGCATGGACTGGCCTTTTGTATGCTGGCAACAAACTGAGAAAGTACCGAAGTGAAGATACTG
CTCCTGAAAGCTGTATATGAATAAATTTCGGTTGTTTTAAAATAGTACACGTGTTTGTGACATCTAGACACAACGTTCAGGATGAGAAATAAAATTTATTTTTCGTAATTTG

>**AstHrp65 [LOC118502614_t1] [*Anopheles stephensi*]**
GGAAACTGCTTTCTTCATCCACTTTTCTTTGCTGCTCGTGGCGAACGGAAGCAAATTGGTGCTGGTCCCGCCGTGCATAGTTGACCAGTTGGTGAAAAGTTCTAAGTGCGCA
TAATTTTAAACCACGCATAGATTCATATTACATAACCTAGATCGGGAACTGAGTGCGAAAAAGTGTACGGCAAAGAAATAGTGTGAAAATGGATGTGGTAGTTAAGCAGGAA
TTGAATGGCTCCCCGTTGCCACAGCGTCCTCAGCAGCAGCAACAGCAGCATGGTGGTGGCGGTGGCGGCGGCGGCGGCACTCCGGGACCGATGCATGGTAACAACCAGTCCG
GTGGTCCCGATGGACCAGGAGGTCCAGGCAACAAGAACATGAATCGGAACAATCGCAACAATAGCAAAAATCGTCTTAGCATGCCGATGCGCAATCGTGGGCCTGGTGGTGT
TGGCGGTGTTGGTGGCGGCGGTGCTGGTGGAAACGGCAACAATGGAGGCCCGGGAAACAATCGGCCCGGCAATAATGGGGGAAATCCTCAGCAGATGGGCGGTGAAGACTTC
AGTCCGAATCAGCACCAGAACCGTGGAGGCATGAATCGTGGCGGCCACAACCGCAGTGGAAATGATATGGATAACAATTTCGGCGATCGTCGTCGCGGTGGCGGTGAGGCTT
ATTTCATCAACGAGAAGCTTCGCATGCTCGCCGGTCCTTTGCTCGATATCCCACCGATCGAGGCGCATGACACCAAATTTTCTGGCCGCAACCGGCTGTACGTCGGCAATTT
GACAACCGACGTGACAGAGGAAGAGCTGATTGAGATGTTCAAACCGTTTGGCGAAATTAACGAGGTGTTCATGAACAAGGAGAAAAACTACGCCTTCGTGCGTGTAGATTAT
CTCTCCAACGCTGAAGCTGCAAAGCGTAACCTGGACGGTACCACTCGCAAGAATCGTGTGCTGCGCGTACGATTCGCACCGAATGCGACCGCTATTCGGGTAAAAAATCTGA
CACCGTACGTGACTAACGAGCTGCTGTACAAAGCATTCGAAGTGTTCGGACCGCTGGAGCGAGCCGTCGTGCAGGTTGACGAGCGTGGCAAACCGACCGGCGAGGGTATTGT
GGAGTTCAAGAACAAACCAGGTGCCTTGGCGGCCATCCGGTGCTGCACGGAGAAATGCTTCTTCCTCACGTCGTCGCTGCGGCCAGTCATCGTGGAACCGTATACGCATCAG
GACGACAACGACGGTCTGCCAGAAAAGTCACTCAACAAGAAACAGGGCGAGTTTATGAAGGCACGCCAGCAGGGACCACGCTTCGCCGAGCAGGGATCGTTTGAGTTCGAGT
ATGGACAGCGCTGGAAGCACATGCACGAGTTGTTCAAGCAGAAGGCTGAATCACTGAAGCGCGAGATGATCATGGAGGAGGAAAAACTCGAAGCGCAGATGGAATACGCTCG
ATACGAGCAAGAAACCGAACAACTGCGCGAGCAACTGCGACAACGTGAAATGGATCGCGATCGCCAGAAGGCCGAATGGGAAATGAAGGAACGTCAGGCACAGGAAGCGCGC
CAGCGCAATGACATTCAGATGAAAACGGAGATCGAAGAAATGACCAATCGTATCAAGCGATCGGACGAAGAATTGCACCGCAGACAGAAGGAAAATACCATGTTCATGCAGA
ACCGTCAGATGATGGAACAGCAGCAAATGAACGATATGGGCATGGGAAGCGGTGGCGATGCCGGTGGTGGCATCGATAACCGCCGCAACTTCGATATGATGAACCAAGGTGG
CGGCAATGGTGGTAACTTTGGCAATATGGAACCGACCGTGCGAGACTATGATCACGGTCAAAACCGACCGACTCGGTTCGACGATGGACCGCAACCACAGCAGCAGCAGCAG
CAGCAGCAACAGCAACAACAGCAAATGCATCAGCAACAGCGCGGCAACAATTTTGGAGGCAATCGTAACAATTGGATGAACAATACACCTGACCGCCGATTGAACAATAACG
ATAATGATTTCCAAAACAAGCGTAGACGCTTCTAAAGAGGAGGATCATCATGATATGATGGGGGGTGAAAAATGATCGCTGAATTTTATTTTTCACACAGCACACAGCAAAA
CCTCATCAAATTGGTCTACGTTCAGAAAAGAAAAACAAAAGATGATGTTTACGAAACACCATTCGTATGCAATAATCACCCATCATCGCCCTTTTCTGTATTGCGCGACCCT
GCGCGTCATTGGTGGCAGGCAGCAATGAAGGGCTGGGAAACATATTTGACAAATTTTTGCATAACGACTCCCATTGCATCGTACTGTACACCTTTTGCCAACCAGCAATGGG
AATTTTAAAATGGTAGAAATTTACGATTTTTGGTGGGGGAGGGGTATTGAATGATGCTGCAGTACGTACCATTTTTGACTTGATCCTTTTTCGATTCGATTTTTTTTTACCA
CGATTAACATTCGATTGGCGTTCATTATAAAATTCATGTATCATTGCCTACACCCCAACAGATCTCATATCACATCAGATGGGATGGAAACAAAACGAAATTTTCATTTATA
TTCCTTGAACGTTCACAATGCATTATTTTAAGGGGGTGGGGTACACGCTGGGGGAAAATGTGTGGAAATGTAAGAAAACATGTTGTGTTGGGCAGCGACAAACAAATTAGTC
ATGTAAGCATAAATCTACGATCCCGTGGTTCGGCCTTCACGTGTCTGTCTCGAAGGAAAATCACCGTATGTGTGTGTATGTATGATAATTTTAAAACCCAACTTTAAACACA
GCATAACATTATTTAGTTAAGAAACGTGTCCATAAAAAGCGCCTAACCGTTGCTACAGAATACAAATACAAATACAAAACACACACATTAACCAACATCATTATGCATTCAT
AATATTTGCAGCAAAATTCAATGAACTGTTTTTTTGAATATGTGGATTAGAGATGAACGACGGCACGCGTTGTGCAAAATCTCTCTCAAATCTAAAATCTCTACCAGAGCAA
AGTATACAATTGAATTGAAAAAAACTAAGGTGTATAAGGTGTACATCATATGATTTTCTGATAAGCCCGCGCAATTGGGAGGGGCGGGGGGGGGGATGCCTTTCCGCATTGA
GTAGAACGGAAAAAGGCCTTCGAAACAATTTACGCAATTTAACTTAAAAGAGATGAACACTTATAGCTTAAAGAACTCAAGCATGAAGGTGATGAGGGAGTGACGACCACCC
ACCACCAGAGTGCAGAGAAAAGGAATAGTGTTTTAATGACATTTTATTTTTACACAAAAGTATTCTCTCCTTATAATTGTATGATGTTCTCGTTGACACACACACGATGAAA
ATTATGTTCCTCTTTGGTTTTTCCTGAACAAAAAAAAAAAATCCAATTTTACATTAACAAATGTCCGCCGTGCTTGTCTCGTTGTGTCACTACCACCGACCCCACGGTGTAAT
GTGTCCCTACAATATCATTTATGTGAATCGCTGATGGTGTGTGTGATGATTCAACGATTGAGATTTAGTTAAATTGCAACAGTACAGAATATATGCGTTCGATTTAATTTAA
CAGATTTATAAATAGAAAAACAACATATATGTATATTTCAAAATACAACCTTCTCGGGTAGAATAATATAAAAATTATCTCATAA

>**CquHrp65 [CQUJHB015709.R24371] [Culex quinquefasciatus]**
TTCTTCTTCTCATTCTGGTTTTAAGCGTTGACGGTGCGGCATCAGCTTTGTGTTCGCCATCGCAGCCCTTGATCGCTTCGGAAACAGGTTTGCAGCCGGTTAGTGTCCCCCC
CTCAAGCGTCGGAGAATCTGCTCTAGTGCCCAATCATGGAAGTGGAGCAACCTGTGGACAACGGAAGTCCGATGCCCCAGCGGCAGCAGAACCAAAACCAGGGCGGTGGTGG
CCCAGGGGGACGAGGTGGCGGCGGCGGCGGCGGCAAACAGTTTCAGAACAACCGGAACAGGAACAACCGTGGGGGCGGCGGGGGTGGAATGAATCGCGGAGGGGGCGGTGGC
CGGAACAGCTTCGGCGGTGGAAATCGTGGTGGTGGCGGCGGCGGTGGCGGTTTCCGGCAGAATCAGGATAATCAGGATGGTAATGGCGACGGAGATAACCAGCAGCAGAACC
AGGGGGGATTCCAGAACCGGGGCGGTGGCCGCGGCGGACGGAATCGATTCAGCGGCGGTGGCGGCCAGGGTGGTGATGGTGACCAGGGCTTCGACCGGCGCCGTAGCGGACC
CGGCGAGATGTACTTCATCGGCGAGAAGCTGCGCATGCTGCAGGGCCAGCTGCTGGACATTCCGCCGATCGACGAAACCGACGCCAAGTTCTCCGGCCGCAACCGGCTGTAC
GTGGGCAACCTGACCCCGGACGTGACCGAGGAGGAGCTGGTCGAGTTGTTCCTGCCGTATGGTGAGATCACCGAGGTCTTCATGAACATGGAGAAGAACTACGCGTTCGTGC
GCGTCGACTTCTTCTCGAACGCCGAGAAGGCCAAGCGCGAACTGGACGGCACCTCGCGCAAGAACCGCATCCTGAAGATCCGCTTCGCGCCGAACGCGACCGCCCTGCGCGT
CTCCAACCTGGGTCCGTTCGTCACGAACGAGCTGCTATACCGCGCGTTCGAGGTGTTTGGCCCGGTCGAGAGCGCCAAGGTGCAGGTCGACGAGCGCGGGAAATCCACCGGC
GAGGGCATCGTCGAGTACAAGAACAAGCCGAGCGCTTCGGCCGCGTTGAAGCACTGCAGCGAAAAGTGCTTCTTCCTGGACTCGTCGCTGCGTCCGTGCTTCGTCGAACCGT
ACACGTACCAGGACAACAACTCCGACGGCCTCTCGGAGAAGCTGATCAACAAAAAGATTCCGGAGTTCTTAAAGTCCCGCCAGCAGGGGCCGCGTTTCGCCGACCAGGGTTC
GTTCGAGCACGAGTACGGCCAGCGCTGGAAGCACATGCACGAGATGTACAAGCAGAAGGTCGAGGCACTAAAGCGCGACATGGTCATGGAGGAGGAGAAGCTCGAGGCGCAG
ATGGAGTTTGCCCGCCACGAGCACGAGATCGAGCAGCTGCGTGAACAACTCCGCATGCGGGAACAGGACAAGGACCGCAAAAAGGCCGAGTGGGAGATGAAGGAACGCTTCG
TCAACGAATCCCGCGAGCGACTTCAGATGCAAATGTCCGACCAGCGAATGGGCTTTCAGGGCAACAACAGCTTCAATAACCGTAACAATTCCTTCGATATGATGAACCAGGG
TGGCGGCGGCGGAAATCGCAACAACAGCTTCGGAGGGGAGGTGCGCGACTACGGCCACGGAGGACAAAAGAGACAGTCTCGCTTCGATAACCACGAAATGAACCAAATGAAC
CAGCAGCAGGATCAACAGCAAGGCCAGCAACAGCAGCAGCAACAGCCCCAACAAGGTCTGCCAGATCAGCCAGAACAGCAGGGACCGGGACAGGAACAGGGTCCGGGAGGTC
CTGCCGCCGAAGGTGGCATGGGAGACGCTGCTAACATCGTCGGAGGAAACGGCGGAGGCGTTGGAGCTGGCCCCGTTGGCATTGAGCAGCTGTTGAGCAACGCGTTTGGCGG
CGGCGGTGGCAACAACAATCGCAACAACAACCCGTGGAACAACGGCGGAGGCAACAACGGGGGAAACCGCGGCGACGACTTCCAGAACAAGCGAAGGCGCTTCTAAGCTCCT
CTGCAATCTCAATGCTCGAGTCCATTTTGCGCGCGTGAGTGTGTGTGAACAGACTGCAACACGGAGCGCATCAGCCGTACGCATTTGAAGCAGATCAAGTGACTATTTATTT
TCATCGTTTACGCGTACAAGTTCCACAGAAAACCACCCTACGCGATCGGCACCGCACACAACTCTCTCTGTGTGTATTCACTTCCTTCCAGACCACTTTTTTTTTTAGTTTTA
CTCGCGCGCTTGTAGGCTAAGAGGACGCATTGTTTTTTTAGTAGATTTAGACGCACGGCCTCCCTGTGTAAATTGTTTGGTTAGGACAAAAATGTGCATTCGCCACTCACAA
TTTTAAAAAGTAGTCTCAACCGGACTCGCGAGCGTACTGGTTTGTTGTGATGGAACTCGTTGCGTTCAATGCTTTATTTAAACGAATATACTTTTATTCGATAGGAGACAAA
CAAAAAAACAAAACAAATCTTATAGTTTATTAATCAGTCACGAGAGATGCAAAAAGATGCGCCATGCGTATTCGAATACGGAAATTAAATAACATTTGAACCCCAGATTAAA
CAAACAAAAAAAAAAAACTAACGGATAGAACATAAACATTGAGTGTAATACGCATTTGTGTCTGTGGTAGCGAAAGCAATGCATTAGGAGAAAGGAAAAAGACGCTCATCAA
CAACAACATAAAACAAAAAAAAACTGAGAATGTACACACCACCATATTGTGTACACACAAACACTTTTGTCTCCACCGGCACATGCATGCGAGTTTGAGAAAATAATGTACT
TTGAATCAACACAGTGTAACAATTATGACATAAATAGTATTCAATGAAAAAGTA

>**CquHrp65-1 [CQUJHB016553.R25562] [*Culex quinquefasciatus*]**
TCTCCTCATCGTCTCATTCTCTTGCCAACCTTCGAACGTGCCGGAAGTGCAAACCCAACCTGCTGCCTTTAAAATAGAACGGGACTTGAAGTGAAGAGTGGGGTAAAGAAAA
AAAAAAGGAAGAACAAGAGTGAATTGAGTGTGCCGCGTTTGGAACACGCCTCATCCGGGACCACCAGCGAGGACACCCGTTACGTAATCACCATGGAGGCCGCCCCAGCAGC

AGCAAAGCCGGAAGTGAACGGAAATCCGTTGCCACAGCGCCAGCAAAAGAACCAAAATCAGCAAAAGCAGGGTGGCGCAGCAGGAGGCGCTGGTGGTGGTGACGGTGGCAAT
CAGGGCCAGCAAAATCAGCAGCAGCAGCAGCAGGGAGGAGGAAACAAGAATCAGAACCAGAATAACCGCAATAGAAACCGAGGCGGTGGCGCAAACGGTCCGAACCGTGGTG
GTGGTGGTGGCCGGAACAATTTCGGTAACCGCAACAACCAGCAAGGCGGTGGAGGACAGGATGCCGGAAACGGCGAGGGTGGAAATAACCAGCAGCAGCAGCAGAATAACCA
GGGATTCCAGAACCGGAACCAGGGCGGTCCCAAGAACCGCGGTGGACCCCGGAATCAGGGCGGCGATGGCGAGGGTGGCGGTGGCTTCGACCGGCGTCGCGCCAACAACGAG
CAATACTTTATCGGCGAGAAGCTGCGCCTGCTGCAGGGACCGCTGCTGGACCTGCCGGCGATCGAGTCCACTGAGGAGGTCAAATTCTCCGGCCGCAACCGGCTGTACATTG
GCAACCTGACGGCCGACGTGACCGAGGAGGAGCTGGTCGAACTGTTCATGCCGTACGGCGAGATTACCGAGATCTTCATGAACAAGGAGAAGCTGTACGCGTTCGTGCGCGT
CGACTTCTTCTCGAACGCCGAGAAGGCCAAGCGCGAACTGGACGGGACCTCGCGCAAGAACCGAATTCTGCGGGTTCGATTTGCGCCCAACGCGACCGCCATCAAGGTCAAG
AACCTAACGCAGGACGTCAGCAACGAACTGCTGTACAAGGCGTTCGAGGTGTTTGGCCCGCTGGAGCGTGCCGTCGTGCAGGTCGACGAGCGCGGCAAGTCCACCGGCGAGG
GTATCGTCGAGTACAAGAACAAGCCGAGCGCGATGGCCGCCCAGCGGTACTGTTCGGAGCGTTGCTACTTCTTGAACTCGTCGCTGCGTCCGTGCATCGTGGAACCGTACAT
GTACCAGGACAACAACGCCGACGGTCTGCCCGAAAAGTCGCTTAACAAGAAGATTCCAGAGTTCATGAAGCTGCGACAGCAAGGACCGCACTTTGCCGACCAGGGTTCGTTC
GAGCACGAGTACGGCCAGCGCTGGAAGCAGATGCACGATCTGTTCAAGCAAAAGTCCGACGCGCTCAAGCGCGAGATGGAAATGGAGGAGGAGAAGCTGGAGGCGCAGATGG
AGTACGCCCGCTACGAGCACGAGACCGAGCAGCTGCGAGAACAGCTCCGTATGCGCGAGCAGGACAGCGACCGCCAAAAGGCCGAGTGGGAGATGAAGGAGCGCATGGCGGT
GGAGAACAAGCAGCGCAACGAGATCCAGATGAAGCACAAGGTCGAGGAGATGCAGAACCTGATCAAGCGCACCGACGAGGAACTGCACCGGCGCCAGCTCGAGAACAACGTG
TTCATGCAGAACCAGCAGATGAACCTCCTGGACGGCATGGATGACAACCGCCAGGGCTTCGGTAACATGCGAGGTGGTAACAACTTTGACGCCGGAATGGAAATTCGTGATT
ATGACCACGGTCAGAACAATAGACAGTCACGCTTCGACGACGGCGGCATGCAGCAGCAGCAAGGAGGTGGCGGAGTGAACCAACGTTTGGGCAATTCATTCAATGCCGGTGG
AAACAATCGCAACTGGATGAGCAACAACCGCGGAGGTGGTGGCGGCGGCGGCGGTGCCAACAATGACGATTTCCAGAGCAAGCGAAGACGCTTCTAAGCTTGGAATCTCGCA
CAGATACGCCACAAACTCTGTGTTTCGCACGACGTCCACGTGGCAGGGCAAGCGCGTGTTGACCTGATTATGTTTGAGCCGCTGTTCGCCCTCCACCTTCTATTGGTCTCGC
CGTTACGTGGTCCCACTGGAGGGAACGGATTACGTGCTTTTTTTTTTCTTCTTGACAACAAACATTCTGGCGGAAGCCGACATCTCACCCACGGTACAATGAAGTCAATTGT
ATCGATCTACGGAGGGTCTCCCCCAGAATCATTGTGACCCAACTTTTGCATACATACAGTTTAAGGACATTATTTTACACGTTGTGAGATTCAACACCCTTTTTAAAAAGAC
AGTTTCGTGAAAAACCAACGGTACAAAGCTATTTTCTACGAGAGGGCGGGCTTCATTTCGAAACGTTTGAAAAGTGGATTCTTGGGCACCATTTGCCGCTACACAACTGTTG
CAAACACATTTAAAAAGAGTGCGCGACCACCTGCTGGAGCAGCTGTCTGGCACGGGTCACATTTTAGCAAGCAGCAAACACACATCCAACACACAAATCACCGTAACAATTG
AATAAAAATAAAACGTAACTTATTATGTCACTTGATATGTGAAACAAATAGAGATAACTGCTACGGCAGCAACAGTAATTATCATATTCTTAAACAAAAACAAAAAATCTTT
GCAGCTGAAAAGCGTCAGCAAAACACATTAAAAAAATCATTGTAAAAAGCACCATGCAAAATGCCACTCTGTGGGCAGGAAAGTGTCATTCGCCGCAGCGAATAATTTGCGT
TGTATTAACAAACAAATCACACACATTTTTTTCTGGCTAGTGCGCTCCACAGCAGCACATCGACGGAAAGAAACAGTACTATTTATAAGGCAAACAAAAACATTATTTTTAA
ATCTAGATTGAACCCTGTACGTGTAAGTTTAAAGAAAAAAAAAAATACCTTGAAAAGCACTGTGTAGTAATAATATAAAGCAATATTCATTGCGAATAATAACTAA

>**AalbHrp65 [AALB003148-RA] [*Aedes albopictus*]**
TTTCCCTCTGGTCACCTTCGCGAACGGACGTGGTTCTTGGTGACGGTGACGCGCTCGGTTAGAAGGGAAAGAAAAAGTTGGGATTCTGTAGAATTTTATCGGACATTCGCTT
GTGCGTGCGTATGCGGGCTCTTATATAAGCAGCAACGTGTTTCTGTCTAAAGAAAGTGCAGTGTCGTGTGAATAAGCCCATCGAAGACCCGGACAAAACCGTGCATCATGAT
GAATCCGGTGGTGAAGCAAGAAATTAATGGTTCCCCCTTGCCACAGCGTATCCCTCAGCAGCAGCAGCATGTTGGTGGTGATGGTGGTGGTGGCGGAGGGGTCCCGGGAGGT
AATCAGCCCGGAGGCGGAGGCAACACGAGTGGCAATGATCAACACGGTCCCGGTGGTCCCGGAGGGAAAGGCAACATGAATCGCAATAACCGAAATAACAGCAAAAATCGGC
TGAGTATGCCTAACCGTAATCGTGGTCCGGGTGGTATGGGAATGGGTGGTGGCGGCGGTGGTGGGGGCAATATGGGCCCCGGAAATAACCGTTCAGGAAATAATGGGGGCAA
CAACATGCAGCAATCGCCACAGCAGGGCGGTGGCGATGATTCTGGCCCAAATCCGCACATGAACCGTGGCATGCATCGTGGAAATCGCGGCGGAAACGATATGGATAACAGC
TTCGTTGATCGTCGTCGTGGCGGCGGAGGGGACGGATATTTTATCAACGAGAAGCTTCGTTTGCTGCAGGGACCGCTGCTCGACATTCCGCCGATTGAGGCACAGGAAGCCA
AGTTTTCCGGCCGTAATCGCCTGTACGTGGGTAACCTTACGAATGATGCGACGGAAGAGGAGCTGATGGAGATGTTCAAGCCGTTCGGCGAAATCAGCGAGGTTTTCATGAA
CAAAGAGAAGAACTACGCCTTTGTGCGTGTAGATTACTTCTCCAATGCCGAAAAGGCGAAACGGGAGCTGGACGGTACTTCGCGCAAGAACCGTGTATTGCGTGTTCGCTTC
GCTCCGAATGCGACCGCCATTCGCGTCAAGAATCTGACCGCATACGTAACGAACGAGTTGCTGTACAAGGCGTTTGAGGTTTTCGGGCCACTCGAGCGGGCAGTGGTGCAGG
TAGACGAGCGTGGTAAACCCACCGGCGAGGGAATTGTCGAGTTTCAGAAAAAACCGGGCGCAGCAGCTGCGATCCGGTACTGCACTGAGAAGTGCTATTTCCTCACTTCCTC
CCTACGCCCCGTTATCGTCGAACCGTACACCTATCAGGATGATAACGATGGTCTGCCCGAGAAGTCACTCAACAAGAAGCAGAATGAGTTCATCAAAGCTCGCCAGCTTGGG
CCACGGTTCGCGGAGAACGGCTCGTTCGAGTACGAGTATGGTCAGCGCTGGAAGCACATGCATGACATGTACCGTCAGAAGGCCGAAGCCCTAAAGCGTGAGATGATCATGG
AGGAGGAACAGCTCGAGGCGAAGATGGAGTTCGCTCGCTACGAACACGAAACGGAACAGCTGCGTGAACAACTTCGCCAGCGCGAGATGGATCGTGACCGCCAGAAGGCTGA
GCTCGAAATGAAGGAGCGCCAAGTGCACGTGGCGCGTCAACGTAACGACATGCAGGTCAAGACGGAAATGGACGAGATGACCAACAGAATCAAGCGTTCGGATGAAGAGTTG
GACCGCAGGCAGAAGGAAAATAATATGTTCATGAAGAACCAGCAGATGAACTTCATGGACCAGCAGCAGCAAATGAACGACATGGGTGGAATGGGTGGTGGTGGCGGAGGCG
GCGGCGGTGGTGGTGGTAATGACAATCGTCGCAACTTCGACATGAATCAAGGACCTGGCAATGGCAACTTCATGAACAGCTAA

>**DmelnonA-l [*Drosophila melanogaster*]**
GCAGTTGGAATAAACTCCTTTCTGTGCCTGGTAACATTTTTTTTCATACCTGACAGTTCCTCCAATCGGTGTTTTTTTTTTCGCATAGAAAATTGAATTTGGGAAAAAGTATT
TTTCGTTTGCCATGGAGGGCGCTGTTAAGAAAAACAGTTTGAACTCTTCGCCATTGCCACAACGCCAGCAGAGGGGCAATTCGACCAATAAGAACCTGGGCAAGCCAACGCC
CCCGAAGCTAAATGCTGCCAGCGACGGTAATCCTGCCGAGAAAAAGGCAAGACTTGGAGGAAACACCCAGAACGGTGGTGGAGTAGCTGGTGGCGGCGGCACTGGCGGTGGT
GGTGGTGGTGGTGGTGCTACTGGCGGCGTTGAATTTTCCAGAAATCGCAATCGAGGCGGCAACCAAAATGAAAACCGGCAGGGTTTCCAAGTCGCAAATAATTCACATCAAA
AGCAGATTAACGAATCGCCGAAGCCGGCTGCGGGCAATGTGCCCGCAAAGAACAATGAACTCTCAGCTGGCGGAGGTGGTCAAAACCAGCCGAACCATAGTAACAAGGGCCA
GGGTAACCAAGGCGATCAAGGAGAACAGGGAAACCAAGGACCGAATTTCAGAGGTCGTGGTGGTGGCCCAAATCAGCCGAATCAGAATGCCAACCAGGAGCAAAGCAATGGA
TATCCCGGCAACCAAGGAGACAATAAAGGAGGACAGGGCCAACGCGGTGCTGGAGGTGGAAAACACCAGCGTGGAAACCGCTCTAGGCGCTCTGGGGGATCTGGAATCATGA
ACTCATCAATGGGCGGCGGTGGGCAGCGCGGCGAAGACTTTTTCATTGCCCAACGACTGCTCGACATTAGTGGACCCACCCATGAGTTGCCGCCCATTGAGTTACCCACCGA
TAACAAATTCGTCGGACGCAACCGCTTATATGTTGGCAACCTCACCAGCGACACAACTGATGACGACCTACGGGAGATGTTCAAGCCATATGGCGAGATCGGCGATATATTC
TCGAACCCGGAGAAGAACTTTACATTCCTGAGGCTAGACTACTACCAAAATGCTGAGAAGGCCAAACGCGCTTTAGATGGCTCCTTGCGCAAGGGACGAGTGCTGCGTGTCC
GCTTTGCGCCCAACGCCATTGTGCGTGTGACTAATCTCAACCAGTTCGTGTCCAACGAGCTGCTGCACCAGTCCTTTGAGATCTTTGGACCCATCGAGCGCGCCGTTATCTG
CGTAGACGATCGCGGTAAGCATACCGGCGAAGGCATTGTTGAGTTCGCCAAGAAGTCCTCGGCCAGCGCCTGTCTGCGCCTGTGCAACGAAAAATGCTTCTTCTTGACTGCT
TCATTGCGTCCGTGTCTGGTGGAACCGATGGAGGTGAACAACGACAATGACGGCTTGCCGGATAAGACGTTAAACAAAAAGTCGCTGGAATTCAGACATGAGCGTAGTGTCG
GACCGCGTTTCGCTTGTTTAAATTCGTTTGAGCACGAGTATGGATCACGCTGGAAGCAATTGCACGATCTCTTCAAGAGCAAACAGGACTCACTCAAGCGTGAGTTGAAAAT
GGAGGAGGACAAGCTGGAGGCCCAGATGGAGTACGCTCGCTACGAACAGGAGACCGAACTGCTGCGCCAGGAGCTGAAGAAGCGCGAGCTGGACAACGAGCGCATGAAACTG
GAGTGGGAGATGCGTGAGAAACAAGCCGAGGAGATTCGAAAGCGGGAAGAGGAATACATGCATCGTTATCAAAACCAATTACTTCGCCATGAGGAGGATATGCGAGCCCGTC
AGCAGGAGAACGATTTGCTAATGCAGGCCAAGAAACTCAACATGATCCTCGATCAGCAGGAGGGATTCGGAGGCAGCAACTCTGGATTCGAACACTTTGATTCCCCTTTTGA
AGTATTTGGGAACAACAGCAACAATTCAACAATGGCTGGACCTGGAGGTCCAGATAATAGCGACGGAAACCAACACGGACACATTGATTCATGGGGACACCGACGCTTCTAG
ACACTGAAACCTATATAGATAGCGACGAAAACAATAGTGATCTCATTAGTTACGCATAATATCGGACATTTCGCTCGGCTATGCGTGAACAATAACATAAATAACAAATGGG
ATCATGCAAGCGAAGATGCATTCATCTCATTATAATATAAATTGAATTTATATAATTAACCAAAAACTGTTAAAAACGGTCTCAACAACAAATCCACCACGAAATATTAACA
CAAAAATGACAAAATCCAAACACGAATGGCAACTTACAAGATGTGCAGTATTTCGAAGAAAAACACCTGAACATAACCATCCATCATCCATTAGATATATGTAGAGACTACC
TCCTAACACGATTAATTTTTGTCTATCATTCAAATTAAATATTGAATGCGACTGAAATTACTGATTATTTGGAGTCGAATATAGATAAAGAGCCTAATTTTTATAATCGTAG
ATTATACTTTGATTTATGAGTAAATAATCGAAATCGGACTTGCTTTTATTTCAATGTATTTCTCATCCCATCTCTGTCTACTTTTAATAAAAACGGCTCTAACCTAAGGTTT
CACTGGGAATTTATGTTGGTAATAAATGTGTTAAAGCGGAAAAGTCGGAAAATGCCATCAGCTCTATTGGGAAATCCGGCAATAAGTTGCAAGGCTGTCCGAAAGGACACAT
TTCCGCCGGTATCTTCAGTCCCACGACGCTGTCCATATATTAGGTCAAAATGCAATCACTTGTTTAAACAAAAGAATTTATTTGATCAACAGAATGAACTATTGTATGAACC
CTCCTCCCATTCACCCAACAATTACACAATTGTATTGTACCGAAGCAATCAAAGATTTCAGTTCAATTACCCGTGGTATAACCATCAATTTGAATTTCCTCGAGATGG

>**DmelnonA [*Drosophila melanogaster*]**
GCTGTGTACGTATAATACGTTTATCTGCTTTGTTCCATTACATTCAGTTTTTCGATTTTGACGACGGTTGTCAACGAGTTGTAGCAAGCACATTTTAATAAAGCGAAAGGAA
ATAATAATATTTAAAAAATGGAAAGTGCGGGGAAACAAGATAATAACGCCACGCAACAGTTGCCGCAGCGACAGCAGCGGGGAAACCAGCAGGCCAACAAAAATCTCGGCAA

GCATAATGCACAAAAACAAAATGATTCCGCGGACGGAGGCCCGGCTGAAAAGAAGCAACGATTCGGTGGTCCAAATGCCCAGAATCAAAACCAGAACCAGAATCAAAATGGT
GGTGTAACGGGCGGTGGTGGTGCAGTTGGTGGCCCCAATCAAAATAAGAATTTTGGAAACAACAAGGGTGGGTTCGTTGGAAACCGCAATCGCAACAATAATCGCGCTGGAA
ACCAAAACCGGACTTTTCCAGGCAACAACAATCCAAATCAAAAGCCCAATAACGAAACATCAAAGGCGGACGGCCCTAATGCACTTGCTAAGAACAATGAACCGGCAACTGC
AGCTGCTGGCCAAAATCAAGCGAATCAGAACGCCAACAAGGGCCAAAATCAACGGCAAGGACAAAACCAAAATCAAAATCAAGTCCATGGTCAAGGAAATCAAGGAGGACCA
GGAAATCAAGGGGGAGCAGGCAATCAAGGAGGCCAAGGAAATCAAGGAGGAGCCGGAAATCAGGGAAATGGTCAAGGATTCAGAGGTCGCAATGCTGGTAACAATCAAGGTG
GCGGTTTCTCCGGAGGTCCGCAGAACCAGCAGCGTGACAACCGAAACCGGAGTGGTCCACGCCCTGGCGGAGGTGCTGGTGGCGCGATGAACAGCACCAATATGGGTGGTGG
TGGTGGTGGCGGTGGAGGCGGTGGTCCGCGCGGCGGTGAAGACTTCTTCATTACCCAGCGGTTACGCAGCATTTCTGGGCCCACCTTTGAACTGGAACCGGTGGAAGTGCCC
ACGGAGACGAAGTTCTCTGGACGAAATCGTCTCTATGTGGGCAACCTGACCAATGACATCACCGACGATGAGCTGCGCGAGATGTTCAAGCCATATGGCGAGATTAGCGAGA
TCTTCTCGAACCTGGATAAGAACTTTACATTCCTAAAGGTCGACTATCATCCCAATGCCGAGAAGGCTAAACGCGCCCTGGACGGATCGATGCGAAAGGGTCGCCAGTTGCG
TGTACGATTCGCACCGAATGCCACCATTTTGCGGGTGAGCAATCTCACACCGTTCGTTTCCAACGAGCTGCTGTACAAGTCTTTCGAAATCTTTGGTCCCATCGAGAGGGCC
AGCATCACCGTCGACGATCGTGGCAAGCATATGGGCGAGGGCATAGTTGAGTTTGCCAAAAAGTCATCGGCCAGCGCCTGTCTGCGGATGTGCAATGAGAAGTGCTTCTTCC
TGACTGCTTCCCTTCGACCGTGTCTGGTTGATCCGATGGAGGTGAATGACGATACCGACGGACTGCCGGAGAAGGCGTTCAACAAAAGATGCCCGACTTCAACCAGGAGCG
TAGCATTGGTCCGCGCTTCGCCGATCCCAATTCGTTTGAGCACGAGTACGGTTCGCGCTGGAAGCAGCTGCACAATTTGTTCAAAACCAAGCAGGACGCGCTCAAACGCGAA
CTGAAAATGGAGGAGGACAAGTTGGAGGCTCAGATGGAGTATGCACGCTATGAGCAAGAAACTGAACTGTTGCGTCAAGAGCTGCGAAAACGCGAAGTTGACAACGAGCGCA
AGAAATTGGAGTGGGAGATGCGCGAGAAGCAGGCCGAGGAGATGCGCAAGCGCGAGGAGGAGACCATGCGACGTCATCAGACCGAGATGCAGAGCCACATGAATCGCCAGGA
GGAGGACATGCTCCGTCGGCAGCAGGAGACACTGTTCATGAAGGCACAGCAGCTCAATTCGTTGCTGGATCAGCAGGAGGGATTTGGTGGCGGCGGCGGCGGTAACAACTCC
ACTTTTGACAACTTCGCTGGCAATAGCAATTCGCCATTCGAGGTTTTTAGAGGCAATAACAATAACAATTCCACAATGATTGGAAACAATGCTGCCCCCAACACACAGGAGA
TCAAACGCCTTGACGGTCCTATAAACGAGATTACTTCAAACTTCAGCCCACCAGATTTGGATTCGGATATAATTTTAGTGGCATTCGTATTGGAAGAGTTCTCATCTCACGT
GTTTTATACGAAAATTGTACAGAAATGTGAAAGCTTTAAGAAACGCACACAGCTTAATGCTCGATCAAATATTATTAAAAAATGCATAAGCAACTTAAATTGGTTAATTGTT
TTATGA

Supplementary Text2. *Asudsx* and *Asufru* sequences.

>***Asudsx*F1**
ATCGCCGATGCCAATGTTCGATCAGAATTGAAGTTTTGCAAATACAAATGATAACAATCATTGATGAGTGTGAATAACTCCATTTCCCAGTGAAGCCTACACCGCAACAGGC
TGCGGTTTCGATCTTAACCGAGTGAACTGAGCTGATAAACGCGATAGCGGCCGGACATCGTTTGATTTTTCGTTGCTGTGTGCTGAAGAAAAAGCGAATATTTTAGTTTTGT
TTTTGCCCTGCTCGTTCGTACACTCGCGGGAAGCCGGTCGACACGCGGTATTTCAATAATCTCGTTTCGCTTCATTACGAATGCCAACGCCGCGCAAACGTTAGGCAAGGGC
AGTGAGTCGCGTCCGCGAAGAAAGCGCGAGGAGAAAAGCGTGAAGCATACCCCGATCGGAGTGGAGGAGCAACATTCGCAAAATTCAAAATAGCCGGCGATCGGAGGTAACA
CAGAAGTAAAAGAAGCAAATCAAAAATCCAGAAGATAATAAAACAGTGAAGCAACGGTGCTAGTTGAAAATCTTTCCAGAACAAGCGATCGGACATACCTACGGCTCGGTTA
TTCAAGTGCATGTGAGAGATTGTTTGATGATTTATTAGCGTTGCAGTATCATACTGATAGCGGCGTTGATTTGGTGCAGAAAAGGAAGATACTGCGTCTCTCCCAACGATAT
CCGATGAATTTACGGTGTTCCAAAAAATAACAACAAATGTGTTTAAATTAATTAAAATACAGTGAGAAGGGTGACTTCCTAAAAATGAAATACTTTAAAACCTAAAAATGTT
CGAAGCGGTTCTAATCACCACGCCACACTAAAGAACGGCTGTAGAACGGATTTTCTAATTCATCGTGTTTCTGGAAAACCTCACGCAACGGAACAACACTTGTTAGCAATCG
ATTCAGACCGTCGTTTTCGGTTTCCATAAAGAGTATGTAGTGTGATTCCGGGAAATCAGATCCGACGGAAGAACATTGATCACAATCGATTGGCGCCATCGGGACGAGTATG
TTGTGGCAAAATTATTGTCCATTCGAAGGCGTCAATTCGGATGGGTCTTTGCGATCTCGTGGGCTCGATCGAGATTTTAGCTTCATGGAAGTACTTCCCATAGTAAATGTTT
TCTGAATTACGCGTCGCCTTTTGAGGGTGCGTCCTGGTACATTGTCCCCCTTCTGACCAGGATCCCGTCAGTATCGAGCAATACAGTTTTATTTTCTCTCTGTAGTAGTAGT
CATAGTAGTTACATTTGTGTGCAATATTGCGTGAGTTAGCGCGAACAGTGCAGTGATAGAGTGCACTTTCGGACCATTGCAGCTCGATAACTAGTTGAATTGAAGGAATTGT
AAGAGAGGGAAAGATCTTTTCTAGCAAGATATTGCTAAATCGGTTGATGGTTTCGCGAGAACGCTGGATAGTAAAGATGTCCGAATCAGGTTACGAGAGCCGAACTGATAAC
AACGGAGCGTCCACCAGCATGAACCCGCGCACACCGCCCAACTGCGCCCGCTGCCGCAACCATGGTCTCAAGATAGCCCTGAAGGGACACAAGCGCTATTGTAGCTTTCGAG
ACTGCAACTGCAAGAGGTGCTGCCTGACGGTCGAGCGGCAGCGGATCATGGCCAAGCAGACGGCTGACCGACGGGCGCAGGTCATGGACGAACAGCGGCTGCTGCAGGACGG
CGAAGTGCCCCCAAAACCGGAACATAACCTTCTTGTGCCGAAATTGTGCGACCTAAAAGAAATTAGCCACAGTTCGCATCCGAGAACGAACGAGTGCGATTCGTTCTCCGGC
TCGATGAACTCCAACCCGGACAGTGCGTCGGTGGCTCTGGCCCTGCACCGAAGGTCATCACCTGGCAGAGCAACGCTTCATCCACAGCAGCACGCTTCAGCCAACCATCCCA
GCGTATCACCTGAGCCCGCAAACAGCATGTTATCAGCCTCATCAGCGCGAGTCGGTCCAGAATCGGGAACAGATGACGAACTAGTGAAACGATCTCAGTGGCTTCTGGAGAA
GCTGCACTACCCGTGGGAGATGATGCCCCTAATGTATGTGATATTGAAAGGCGCCGACGGGGATGTCCATAAAGCGCACCAAAGGATCGATGAAGGTCAAGCCGTGGTCAAT
GAATACTCACGATTGCACAATCTGAACATGTTTGACGGTGTGGAATTGCGCAGTACGACGCGTCAATCCGGATGATAGACTTTTTACGCGAACAGCTGTTTAACCGCGAAAG
TTGTACCCAAACAAGAACAAACATCGTGAATTCTATAAGTCTTAAAGAAACCGGATGCAACAGAAGAAAACGACGATATCGCAAATATACAAAGCGTAACAGAAAACCCGCA
AGTTCAAACTAGTGATTACAATTAAAATATTTTAAAAAGATAGTGAAACATAGATTTTTGAAGCTAGTTTACAAAGTGCTCTACACCTAGCAAAGAGATCTAATACTAGATC
GTGGGTATTCATTTGAACCCGTCGCGTGAAACGGAAAGTGATCGAAATTAGGCCAAATGTGCGAAAAACTGTGAACACAAATGCCTTTTGGGCTGGAGAAGTTGAGTGCAAA
TGCTGTTCAACGATAATAGCGAACTTCAGCCATTCTGGGGCTACCACGTGTAGCTCTACTTGTGAGACAGCGATCCTAAAGTGTGTGAAAGTGCAAACCAGTGAAGAAACCC
ATAGTGCAAAGCAGGTTTTAGATAAACAAGTGATACTGCAAACGGTTTTACTACTTATTAGTTAAAGTGTTTTTCGAAAGCCGAAGTGTGTTCCACCCGCCCAGTGAAGAGA
ACTTCTGCCGACACTAAGATCACCATAAAAAAGCAACACCCAACTGGGCCAACCGCAGTGGTTCTTCCACCGACTATCATCAATCCCAACTGGGCAACCGCAGTGGTTCTGC
GAACCAACTGCCACCAATCATCAACAAATCTTTTCCAATCGTCTCGAAAATCGTTCGATTCGATTGCAACCTAAACACTGACAATTTGTCTCTCTGTCACTCGCATGCCTCG
CCTGTGCATCGTTTGATGCCATATGGCCAAGTGACTGCTAAAGCCAGCGAAAAACATGTTGGCCCAGAACGAGAAAACGAGCAGTCTCTTCTGGTCACGGTCACGGTCACGC
TCTCATTCGCCGCAAAATGACGAGGGCGTTCTCAACCTGGACATGAAATCTACCAAAGTATATGCCGCTAACGATGAAGAAGCTGCACTCGATGAATCCACCCCGCGATCTC
ATGATTCCGCCGAGTTACGCTCCCGCGTACAGGTTTCCTACTACGGTAGCATAGAGCACGGTGCGAGCAGTAGCAGTTCTCATCGGAATGATGACAAGTATAGCTCGTCGGA
GGACATCCAAGTGCAGTCAGATCCGGAAGACAGCGACAAAGTGCAACTGCATCACGATGCACTTAGTAAAAGTCGGAAAGCGGCGCACAAGGATGTTGATTATTACGCAATT
AAGGATAAACCCTTTCCTCCGCTATCCAACGGATTTGAGTCAGCCGAACTGTTCAGGAACGGTCTTCACGTTGCCGGGTTGAACACGATCAATTTCACGGCAGACCTCCACC
AAAAAGCTGCTGCTGCTAACTTGATACAATACTTTCCCGTTGCGCCATGTCCCACGTTTGCACAGTTCGGGCTGTACCCACCGAATCTGGTGTATTCTCAACTGCATGGCCT
AAGTTACCAGAAGGAAGTGCTCAAGGATCACCGACCATCTTCTCCGTCCCGTTCGTCACGAACCTCGCTGGTTGACCCAGCACTGATAGATCCCAGCCATCCGCATCAGTCG
TCTGCAGCCGTAGAATCGGTCCACTAGTACGAGCTGCCACATTCGGTCGAGTGGCTCCACGTCAACGGTGCTGCCTTCTAGTTCTTGGACTGGTTGGGGAACTGCTGACTAT
CTATCCTGAGAATTATGAATCCATGCAAGCTTAACCGAAAGGCCGAAAGCTTGCATCGTGCATCTGATAAAGGTTTCGTGACGTCAGAGACGCTTGGCAGCCGCTCCCATCT
GTGTAGAAAATGTATGTTAATCATGAGTTTTAAACTTTACACATTATGATGTATTGCTTTCTCTCACTTTCCGAGACAATTGTTTATCTATTGATTAGATAAAAAAAATGTG
GTGTAAGTATTCTGGAAAATTGTTGGCTATGTGTAAGGTAAACATTGAAATCAAGCAAAAACTGTAACTTACAGCCAGCAGAAAGCGAGGAAAGGAGTGGCTCAAATTAAAT
TTGAAATCATTCACTTCAAGACTATTATGACCAAATGAATCAAAGCTGCGAAAACTACAAGTTTTCATTGTGATAACGACAACACAAGAACAAAAAGATAACGCTGGAAAAT
TAGGACTTAATAAGGATCGTTGAGGCTTACGACAATCGAAAATGAACTTCATTTATCTGATTTTTGTTATTTGTCTTGTAACAATTAATTTTCATTCTCAAATTTGAACGAT
TTTATTGTAAAAACTGAAATGAAATTTATACTAAAAGATAACTGATTTGAACTATTATTTGTTAACAGTTAACAGTCTATTAAGCAGGCATGGAGAATTGGCGTTTATAACT
AAAGGAAGGTTG

>***Asudsx*F2**
ATCGCCGATGCCAATGTTCGATCAGAATTGAAGTTTTGCAAATACAAATGATAACAATCATTGATGAGTGTGAATAACTCCATTTCCCAGTGAAGCCTACACCGCAACAGGC
TGCGGTTTCGATCTTAACCGAGTGAACTGAGCTGATAAACGCGATAGCGGCCGGACATCGTTTGATTTTTCGTTGCTGTGTGCTGAAGAAAAAGCGAATATTTTAGTTTTGT
TTTTGCCCTGCTCGTTCGTACACTCGCGGGAAGCCGGTCGACACGCGGTATTTCAATAATCTCGTTTCGCTTCATTACGAATGCCAACGCCGCGCAAACGTTAGGCAAGGGC
AGTGAGTCGCGTCCGCGAAGAAAGCGCGAGGAGAAAAGCGTGAAGCATACCCCGATCGGAGTGGAGGAGCAACATTCGCAAAATTCAAAATAGCCGGCGATCGGAGGTAACA
CAGAAGTAAAAGAAGCAAATCAAAAATCCAGAAGATAATAAAACAGTGAAGCAACGGTGCTAGTTGAAAATCTTTCCAGAACAAGCGATCGGACATACCTACGGCTCGGTTA
TTCAAGTGCATGTGAGAGATTGTTTGATGATTTATTAGCGTTGCAGTATCATACTGATAGCGGCGTTGATTTGGTGCAGAAAAGGAAGATACTGCGTCTCTCCCAACGATAT

CCGATGAATTTACGGTGTTCCAAAAAAATAACAACAAATGTGTTTAAATTAATTAAAATACAGTGAGAAGGGTGACTTCCTAAAAATGAAATACTTTAAAACCTAAAAATGTT
CGAAGCGGTTCTAATCACCACGCCACACTAAAGAACGGCTGTAGAACGGATTTTCTAATTCATCGTGTTTCTGGAAAACCTCACGCAACGGAACAACACTTGTTAGCAATCG
ATTCAGACCGTCGTTTTCGGTTTCCATAAAGAGTATGTAGTGTGATTCCGGGAAATCAGATCCGACGGAAGAACATTGATCACAATCGATTGGCGCCATCGGGACGAGTATG
TTGTGGCAAAATTATTGTCCATTCGAAGGCGTCAATTCGGATGGGTCTTTGCGATCTCGTGGGCTCGATCGAGATTTTAGCTTCATGGAAGTACTTCCCATAGTAAATGTTT
TCTGAATTACGCGTCGCCTTTTGAGGGTGCGTCCTGGTACATTGTCCCCCTTCTGACCAGGATCCCGTCAGTATCGAGCAATACAGTTTTATTTTCTCTCTGTAGTAGTAGT
CATAGTAGTTACATTTGTGTGCAATATTGCGTGAGTTAGCGCGAACAGTGCAGTGATAGAGTGCACTTTCGGACCATTGCAGCTCGATAACTAGTTGAATTGAAGGAATTGT
AAGAGAGGGAAAGATCTTTTCTAGCAAGATATTGCTAAATCGGTTGATGGTTTCGCGAGAACGCTGGATAGTAAAGATGTCCGAATCAGGTTACGAGAGCCGAACTGATAAC
AACGGAGCGTCCACCAGCATGAACCCGCGCACACCGCCCAACTGCGCCCGCTGCCGCAACCATGGTCTCAAGATAGCCCTGAAGGGACACAAGCGCTATTGTAGCTTTCGAG
ACTGCAACTGCAAGAGGTGCTGCCTGACGGTCGAGCGGCAGCGGATCATGGCCAAGCAGACGGCTGACCGACGGGCGCAGGTCATGGACGAACAGCGGCTGCTGCAGGACGG
CGAAGTGCCCCCAAAACCGGAACATAACCTTCTTGTGCCGAAATTGTGCGACCTAAAAGAAATTAGCCACAGTTCGCATCCGAGAACGAACGAGTGCGATTCGTTCTCCGGC
TCGATGAACTCCAACCCGGACAGTGCGTCGGTGGCTCTGGCCCTGCACCGAAGGTCATCACCTGGCAGAGCAACGCTTCATCCACAGCAGCACGCTTCAGCCAACCATCCCA
GCGTATCACCTGAGCCCGCAAACAGCATGTTATCAGCCTCATCAGCGCGAGTCGGTCCAGAATCGGGAACAGATGACGAACTAGTGAAACGATCTCAGTGGCTTCTGGAGAA
GCTGCACTACCCGTGGGAGATGATGCCCCTAATGTATGTGATATTGAAAGGCGCCGACGGGGATGTCCATAAAGCGCACCAAAGGATCGATGAAGTGCAAATGCTGTTCAAC
GATAATAGCGAACTTCAGCCATTCTGGGGCTACCACGTGTAGCTCTACTTGTGAGACAGCGATCCTAAAGTGTGTGAAAGTGCAAACCAGTGAAGAAACCCATAGTGCAAAG
CAGGTTTTAGATAAACAAGTGATACTGCAAACGGTTTTACTACTTATTAGTTAAAGTGTTTTTCGAAAGCCGAAGTGTGTTCCACCCGCCCAGTGAAGAGAACTTCTGCCGA
CACTAAGATCACCATAAAAAAGCAACACCCAACTGGGCCAACCGCAGTGGTTCTTCCACCGACTATCATCAATCCCAACTGGGCAACCGCAGTGGTTCTGCGAACCAACTGC
CACCAATCATCAACAAATCTTTTCCAATCGTCTCGAAAATCGTTCGATTCGATTGCAACCTAAACACTGACAATTTGTCTCTCTGTCACTCGCATGCCTCGCCTGTGCATCG
TTTGATGCCATATGGCCAAGTGACTGCTAAAGCCAGCGAAAAACATGTTGGCCCAGAACGAGAAAACGAGCAGTCTCTTCTGGTCACGGTCACGGTCACGCTCTCATTCGCC
GCAAAATGACGAGGGCGTTCTCAACCTGGACATGAAATCTACCAAAGTATATGCCGCTAACGATGAAGAAGCTGCACTCGATGAATCCACCCCGCGATCTCATGATTCCGCC
GAGTTACGCTCCCGCGTACAGGTTTCCTACTACGGTAGCATAGAGCACGGTGCGAGCAGTAGCAGTTCTCATCGGAATGATGACAAGTATAGCTCGTCGGAGGACATCCAAG
TGCAGTCAGATCCGGAAGACAGCGACAAAGTGCAACTGCATCACGATGCACTTAGTAAAAGTCGGAAAGCGGCGCACAAGGATGTTGATTATTACGCAATTAAGGATAAACC
CTTTCCTCCGCTATCCAACGGATTTGAGTCAGCCGAACTGTTCAGGAACGGTCTTCACGTTGCCGGGTTGAACACGATCAATTTCACGGCAGACCTCCACCAAAAAGCTGCT
GCTGCTAACTTGATACAATACTTTCCCGTTGCGCCATGTCCCACGTTTGCACAGTTCGGGCTGTACCCACCGAATCTGGTGTATTCTCAACTGCATGGCCTAAGTTACCAGA
AGGAAGTGCTCAAGGATCACCGACCATCTTCTCCGTCCCGTTCGTCACGAACCTCGCTGGTTGACCCAGCACTGATAGATCCCAGCCATCCGCATCAGTCGTCTGCAGCCGT
AGAATCGGTCCACTAGTACGAGCTGCCACATTCGGTCGAGTGGCTCCACGTCAACGGTGCTGCCTTCTAGTTCTTGGACTGGTTGGGGAACTGCTGACTATCTATCCTGAGA
ATTATGAATCCATGCAAGCTTAACCGAAAGGCCGAAAGCTTGCATCGTGCATCTGATAAAGGTTTCGTGACGTCAGAGACGCTTGGCAGCCGCTCCCATCTGTGTAGAAAAT
GTATGTTAATCATGAGTTTTAAACTTTACACATTATGATGTATTGCTTTCTCTCACTTTCCGAGACAATTGTTTATCTATTGATTAGATAAAAAAAATGTGGTGTAAGTATT
CTGGAAAATTGTTGGCTATGTGTAAGGTAAACATTGAAATCAAGCAAAAACTGTAACTTACAGCCAGCAGAAAGCGAGGAAAGGAGTGGCTCAAATTAAATTTGAAATCATT
CACTTCAAGACTATTATGACCAAATGAATCAAAGCTGCGAAAACTACAAGTTTTCATTGTGATAACGACAACACAAGAACAAAAAGATAACGCTGGAAAATTAGGACTTAAT
AAGGATCGTTGAGGCTTACGACAATCGAAAATGAACTTCATTTATCTGATTTTTGTTATTTGTCTTGTAACAATTAATTTTCATTCTCAAATTTGAACGATTTTATTGTAAA
AACTGAAATGAAATTTATACTAAAAGATAACTGATTTGAACTATTATTTGTTAACAGTTAACAGTCTATTAAGCAGGCATGGAGAATTGGCGTTTATAACTAAAGGAAGGTT
G

>***AsudsxF*3**

ATCGCCGATGCCAATGTTCGATCAGAATTGAAGTTTTGCAAATACAAATGATAACAATCATTGATGAGTGTGAATAACTCCATTTCCCAGTGAAGCCTACACCGCAACAGGC
TGCGGTTTCGATCTTAACCGAGTGAACTGAGCTGATAAACGCGATAGCGGCCGGACATCGTTTGATTTTTCGTTGCTGTGTGCTGAAGAAAAAGCGAATATTTTAGTTTTGT
TTTTGCCCTGCTCGTTCGTACACTCGCGGGAAGCCGGTCGACACGCGGTATTTCAATAATCTCGTTTCGCTTCATTACGAATGCCAACGCCGCGCAAACGTTAGGCAAGGGC
AGTGAGTCGCGTCCGCGAAGAAAGCGCGAGGAGAAAAGCGTGAAGCATACCCCGATCGGAGTGGAGGAGCAACATTCGCAAAATTCAAAATAGCCGGCGATCGGAGGTAACA
CAGAAGTAAAAGAAGCAAATCAAAAATCCAGAAGATAATAAAACAGTGAAGCAACGGTGCTAGTTGAAAATCTTTCCAGAACAAGCGATCGGACATACCTACGGCTCGGTTA
TTCAAGTGCATGTGAGAGATTGTTTGATGATTTATTAGCGTTGCAGTATCATACTGATAGCGGCGTTGATTTGGTGCAGAAAAGGAAGATACTGCGTCTCTCCCAACGATAT
CCGATGAATTTACGGTGTTCCAAAAAAATAACAACAAATGTGTTTAAATTAATTAAAATACAGTGAGAAGGGTGACTTCCTAAAAATGAAATACTTTAAAACCTAAAAATGTT
CGAAGCGGTTCTAATCACCACGCCACACTAAAGAACGGCTGTAGAACGGATTTTCTAATTCATCGTGTTTCTGGAAAACCTCACGCAACGGAACAACACTTGTTAGCAATCG
ATTCAGACCGTCGTTTTCGGTTTCCATAAAGAGTATGTAGTGTGATTCCGGGAAATCAGATCCGACGGAAGAACATTGATCACAATCGATTGGCGCCATCGGGACGAGTATG
TTGTGGCAAAATTATTGTCCATTCGAAGGCGTCAATTCGGATGGGTCTTTGCGATCTCGTGGGCTCGATCGAGATTTTAGCTTCATGGAAGTACTTCCCATAGTAAATGTTT
TCTGAATTACGCGTCGCCTTTTGAGGGTGCGTCCTGGTACATTGTCCCCCTTCTGACCAGGATCCCGTCAGTATCGAGCAATACAGTTTTATTTTCTCTCTGTAGTAGTAGT
CATAGTAGTTACATTTGTGTGCAATATTGCGTGAGTTAGCGCGAACAGTGCAGTGATAGAGTGCACTTTCGGACCATTGCAGCTCGATAACTAGTTGAATTGAAGGAATTGT
AAGAGAGGGAAAGATCTTTTCTAGCAAGATATTGCTAAATCGGTTGATGGTTTCGCGAGAACGCTGGATAGTAAAGATGTCCGAATCAGGTTACGAGAGCCGAACTGATAAC
AACGGAGCGTCCACCAGCATGAACCCGCGCACACCGCCCAACTGCGCCCGCTGCCGCAACCATGGTCTCAAGATAGCCCTGAAGGGACACAAGCGCTATTGTAGCTTTCGAG
ACTGCAACTGCAAGAGGTGCTGCCTGACGGTCGAGCGGCAGCGGATCATGGCCAAGCAGACGGCTGACCGACGGGCGCAGGTCATGGACGAACAGCGGCTGCTGCAGGACGG
CGAAGTGCCCCCAAAACCGGAACATAACCTTCTTGTGCCGAAATTGTGCGACCTAAAAGAAATTAGCCACAGTTCGCATCCGAGAACGAACGAGTGCGATTCGTTCTCCGGC
TCGATGAACTCCAACCCGGACAGTGCGTCGGTGGCTCTGGCCCTGCACCGAAGGTCATCACCTGGCAGAGCAACGCTTCATCCACAGCAGCACGCTTCAGCCAACCATCCCA
GCGTATCACCTGAGCCCGCAAACAGCATGTTATCAGCCTCATCAGCGCGAGTCGGTCCAGAATCGGGAACAGATGACGAACTAGTGAAACGATCTCAGTGGCTTCTGGAGAA
GCTGCACTACCCGTGGGAGATGATGCCCCTAATGTATGTGATATTGAAAGGCGCCGACGGGGATGTCCATAAAGCGCACCAAAGGATCGATGAAGGTCAAGCCGTGGTCAAT
GAATACTCACGATTGCACAATCTGAACATGTTTGACGGTGTGGAATTGCGCAGTACGACGCGTCAATCCGGATGATAGACTTTTACGCGAACAGCTGTTTAACCGCGAAAG
TTGTACCCAAACAAGAACAAACATCTGTGAATTCTATAAGTCTTAAAGAAACCGGATGCAACAGAAGAAAACGACGATATCGCAAATATACAAAGCGTAACAGAAAACCCGCA
AGTTCAAACTAGTGATTACAATTAAAATATTTTAAAAAGATAGTGAAACATAGATTTTTGAAGCTAGTTTACAAAGTGCTCTACACCTAGCAAAGAGATCTAATACTAGATC
GTGGGTATTCATTTGAACCCGTCGCGTGAAACGGAAAGTGATCGAAATTAGGCCAAATGTGCGAAAAACTGTGAACACAAATGCCTTTTGGGCTGGAGAAGTTGAGGTCATT
ATAAACATCTTGAATACGGCGTCGCGGGTGGAAGTCAATCGATGCAAGTTCCCACAACTAAATTGATCAACTTCCGGCTGTGAATCGCCTTTGATTTAAAACCTTCCCGAG
GCACGGTTTTCTTTCGGTCGTTCTGATTTGTTTACTTTCTTATTTTACGAAATTATTCGCAACGAACAATGTAGTGCAAATGCTGTTCAACGATAATAGCGAACTTCAGCCA
TTCTGGGGCTACCACGTGTAGCTCTACTTGTGAGACAGCGATCCTAAAGTGTGTGAAAGTGCAAACCAGTGAAGAAACCCATAGTGCAAAGCAGGTTTTAGATAAACAAGTG
ATACTGCAAACGGTTTTACTACTTATTAGTTAAAGTGTTTTTCGAAAGCCGAAGTGTGTTCCACCCGCCCAGTGAAGAGAACTTCTGCCGACACTAAGATCACCATAAAAAA
GCAACACCCAACTGGGCCAACCGCAGTGGTTCTTCCACCGACTATCATCAATCCCAACTGGGCAACCGCAGTGGTTCTGCGAACCAACTGCCACCAATCATCAACAAATCTT
TTCCAATCGTCTCGAAAATCGTTCGATTCGATTGCAACCTAAACACTGACAATTTGTCTCTCTGTCACTCGCATGCCTCGCCTGTGCATCGTTTGATGCCATATGGCCAAGT
GACTGCTAAAGCCAGCGAAAAACATGTTGGCCCAGAACGAGAAAACGAGCAGTCTCTTCTGGTCACGGTCACGGTCACGCTCTCATTCGCCGCAAAATGACGAGGGCGTTCT
CAACCTGGACATGAAATCTACCAAAGTATATGCCGCTAACGATGAAGAAGCTGCACTCGATGAATCCACCCCGCGATCTCATGATTCCGCCGAGTTACGCTCCCGCGTACAG
GTTTCCTACTACGGTAGCATAGAGCACGGTGCGAGCAGTAGCAGTTCTCATCGGAATGATGACAAGTATAGCTCGTCGGAGGACATCCAAGTGCAGTCAGATCCGGAAGACA
GCGACAAAGTGCAACTGCATCACGATGCACTTAGTAAAAGTCGGAAAGCGGCGCACAAGGATGTTGATTATTACGCAATTAAGGATAAACCCTTTCCTCCGCTATCCAACGG
ATTTGAGTCAGCCGAACTGTTCAGGAACGGTCTTCACGTTGCCGGGTTGAACACGATCAATTTCACGGCAGACCTCCACCAAAAAGCTGCTGCTGCTAACTTGATACAATAC
TTTCCCGTTGCGCCATGTCCCACGTTTGCACAGTTCGGGCTGTACCCACCGAATCTGGTGTATTCTCAACTGCATGGCCTAAGTTACCAGAAGGAAGTGCTCAAGGATCACC
GACCATCTTCTCCGTCCCGTTCGTCACGAACCTCGCTGGTTGACCCAGCACTGATAGATCCCAGCCATCCGCATCAGTCGTCTGCAGCCGTAGAATCGGTCCACTAGTACGA
GCTGCCACATTCGGTCGAGTGGCTCCACGTCAACGGTGCTGCCTTCTAGTTCTTGGACTGGTTGGGGAACTGCTGACTATCTATCCTGAGAATTATGAATCCATGCAAGCTT
AACCGAAAGGCCGAAAGCTTGCATCGTGCATCTGATAAAGGTTTCGTGACGTCAGAGACGCTTGGCAGCCGCTCCCATCTGTGTAGAAAATGTATGTTAATCATGAGTTTTA
AACTTTACACATTATGATGTATTGCTTTCTCTCACTTTCCGAGACAATTGTTTATCTATTGATTAGATAAAAAAAATGTGGTGTAAGTATTCTGGAAAATTGTTGGCTATGT
GTAAGGTAAACATTGAAATCAAGCAAAAACTGTAACTTACAGCCAGCAGAAAGCGAGGAAAGGAGTGGCTCAAATTAAATTTGAAATCATTCACTTCAAGACTATTATGACC
AAATGAATCAAAGCTGCGAAAACTACAAGTTTTCATTGTGATAACGACAACACAAGAACAAAAAGATAACGCTGGAAAATTAGGACTTAATAAGGATCGTTGAGGCTTACGA

CAATCGAAAATGAACTTCATTTATCTGATTTTTGTTATTTGTCTTGTAACAATTAATTTTCATTCTCAAATTTGAACGATTTTATTGTAAAAACTGAAATGAAATTTATACT
AAAAGATAACTGATTTGAACTATTATTTGTTAACAGTTAACAGTCTATTAAGCAGGCATGGAGAATTGGCGTTTATAACTAAAGGAAGGTTG

>***AsudsxM***
ATCGCCGATGCCAATGTTCGATCAGAATTGAAGTTTTGCAAATACAAATGATAACAATCATTGATGAGTGTGAATAACTCCATTTCCCAGTGAAGCCTACACCGCAACAGGC
TGCGGTTTCGATCTTAACCGAGTGAACTGAGCTGATAAACGCGATAGCGGCCGGACATCGTTTGATTTTTCGTTGCTGTGTGCTGAAGAAAAAGCGAATATTTTAGTTTTGT
TTTTGCCCTGCTCGTTCGTACACTCGCGGGAAGCCGGTCGACACGCGGTATTTCAATAATCTCGTTTCGCTTCATTACGAATGCCAACGCCGCGCAAACGTTAGGCAAGGGC
AGTGAGTCGCGTCCGCGAAGAAAGCGCGAGGAGAAAAGCGTGAAGCATACCCCGATCGGAGTGGAGGAGCAACATTCGCAAAATTCAAAATAGCCGGCGATCGGAGGTAACA
CAGAAGTAAAAGAAGCAAATCAAAAATCCAGAAGATAATAAAACAGTGAAGCAACGGTGCTAGTTGAAAATCTTTCCAGAACAAGCGATCGGACATACCTACGGCTCGGTTA
TTCAAGTGCATGTGAGAGATTGTTTGATGATTTATTAGCGTTGCAGTATCATACTGATAGCGGCGTTGATTTGGTGCAGAAAAGGAAGATACTGCGTCTCTCCCAACGATAT
CCGATGAATTTACGGTGTTCCAAAAAATAACAACAAATGTGTTTAAATTAATTAAAATACAGTGAGAAGGGTGACTTCCTAAAAATGAAATACTTTAAAACCTAAAAATGTT
CGAAGCGGTTCTAATCACCACGCCACACTAAAGAACGGCTGTAGAACGGATTTTCTAATTCATCGTGTTTCTGGAAAACCTCACGCAACGGAACAACACTTGTTAGCAATCG
ATTCAGACCGTCGTTTTCGGTTTCCATAAAGAGTATGTAGTGTGATTCCGGGAAATCAGATCCGACGGAAGAACATTGATCACAATCGATTGGCGCCATCGGGACGAGTATG
TTGTGGCAAAATTATTGTCCATTCGAAGGCGTCAATTCGGATGGGTCTTTGCGATCTCGTGGGCTCGATCGAGATTTTAGCTTCATGGAAGTACTTCCCATAGTAAATGTTT
TCTGAATTACGCGTCGCCTTTTGAGGGTGCGTCCTGGTACATTGTCCCCCTTCTGACCAGGATCCCGTCAGTATCGAGCAATACAGTTTTATTTTCTCTCTGTAGTAGTAGT
CATAGTAGTTACATTTGTGTGCAATATTGCGTGAGTTAGCGCGAACAGTGCAGTGATAGAGTGCACTTTCGGACCATTGCAGCTCGATAACTAGTTGAATTGAAGGAATTGT
AAGAGAGGGAAAGATCTTTTCTAGCAAGATATTGCTAAATCGGTTGATGGTTTCGCGAGAACGCTGGATAGTAAAGATGTCCGAATCAGGTTACGAGAGCCGAACTGATAAC
AACGGAGCGTCCACCAGCATGAACCCGCGCACACCGCCCAACTGCGCCCGCTGCCGCAACCATGGTCTCAAGATAGCCCTGAAGGGACACAAGCGCTATTGTAGCTTTCGAG
ACTGCAACTGCAAGAGGTGCTGCCTGACGGTCGAGCGGCAGCGGATCATGGCCAAGCAGACGGCTGACCGACGGGCGCAGGTCATGGACGAACAGCGGCTGCTGCAGGACGG
CGAAGTGCCCCCAAAACCGGAACATAACCTTCTTGTGCCGAAATTGTGCGACCTAAAAGAAATTAGCCACAGTTCGCATCCGAGAACGAACGAGTGCGATTCGTTCTCCGGC
TCGATGAACTCCAACCCGGACAGTGCGTCGGTGGCTCTGGCCCTGCACCGAAGGTCATCACCTGGCAGAGCAACGCTTCATCCACAGCAGCACGCTTCAGCCAACCATCCCA
GCGTATCACCTGAGCCCGCAAACAGCATGTTATCAGCCTCATCAGCGCGAGTCGGTCCAGAATCGGGAACAGATGACGAACTAGTGAAACGATCTCAGTGGCTTCTGGAGAA
GCTGCACTACCCGTGGGAGATGATGCCCCTAATGTATGTGATATTGAAAGGCGCCGACGGGGATGTCCATAAAGCGCACCAAAGGATCGATGAAGCGAAAAACATGTTGGCC
CAGAACGAGAAAACGAGCAGTCTCTTCTGGTCACGGTCACGGTCACGCTCTCATTCGCCGCAAAATGACGAGGGCGTTCTCAACCTGGACATGAAATCTACCAAAGTATATG
CCGCTAACGATGAAGAAGCTGCACTCGATGAATCCACCCCGCGATCTCATGATTCCGCCGAGTTACGCTCCCGCGTACAGGTTTCCTACTACGGTAGCATAGAGCACGGTGC
GAGCAGTAGCAGTTCTCATCGGAATGATGACAAGTATAGCTCGTCGGAGGACATCCAAGTGCAGTCAGATCCGGAAGACAGCGACAAAGTGCAACTGCATCACGATGCACTT
AGTAAAAGTCGGAAAGCGGCGCACAAGGATGTTGATTATTACGCAATTAAGGATAAACCCTTTCCTCCGCTATCCAACGGATTTGAGTCAGCCGAACTGTTCAGGAACGGTC
TTCACGTTGCCGGGTTGAACACGATCAATTTCACGGCAGACCTCCACCAAAAAGCTGCTGCTGCTAACTTGATACAATACTTTCCCGTTGCGCCATGTCCCACGTTTGCACA
GTTCGGGCTGTACCCACCGAATCTGGTGTATTCTCAACTGCATGGCCTAAGTTACCAGAAGGAAGTGCTCAAGGATCACCGACCATCTTCTCCGTCCCGTTCGTCACGAACC
TCGCTGGTTGACCCAGCACTGATAGATCCCAGCCATCCGCATCAGTCGTCTGCAGCCGTAGAATCGGTCCACTAGTACGAGCTGCCACATTCGGTCGAGTGGCTCCACGTCA
ACGGTGCTGCCTTCTAGTTCTTGGACTGGTTGGGGAACTGCTGACTATCTATCCTGAGAATTATGAATCCATGCAAGCTTAACCGAAAGGCCGAAAGCTTGCATCGTGCATC
TGATAAAGGTTTCGTGACGTCAGAGACGCTTGGCAGCCGCTCCCATCTGTGTAGAAAATGTATGTTAATCATGAGTTTTAAACTTTACACATTATGATGTATTGCTTTCTCT
CACTTTCCGAGACAATTGTTTATCTATTGATTAGATAAAAAAAAATGTGGTGTAAGTATTCTGGAAAATTGTTGGCTATGTGTAAGGTAAACATTGAAATCAAGCAAAAACTG
TAACTTACAGCCAGCAGAAAGCGAGGAAAGGAGTGGCTCAAATTAAATTTGAAATCATTCACTTCAAGACTATTATGACCAAATGAATCAAAGCTGCGAAAACTACAAGTTT
TCATTGTGATAACGACAACACAAGAACAAAAAGATAACGCTGGAAAATTAGGACTTAATAAGGATCGTTGAGGCTTACGACAATCGAAAATGAACTTCATTTATCTGATTTT
TGTTATTTGTCTTGTAACAATTAATTTTCATTCTCAAATTTGAACGATTTTATTGTAAAAACTGAAATGAAATTTATACTAAAAGATAACTGATTTGAACTATTATTTGTTA
ACAGTTAACAGTCTATTAAGCAGGCATGGAGAATTGGCGTTTATAACTAAAGGAAGGTTG

>***AsufruF***
GGGGACATCACTGTAGTTCGTTTTCAGGGTCAGTCGGTTTGACGTTTGTCGTCCACTGGTGAAATGGCGAAATTTGTGATTTGACTAGCGAATGAACCGTTAACCGTGTTGT
TCCTGCTTGTGAAAATATAATATTGTGACTTCTCTTAAATGAAAGAAAGATTCGTGTCAAATTGTGTCCGTGTTTATTGAACATCTGAAGTGCAACTGTGCTCAAGTGTTGT
AATCTGTGTAAAACCTCAACCCCGAATAAAGTGTTATCCAGGCTCAAACAAACCTCGAATTGAATAGCAAACGCTTCCACAGCCGTTGAGTGCTACTGTGTACTGAGAAGTG
AATCAAAATCGAAACAAAAATAAAGTCCCGCAAGAGCCGCCGCAAGGGCATCCCATTGAAAATCGGGACACCGAAAGCCCTCAACAGCAGCAGCCACTACGAGTCTTCGCCG
CCGCAGTCGTCAATCGCTGACCCAGCCACCGAGCCCAAGAATACAGTTTCTACTAATAGTGACAGTTTGAACGGCAATGGAGACCATCAACTAGACTCATCAAGGTCCTTCA
AAATACTTCTGCAAGTACTGGACAACATGTCTTCACCACCTGCCATACCGCTCTATACCAGCCGGTATCCGACACTGAACGGTTACCCCCAGATCAACGGTGTCGATCCTGG
CGAACCAATGGATTGCCGCAAGCTTCAGTCACACCGAAGGTATGTCCAGCGGGGGTTCCATAGAAAGAAAATCAACACGAGGAAACGCATCCTCTACCATATATGCGGATTT
TAATCGTTTAACCAACCCTCTAAAATCTTGTACATTCATCATGCGAGCTTGAATCAAGGGAAACACACATATGTACTATAAAATGAAATTGAATGCTTAAATTTTACCTAAC
ATTACTCAAACAATCAAAACAAACATCAAACGAGAAACCAAATATCCTGTAAAAACGAAAAGCAGTACTAATAGGCAAATACGATAATAACTAATGATAATAATGTCAAACG
TAGCAGCAATTCAGGTTAACTGTTTTATCATAATAATATATTTAAACCAATAAAACTGTAGCAAAGTGAGAAAATAATGACATATCCTTTATCTGTTTCTCATGGAGAAAGT
ATGTGACTGACAACTGTAAAAAGTCGTAAACAAGCGAGCTGATAAATATTGCTTTTCTACTGGAGATATACTTTATACGGATTACATAAGAAAGACTTTTTCGCTTCAGTAG
AGTTTCATCGCATATAGATCCGGTAGACATGCTTTGCTTCCTGTAGTTATATGCAAGTTTATTAAAAAGTTATCCCAACTAAAATCAAAATATTATCAAGACATGTAAATGA
TCCGTAGAGTTTCCGAGTACAAAACGAACTTAGCTAAACAAAATCAAATGAAATGCAAACAAACAGTGAAACATAGCATCATTTAGCCCGATTAATTAACAATATAACCTAC
CTAGTTACGTAATTTGTACACTCAACCAAAAATATAAAATTTAAATATTTAATTCCCTTCCAACCATTAAACAAACTCCCCGCGTACAATTCGATCGAATCGAGCCAACTAA
GCCAACTAAAATCCCACCGAGTAAAAATGTGACGTTTCCCTTGCAACGAAAACCGCTCAAGCAAACTGGCAACGCTCCCGTGTGCGTCAACTATCGTTCCGGTTCATCAATC
GACACACTTCGGGATCCGTGATCCGGCTCCATCTTCAACGAACAATATCGAACCATCAATCGCTCAAATGACGCACGGGAATCAGAAGCGTTTGCCAGCTCGTCTACACCGT
CATCAATGTTGGGCGCACCGTGCGGTGCTCCAGCCAACGTGGAAGACCCTGGGCGGAAGTCGCTATGGAACAGCACTGACACAGGAACAATGGACCAGCAGTATTGCTTACG
CTGGAACAACCATCAATCCAACCTGACAACCGTGCTCAGAACCCTGCTGGAAGATGAGAAACTGTGTGATGTCACGCTGGCCTGCGATAATGGAATTGTCAAAGCACATCAA
GCGATACTGTCGGCGTGCAGTCCGTACTTTGAACAGATCTTCGTCGAGAACAAACACCCGCATCCTATCATCTACCTGCGCGATGTCGAGGTCAGTGAAATGCGCGCCCTGC
TCAACTTCATGTACCAGGGCGAGGTGAACGTTGGCCAGCACAACCTGCAGAACTTCCTCAAAACGGCGGAGAGCTTAAAAGTACGAGGTCTCACCGAGAGCAGTGCCGACCG
GTACGCATCCGAAGCCGACAAAAGCAGACCCGACCGGTCGAGGGTTGACTCGCGAGATGGACGCGACTCGGTCCCCCCAACCAGTGTCACCACCAACAACAACACCATCAAC
AGTAACAACAATAACAACAACAACAATAATAACAACAACAACAACAATACTCTCCACCACCCGCTGCAGCGGGACAAAGAACTACGTGAACGGGAAGAGCTGAGGGAACGGG
ACAGGAGGGAGGCCCATCGGGAGCTGCAGAGAGAACGGGAACTGCAGGCGCGAGAACACCAGCGCAGTGCCAGTGCCGATCTGCTCACGCCGATGACCGACGACTGCCGTTA
CTCCCCCTCGGACGACCGGGGAATGTCGGTAGTAGACAGTAAGAAAAAACGTAAAATGTCCACCTGTGATAACTCTTTGCCCTCGACACCGAGCCTGATGAACGATCGACCG
GGTGGATACGAATCGCAGGCGTCATCACACAGTAGTATCAAGCTAAGTCCAAAGCCAGACGACGAGTTCAAAGTCAGCTCACCAGCCCCGATGCATTCGCTCGCCAGTCAGA
TCAAACAAGAGTACTCTGACCTGCCCAGTCGCCATCACCCACTGCATCCAGAGCTATTGCCGAAAAATGTTTGGGGAAGTGAATCCACATCAGCATCGTTGTCGGCCGGCAA
AGCGATCGCTACTTCTTCCTCCACATCACAGCCATCGCCGTCGACGTCCACAGCCTGTAAGCCTTTGATGACGACACTAACCGGCCAAACGCTTATTACTGCCATAAAAGAG
GAGTCCCCCTCCTCCTCGACCACTCAAGACCACCACAAACACCAGCAGTTGCTTTCCTCGCTGGCTGAACTTCAGCACCTGAAGCAGCAACAGCAGCAATCCAGTGTACAAA
ATCAGCTACAGCTGTCCCAAAGTACATATCGATCATTTCGAACGCTGCGGCTGCCACTCCAAGCTTTACCAGCTGGAGCAGCCCATCGAACTTGCATCGTTACCTCCTTCAC
AGCACAGCAGCAACACAACAACCCCCTCGTGGATCTTCAAGTAGTGCCAGTGCAACCGCCGAGGAATCCCTCCTAGAGGAAATGATCCGTCCCTCGACGGTGCACACCAAAC
CTCACACACCCCGCCAAATGTCCCGCGGCGGGCGTGATCCCTCCACAACCCCGTCCTCCCGCAAAGGTGGCCGTTTTCGGCCGAACTGGCTCGAACAGTTCGACTGGCTCAA
GTACGACGAGGAGAAGAACTACATGTACTGTGCCTTCTGTCGGCGGTGGAGCAACGACATACCCGACATACGCACCTCGTTCGTTGAGGGCAACTCCAACTTTCGGCTGGAG
ATCGTCAACCATCACGACAAGTGCAAGGCCCACCGATTGTGCAAGGAACGCGAACAGCGCGAAGTGGAACTCACTCAAATGGAAACGGACCAGGACCGGGGTGGCGGCGGTG
GCGGTGTAGGTGCCGCAACTTGAGCCTCGTCCTCCTGGCCGGAAAGCAATCGCGACGATGAGGAGGAGGAGGTGAAAGTGGAATTTTGTGATAATCCAAAACCCCGTTCCCG
TGGAAGCGCCATCGCGCCAGAACTAGACCTTCTGGCGACATTTTAAATCTATAGAGACAGAGAAATGTTTATCTTTATATTAGTTGTTAAGTGTTTTGGCTTGTATTTTGAG
ACATTTTAGCAGGTGTGCTAAAACCACGAATCGCGCTGGTGAAATTTTGCAGCGGATTTTCATTTTTACCAGTAGCGCGATGCAAACATGAGTAGGTTGGTAGGTTAGTTGA
ACTTGAGAGGACCCTTACCGCCTAGCGGATGATGAAATATATTTTGTGTCAAAAGAACTGTAAGCCCTTCAGTACCATAGAGGATTACCGAATAAATGAACAGTGATGAATT

CGT

>***AsufruM***
GGGGACATCACTGTAGTTCGTTTTCAGGGTCAGTCGGTTTGACGTTTGTCGTCCACTGGTGAAATGGCGAAATTTGTGATTTGACTTGCGAATGAACCGTTAACCGTGTTGT
TCCAGCTTGTGAAAATATAATATCGTGACTTCTCTTAAATGAAAGAAAGATTCGTGTCAAATTGTGTCCGTGTTTATTGAACATCTGAAGTGCAACTGTGCTCAAGTGTTGT
AATCTGTGTAAAACCTCAATCCCAAATAAAGTGTTATCCAGGCTCAAACAAACCTCGAATTGAATAGCAAACGCTTCCACAGCCGTTGAGTGCTACTGTGTACTGAGAAGTG
AATCAAAATCGAAACAAAAATAAAGTCCCGCAAGAGCCGCCGTAAGGGCATCCCATTGAAAATCGGGACACCGAAAGCCCTCAACAGCAGCAGCCACTACGAGTCTTCGCCG
CCGCAGTCGTCAATCGCTGACCCAGCCACCGAGCCCAAGAATACAGTTTCTACTAATAGTGACAGTTTGAACGGCAATGGAGACCATCAACTAGACTCATCAAGGTCCTTCA
AAATACTTCTGCAAGTACTGGACAGCATGTCTTCGCCACCTGCCATACCGCTCTATACCAGCCGGTATCCGACACTGAACGGTTACCCCCAGATCAACGGTGTCGATCCTGG
CGAACCAATGGATTGCCGCAAGCTTCAGTCACACCGAAGGAACAGCACTGACACAGGAACAATGGACCAGCAGTATTGCTTACGCTGGAACAACCATCAATCCAACCTGACA
ACCGTGCTCAGAACCCTGCTGGAAGATGAGAAACTGTGTGATGTCACGCTGGCCTGCGATAATGGAATTGTCAAAGCACATCAAGCGATACTGTCGGCGTGCAGTCCGTACT
TTGAACAGATCTTCGTCGAGAACAAACACCCGCATCCTATCATCTACCTGCGCGATGTCGAGGTCAGTGAAATGCGCGCCCTGCTCAACTTCATGTACCAGGGCGAGGTGAA
CGTTGGCCAGCACAACCTGCAGAACTTCCTCAAAACGGCGGAGAGCTTAAAAGTACGAGGTCTCACCGAGAGCAGTGCCGACCGGTACGCATCCGAAGCCGACAAAAGCAGA
CCCGACCGGTCGAGGGTTGACTCGCGAGATGGACGCGACTCGGTCCCCCCAACCAGTGTCACCACCAACAACAACACCATCAACAGTAACAACAATAACAACAACAACAACA
ATAATAACAACAACAACAACAATACTCTCCACCACCCGCTGCAGCGGGACAAAGAACTACGTGAACGGGAAGAGCTGAGGGAACGGGACAGGAGGGAGGCCCATCGGGAGCT
GCAGAGAGAACGGGAACTGCAGGCGCGAGAACACCAGCGCAGTGCCAGTGCCGATCTGCTCACGCCGATGACCGACGACTGCCGTTACTCCCCCTCGGACGACCGGGAAATG
TCGGTAGTAGACAGTAAGAAAAAACGTAAAATGTCCACCTGTGATAACTCTTTGCCCTCGACACCGAGCCTGATGAACGATCGACCGGGTGGATACGAATCGCAGGCGTCAT
CACACAGTAGTATCAAGCTAAGTCCAAAGCCAGACGACGAGTTCAAAGTCAGCTCACCAGCCCCGATGCATTCGCTCGCCAGTCAGATCAAACAAGAGTACTCTGACCTGCC
CAGTCGCCATCACCCACTGCATCCAGAGCTATTGCCGAAAAATGTTTGGGGAAGTGAATCCACATCAGCATCGTTGTCGGCCGGCAAAGCGATCGCTACTTCTTCCTCCACA
TCACAGCCATCGCCGTCGACGTCCACAGCCTGTAAGCCTTTGATGACGACACTAACCGGCCAAACGCTTATTACTGCCATAAAAGAGGAGTCCCCCTCCTCCTCGACCACTC
AAGACCACCACAAACACCAGCAGTTGCTTTCCTCGCTGGCTGAACTTCAGCACCTGAAGCAGCAACAGCAGCAATCCAGTGTACAAAATCAGCTACAGCTGTCCCAAAGTAC
AATATCGATCATTTCGAACGCTGCGGCTGCCACTCCAAGCTTTACCAGCTGGAGCAGCCCATCGAACCTGCATCGTTACCTTGCTACACAGCACAGCAGCACCAACACAACC
CACTCGTGGATTCTTTCAAAGTAGTGCCAGTGCAACCGCCCGAGGAATTCCCTCCTAGAGGAAATGATCCCGTCCCTCGACGGTGCACACCAAACCTCACACACCCCGCCAA
ACGTCCCGCGGCGGGCGTGATCCCTCCACAACCCCGTCCTCCCGCAAAGGTGGCCGTTTTCGGCCGAACTGGCTCGAACAGTTCGACTGGCTCAAGTACGACGAGGAGAAGA
ACTACATGTACTGTGCCTTCTGTCGGCGGTGGAGCAACGACATACCCGACATACGCACCTCGTTCGTTGAGGGCAACTCTAACTTTCGGCTGGAGATCGTCAACCATCACGA
CAAGTGCAAGGCCCACCGATTGTGCAAGGAACGCGAACAGCGCGAAGTGGAACTCACTCAAATGGAAACGGACCAGGACCGGGGTGGCGGTGGTGGCGGCGTAGGTGCCGCA
ACTTGAGCCTCGTCCCCCTGGCCGGAAAGCAATCGCGGCGATGAGGAGGAGGAGGTGAAAGTGGAATTTTGTGATAATCCAAAACCCCGTTCCCGTGGAAGCGCCATCGCGC
CAGAACTAGACCTTCTGGGGACATTTTAAATCTATAGAGACAGAGAAATGTTTATCTTTATATTAGTTGTTAAGTGTTTTGGCTTGTATTTTGAGACATTTTAGCAGGTGTG
CTAAAACCCCGAATCGCGCTGGTGAAATTTTGCAGCGGATTTTCATTTTTACCAGTAGCGCGATGCAAACATGAGTAGGTTGGTAGGTTAGTTGAACTTGAGAGGACCCTTA
CCGCCTAGCGGATGATGAAATATATTTTGTGTCAAAAGAACTGTAAGCCCTTCTGTACCATAGAGGATTACCGAATAAATGAAGTGATGAATTCG

Supplementary Text3. DBHS Protein Sequences.

>***AsuMf1* [*Armigeres subalbatus*]**
MDVVKPVFSDKHQQGDGGSNGRSGNLNNLRSSKQQQQRVVNKQNRNQNKNLGGNQNQQKRDGTRGNHRNIDDDKDQVFKRRRSGPGEEFFINKKLRMLQGPLTDISPIETEE
NKFFGRNRLFIGNLTNDATEDELIELFRPFGDISEIFMNKDKNYAFVRVDYFSNAVKAKRELEGTLHKNRMLRLRFAPSATIIRVRNLTPWVSDELLFKSFEVFGSVERAFV
HVDERGKSTGKGIVEFKNKPAALVALRYCTDKCYFLTASLRPVIVEPYTYKDDSVPEKSMNKKHPDFYKARQKGPRFAEYSSFEHEYGQRWKQLYELYRQKAEALKREMIME
EEKLEAQIEFARYEHETEQLREELRKRERNRDRQKADLKTKIRIASEDKRRKDMQMKPDVQTGHCTDYDLQRIRKKSNVFMPVCN

>***AsuHrp65* [*Armigeres subalbatus*]**
MEVAKPEVNGNPLPQRQQNNPNAQPQQQGGGGGVQGGNQNNQGGNQNNQGGPNQQQGGGGNKQNRNQQNKNRGGNQNRFGNQNRNQGGGGGGGPGGNRQNNPDGGNMGGDGG
NHQQQQQQRGGGGGGGPGFNRGNRRTMGGDNDQGFDRRRSGPGEQYFINEKLRMLQGPLMDIPPIESEEAKFSGRNRLYIGNLTNDVTEDELTELFRPYGDISEIFMNKDKN
YAFVRVDYFSNAEKAKRELEGTMRKNRMLRVRFAPNATAIRVRNLTPWVSNELLFKSFEVFGPVERASVHVDERGKSTGEGIVEFKNKPGAMVALRYCTEKCYFLTASLRPV
IVEPYTYQDDTDGLPEKSMNKKIPDFQKARQHGPRFADHGSFEHEYGQRWKQMHELYKQKAESLKREMIMEEEKLEGSWRKRSWKLRWNLPVTSMRPSSFENNCVCVSRIVT
VRRPNGK

>**AaeHrp65 [AAEL017116-PB] [*Aedes aegypti*]**
MEVAKPEVNGSPLPQRQQNNPNAQQQGQQQGGPGGLQGGNPNQGQGGGPNQQQQQGGKQQQQQNRNQQNKNRGGNQNRFGNRNQGGGGGGPGGNRQNNPDGGNMGGGDGGNP
QHQQQQQRGGGGGGPGGNRGNRRNMGGDDNQGGFDRRRSGPGEQYFINEKLRMLQGPLMDIPPIETEEVKFSGRNRLYIGNLTNDITEEELVELFRPYGDISEVFMNKEKNY
AFVRVDYFSNAEKAKRELEGTMRKNRMLRVRFAPNATAIRVRNLTPWVSNELLFKAFEVFGPVERASVHVDERGKSTGEGIVEFKNKPGAMVALRYCSEKCFFLTASLRPVI
VEPYTYQDDTDGLPEKSMNKKIPDFQKARQQGPRFADHGSFEHEYGQRWKQMHELYKQKAEALKREMIMEEEKLEAQMEFARYEHETEQLREQLRMREQDRDRQKAEWEMKE
RMASEAKQRNDMQMKHDVEEMQNRIKRTDEELHRRQQENNVFMQQNQQMGALMDNNDDNRRAFEMMNQGGGGGGGGGNAGPGGNNFGGMEVRDYDHGQNRPSRFNDDGPQQN
QQGGGGGGNQQRNSFGGGQNRNWMNNDRRNNDDFQSKRRRF

>**AalbHrp65 [AALB003148-PA] [*Anopheles albimanus*]**
MMNPVVKQEINGSPLPQRIPQQQHVGGDGGGGGGVPGGNQPGGGGNTSGNDQHGPGGPGGKGNMNRNNNRNNSKNRLSMPNRNRGPGGMGMGGGGGGGGNMGPGNNRSGNNG
GNNMQQSPQQGGGDDSGPNPHMNRGMHRGNRGGNDMDNSFVDRRRGGGGDGYFINEKLRLLQGPLLDIPPIEAQEAKFSGRNRLYVGNLTNDATEEELMEMFKPFGEISEVF
MNKEKNYAFVRVDYFSNAEKAKRELDGTSRKNRVLRVRFAPNATAIRVKNLTAYVTNELLYKAFEVFGPLERAVVQVDERGKPTGEGIVEFQKKPGAAAAIRYCTEKCYFLT
SSLRPVIVEPYTYQDDNDGLPEKSLNKKQNEFIKARQLGPRFAENGSFEYEYGQRWKHMHDMYRQKAEALKREMIMEEEQLEAKMEFARYEHETEQLREQLRQREMDRDRQK
AELEMKERQVHVARQRNDMQVKTEMDEMTNRIKRSDEELDRRQKENNMFMKNQQMNFMDQQQQMNDMGGMGGGGGGGGGGGGNDNRRNFDMNQGPGNGNFMNS

>**AalHrp65 [AALF004221-PA] [*Aedes albopictus*]**
MEVAKPEVNGSPLPQRQQNNPNAQQQQQQQGGLQGGNPNQGGGGGGGGPNQQQGQGGGNKQMNRNQQNKNRGGNQNRFGNRNQQGGPGGNRQNNPDGGNMGGEGGGNQQQQQ
RGGGGGGGPGGNRGNRRNMGDNDQGGFDRRRSGPGEQYFINEKLRMLQGPIMDIPPIEAEEAKFTGRNRLYIGNLTNDVTEEELVELFRPYGDISEIFMNKEKNYAFVRVDY
FSNAEKAKRELEGTMRKNRMLRVRFAPNATAIRVRNLTPWVSNELLYKAFEVFGPVERASVHVDERGKSTGEGIVEFKSKPGAMVALRYCTEKCFFLTASLRPVIVEPYTYQ
DDTDGLPEKSMNKKIPDFQKARQHGPRFADHGSFEHEYGQRWKQMHELYKQKAEALKREMIMEEEKLEAQMEFARYEHETEQLREQLRMREQDRDRQKAEWEMKERMASEAK
QRNDMQMKHDVEEMQNRIKRTDEELHRRQQENNVFMQQNQQMGGGPMDNNDDNRRPPPFDLMNQGGGAGGNAGPGGNNFGGMEVRDYEHGQNRPSRFNDDGPQQNQQGGGGG
GNQQRNSFGGGNNRNWMNNDRRNNDDFQSKRRRF

>**AgaHrp65 [AGAP003794-PB] [*Anopheles gambiae*]**
MDVAVKQEINGSPLPQRPQQQPHGGGGGGPGPMQGNNQAGPDQHGPGGPGGPGGPGGPGGPGGPGGPGNKNMNRNNNRNNSKNRQSMPNRNRGPGGNAGGGGGNPNNNGGPGN
NRPGNNGGNPQQMGGEDFAYFINEKLRQLAGPLFEIPPIEAQDVKFSGRNRLYVGNLTNDVTEEELVDMFKVYGEISEVFMNKEKNYAFVRVDYLSNAEAAKRGLDGTTRKN
RVLRVRFAPNATAIRVKNLTPYVTNELLYKAFEVFGPLERAVVQVDERGKPTGEGIVEFKNKPGAMAAIRYCTEKCYFLTASLRPVIVEPYTYQDDNDGLPEKSLNKKQNDF

LKARQQGPRFAEHGSFEFEYGQRWKHMHELFKQKAESLKREMIMEEEKLEAQMEYARYEQETELLREQLRQREMDRDRQKAEWEMKERQVQEARQRNDIQIKTELEEMTTRI
KRSDEELHRRQKENNMFMQQQMNDMGGMGSGGDGGGGSGIDNRRNFDMMNQVVGGGGGGGGGGGGGGNGNFGNMFEKEYLSELLRLLGTGSRDNAQGPNNINMKGKTANKKKT
FRGGPRPNK

>**AstHrp65 [XP035890878.1] [*Anopheles stephensi*]**
MDVVVKQELNGSPLPQRPQQQQQQHGGGGGGGGGTPGPMHGNNQSGGPDGPGGPGNKNMNRNNRNNSKNRLSMPMRNRGPGGVGGVGGGGAGGNGNNGGPGNNRPGNNGGNP
QQMGGEDFSPNQHQNRGGMNRGGHNRSGNDMDNNFGDRRRGGGEAYFINEKLRMLAGPLLDIPPIEAHDTKFSGRNRLYVGNLTTDVTEEELIEMFKPFGEINEVFMNKEKN
YAFVRVDYLSNAEAAKRNLDGTTRKNRVLRVRFAPNATAIRVKNLTPYVTNELLYKAFEVFGPLERAVVQVDERGKPTGEGIVEFKNKPGALAAIRCCTEKCFFLTSSLRPV
IVEPYTHQDDNDGLPEKSLNKKQGEFMKARQQGPRFAEQGSFEFEYGQRWKHMHELFKQKAESLKREMIMEEEKLEAQMEYARYEQETEQLREQLRQREMDRDRQKAEWEMK
ERQAQEARQRNDIQMKTEIEEMTNRIKRSDEELHRRQKENTMFMQNRQMMEQQQMNDMGMGSGGDAGGGIDNRRNFDMMNQGGGNGGNFGNMEPTVRDYDHGQNRPTRFDDG
PQPQQQQQQQQQQQQQMHQQQRGNNFGGNRNNWMNNTPDRRLNNNDNDFQNKRRRF

>**CquHrp65 [CQUJHB015709.P24371] [*Culex quinquefasciatus*]**
MEVEQPVDNGSPMPQRQQNQNQGGGGPGGRGGGGGGGKQFQNNRNRNNRGGGGGGMNRGGGGGRNSFGGGNRGGGGGGGGFRQNQDNQDGNGDGDNQQQNQGGFQNRGGGRG
GRNRFSGGGGQGGDGDQGFDRRRSGPGEMYFIGEKLRMLQGQLLDIPPIDETDAKFSGRNRLYVGNLTPDVTEEELVELFLPYGEITEVFMNMEKNYAFVRVDFFSNAEKAK
RELDGTSRKNRILKIRFAPNATALRVSNLGPFVTNELLYRAFEVFGPVESAKVQVDERGKSTGEGIVEYKNKPSASAALKHCSEKCFFLDSSLRPCFVEPYTYQDNNSDGLS
EKLINKKIPEFLKSRQQGPRFADQGSFEHEYGQRWKHMHEMYKQKVEALKRDMVMEEEKLEAQMEFARHEHEIEQLREQLRMREQDKDRKKAEWEMKERFVNESRERLQMQM
SDQRMGFQGNNSFNNRNNSFDMMNQGGGGGNRNNSFGGEVRDYGHGGQKRQSRFDNHEMNQMNQQQDQQQGQQQQQQQPQQGLPDQPEQQGPGQEQGPGGPAAEGGMGDAAN
IVGGNGGGVGAGPVGIEQLLSNAFGGGGGNNNRNNNPWNNGGGNNGGNRGDDFQNKRRRF

>**CquHrp65-1 [CQUJHB016553.P25562] [*Culex quinquefasciatus*]**
MEAAPAAAKPEVNGNPLPQRQQKNQNQQKQGGAAGGAGGGDGGNQGQQNQQQQQQGGGNKNQNQNNRNRNRGGGANGPNRGGGGGRNNFGNRNNQQGGGGQDAGNGEGGNNQ
QQQQNNQGFQNRNQGGPKNRGGPRNQGGDGEGGGGFDRRRANNEQYFIGEKLRLLQGPLLDLPAIESTEEVKFSGRNRLYIGNLTADVTEEELVELFMPYGEITEIFMNKEK
LYAFVRVDFFSNAEKAKRELDGTSRKNRILRVRFAPNATAIKVKNLTQDVSNELLYKAFEVFGPLERAVVQVDERGKSTGEGIVEYKNKPSAMAAQRYCSERCYFLNSSLRP
CIVEPYMYQDNNADGLPEKSLNKKIPEFMKLRQQGPHFADQGSFEHEYGQRWKQMHDLFKQKSDALKREMEMEEEKLEAQMEYARYEHETEQLREQLRMREQDSDRQKAEWE
MKERMAVENKQRNEIQMKHKVEEMQNLIKRTDEELHRRQLENNVFMQNQQMNLLDGMDDNRQGFGNMRGGNNFDAGMEIRDYDHGQNNRQSRFDDGGMQQQQGGGGVNQRLG
NSFNAGGNNRNWMSNNRGGGGGGGGGANNDDFQSKRRRF

>**DmelnonA-l [FBpp0305874] [*Drosophila melanogaster*]**
MEGAVKKNSLNSSPLPQRQQRGNSTNKNLGKPTPPKLNAASDGNPAEKKARLGGNTQNGGGVAGGGGTGGGGGGGGATGGVEFSRNRNRGGNQNENRQGFQVANNSHQKQIN
ESPKPAAGNVPAKNNELSAGGGGQNQPNHSNKGQGNQGDQGEQGNQGPNFRGRGGGPNQPNQNANQEQSNGYPGNQGDNKGGQGQRGAGGGKHQRGNRSRRSGGSGIMNSSM
GGGGQRGEDFFIAQRLLDISGPTHELPPIELPTDNKFVGRNRLYVGNLTSDTTDDDLREMFKPYGEIGDIFSNPEKNFTFLRLDYYQNAEKAKRALDGSLRKGRVLRVRFAP
NAIVRVTNLNQFVSNELLHQSFEIFGPIERAVICVDDRGKHTGEGIVEFAKKSSASACLRLCNEKCFFLTASLRPCLVEPMEVNNDNDGLPDKTLNKKSLEFRHERSVGPRF
ACLNSFEHEYGSRWKQLHDLFKSKQDSLKRELKMEEDKLEAQMEYARYEQETELLRQELKKRELDNERMKLEWEMREKQAEEIRKREEEYMHRYQNQLLRHEEDMRARQQEN
DLLMQAKKLNMILDQQEGFGGSNSGFEHFDSPFEVFGNNSNNSTMAGPGGPDNSDGNQHGHIDSWGHRRF

>**DmelnonA [FBpp0074012] [*Drosophila melanogaster*]**
MESAGKQDNNATQQLPQRQQRGNQQANKNLGKHNAQKQNDSADGGPAEKKQRFGGPNAQNQNQNQNQNGGVTGGGGAVGGPNQNKNFGNNKGGFVGNRNRNNNRAGNQNRTF
PGNNNPNQKPNNETSKADGPNALAKNNEPATAAAGQNQANQNANKGQNQRQGQNQNQNQVHGQGNQGGPGNQGGAGNQGGQGNQGGAGNQGNGQGFRGRNAGNNQGGGFSGG
PQNQQRDNRNRSGPRPGGGAGGAMNSTNMGGGGGGGGGGGPRGGEDFFITQRLRSISGPTFELEPVEVPTETKFSGRNRLYVGNLTNDITDDELREMFKPYGEISEIFSNLD
KNFTFLKVDYHPNAEKAKRALDGSMRKGRQLRVRFAPNATILRVSNLTPFVSNELLYKSFEIFGPIERASITVDDRGKHMGEGIVEFAKKSSASACLRMCNEKCFFLTASLR
PCLVDPMEVNDDTDGLPEKAFNKKMPDFNQERSIGPRFADPNSFEHEYGSRWKQLHNLFKTKQDALKRELKMEEDKLEAQMEYARYEQETELLRQELRKREVDNERKKLEWE
MREKQAEEMRKREEETMRRHQTEMQSHMNRQEEDMLRRQQETLFMKAQQLNSLLDQQEGFGGGGGGNNSTFDNFAGNSNSPFEVFRGNNNNNSTMIGNNAAPNTQEIKRLDG
PINEITSNFSPPDLDSDIILVAFVLEEFSSHVFYTKIVQKCESFKKRTQLNARSNIIKKCISNLNWLIVL

>**HasPSPC1 [ENSP00000481916.1] [*Homo sapiens*]**
MMLRGNLKQVRIEKNPARLRALESAVGESEPAAAAAMALALAGEPAPPAPAPPEDHPDEEMGFTIDIKSFLKPGEKTYTQRCRLFVGNLPTDITEEDFKRLFERYGEPSEVF
INRDRGFGFIRLESRTLAEIAKAELDGTILKSRPLRIRFATHGAALTVKNLSPVVSNELLEQAFSQFGPVEKAVVVVDDRGRATGKGFVEFAAKPPARKALERCGDGAFLLT
TTPRPVIVEPMEQFDDEDGLPEKLMQKTQQYHKEREQPPRFAQPGTFEFEYASRWKALDEMEKQQREQVDRNIREAKEKLEAEMEAARHEHQLMLMRQDLMRRQEELRRLEE
LRNQELQKRKQIQLRHEEEHRRREEEMIRHREQEELRRQQEGFKPNYMENREQEMRMGDMGPRGAINMGDAFSPAPAGNQGPPPMMGMNMNNRATIPGPPMGPGPAMGPEGA
ANMGTPMMPDNGAVHNDRFPQGPPSQMGSPMGSRTGSETPQAPMSGVGPVSGGPGGFGRGSQGGNFEGPNKRRRY

>**HsaNONO [ENSP00000362963.4] [*Homo sapiens*]**
MQSNKTFNLEKQNHTPRKHHQHHHQQQHHQQQQQQPPPPPIPANGQQASSQNEGLTIDLKNFRKPGEKTFTQRSRLFVGNLPPDITEEEMRKLFEKYGKAGEVFIHKDKGFG
FIRLETRTLAEIAKVELDNMPLRGKQLRVRFACHSASLTVRNLPQYVSNELLEEAFSVFGQVERAVVIVDDRGRPSGKGIVEFSGKPAARKALDRCSEGSFLLTTFPRPVTV
EPMDQLDDEEGLPEKLVIKNQQFHKEREQPPRFAQPGSFEYEYAMRWKALIEMEKQQQDQVDRNIKEAREKLEMEMEAARHEHQVMLMRQDLMRRQEELRRMEELHNQEVQK
RKQLELRQEEERRRREEEMRRQQEEMMRRQQEGFKGTFPDAREQEIRMGQMAMGGAMGINNRGAMPPAPVPAGTPAPPGPATMMPDGTLGLAGVQWCNLGSLQPPPPRFKRF
SCLSLLSSWDYRPHQQLNALVRLLQWKELGQLVELLLHSTVQLLELNLPQTNVADTNKLQCLVSQNP

>**HasSFPQ [ENSP00000349748.5] [*Homo sapiens*]**
MSRDRFRSRGGGGGGFHRRGGGGGRGGLHDFRSPPPGMGLNQNRGPMGPGPGQSGPKPPIPPPPPHQQQQQPPPQQPPPQQPPPHQPPPHPQPHQQQQPPPPPQDSSKPVVA
QGPGPAPGVGSAPPASSSAPPATPPTSGAPPGSGPGPTPTPPPAVTSAPPGAPPPTPPSSGVPTTPPQAGGPPPPPAAVPGPGPGPKQGPGPGGPKGGKMPGGPKPGGGPGL
STPGGHPKPPHRGGGEPRGGRQHHPPYHQQHHQGPPPGGPGGRSEEKISDSEGFKANLSLLRRPGEKTYTQRCRLFVGNLPADITEDEFKRLFAKYGEPGEVFINKGKGFGF
IKLESRALAEIAKAELDDTPMRGRQLRVRFATHAAALSVRNLSPYVSNELLEEAFSQFGPIERAVVIVDDRGRSTGKGIVEFASKPAARKAFERCSEGVFLLTTTPRPVIVE
PLEQLDDEDGLPEKLAQKNPMYQKERETPPRFAQHGTFEYEYSQRWKSLDEMEKQQREQVEKNMKDAKDKLESEMEDAYHEHQANLLRQDLMRRQEELRRMEELHNQEMQKR
KEMQLRQEEERRRREEEMMIRQREMEEQMRRQREESYSRMGYMDPRERDMRMGGGGAMNMGDPYGSGGQKFPPLGGGGGIGYEANPGVPPATMSGSMMGSDMRTERFGQGGA
GPVGGQGPRGMGPGTPAGYGRGREEYEGPNKKPRF

>**MmuNONO [ENSMUSP00000033673.7] [*Mus musculus*]**
MQSNKAFNLEKQNHTPRKHHQHHHQQHHQQQQQQQQQQPPPPIPANGQQASSQNEGLTIDLKNFRKPGEKTFTQRSRLFVGNLPPDITEEEMRKLFEKYGKAGEVFIHKDKG
FGFIRLETRTLAEIAKVELDNMPLRGKQLRVRFACHSASLTVRNLPQYVSNELLEEAFSVFGQVERAVVIVDDRGRPSGKGIVEFSGKPAARKALDRCSEGSFLLTTFPRPV
TVEPMDQLDDEEGLPEKLVIKNQQFHKEREQPPRFAQPGSFEYEYAMRWKALIEMEKQQQDQVDRNIKEAREKLEMEMEAARHEHQVMLMRQDLMRRQEELRRMEELHNQEV
QKRKQLELRQEEERRRREEEMRRQQEEMMRRQQEGFKGTFPDAREQEIRMGQMAMGGAMGINNRGAMPPAPVPPGTPAPPGPATMMPDGTLGLTPPTTERFGQAATMEGIGA
IGGTPPAFNRPAPGAEFAPNKRRRY

>**MmuPSPC1 [ENSMUSP00000022507.6] [*Mus musculus*]**
MMLRGNLKQVRIEKNPARLRALESAAGESEPVAAAAMALTLAGEQAPPPAPSEEHPDEELGFTIDIKSFLKPGEKTYTQRCRLFVGNLPTDITEEDFKRLFERYGEPSEVFI
NRDRGFGFIRLESRTLAEIAKAELDGTILKSRPLRIRFATHGAALTVKNLSPVVSNELLEQAFSQFGPVEKAVVVVDDRGRATGKGFVEFAAKPPARKALERCGDGAFLLTT
TPRPVIVEPMEQFDDEDGLPEKLMQKTQQYHKEREQPPRFAQPGTFEFEYASRWKALDEMEKQQREQVDRNIREAKEKLEAEMEAARHEHQLMLMRQDLMRRQEELRRLEEL
RNQELQKRKQIQLRHEEEHRRREEEMIRHREQEELRRQQEGGFKPNYMENREQEMRMGDMGPRGAINMGDAFSPAPAGTQGPPPMMGMNMNNRGTIPGPPMGPGPAMGPEGA
ANMGTPMIPDNGAVHNDRFPQGPPSQMGSPMGNRTGSETPQAPMSGVGPVSGGPGGFGRGSQGGNFEGPNKRRRY

>**MmuSFPQ [ENSMUSP00000030623.8] [*Mus musculus*]**
MSRDRFRSRGGGGGGFHRRGGGGGRGGLHDFRSPPPGMGLNQNRGPMGPGPGGPKPPLPPPPPHQQQQQPPPQQPPPQQPPPHQQPPPHQPPHQQPPPPPQESKPVVPQGPG
SAPGVSSAPPPAVSAPPANPPTTGAPPGPGPTPTPPPAVPSTAPGPPPPSTPSSGVSTTPPQTGGPPPPPAGGAGPGPKPGPGPGGPKGGKMPGGPKPGGGPGMGAPGGHPK
PPHRGGGEPRGGRQHHAPYHQQHHQGPPPGGPGPRTEEKISDSEGFKANLSLLRRPGEKTYTQRCRLFVGNLPADITEDEFKRLFAKYGEPGEVFINKGKGFGFIKLESRAL
AEIAKAELDDTPMRGRQLRVRFATHAAALSVRNLSPYVSNELLEEAFSQFGPIERAVVIVDDRGRSTGKGIVEFASKPAARKAFERCSEGVFLLTTTPRPVIVEPLEQLDDE
DGLPEKLAQKNPMYQKERETPPRFAQHGTFEYEYSQRWKSLDEMEKQQREQVEKNMKDAKDKLESEMEDAYHEHQANLLRQDLMRRQEELRRMEELHSQEMQKRKEMQLRQE
EERRRREEEMMIRQREMEEQMRRQREESYSRMGYMDPRERDMRMGGGGTMNMGDPYGSGGQKFPPLGGGGGIGYEANPGVPPATMSGSMMGSDMRTERFGQGGAGPVGGQGP
RGMGPGTPAGYGRGREEYEGPNKKPRF
