# Supplementary material for: A DBHS family member regulates male determination in the filariasis vector Armigeres subalbatus
Source: Nat Commun. 2023 Apr 21;14:2292. doi: 10.1038/s41467-023-37983-y (PMC10121658; doi:10.1038/s41467-023-37983-y)
Supplement: Supplementary file 3 — Description of Additional Supplementary Files [file 41467_2023_37983_MOESM3_ESM.pdf]

Description of Additional Supplementary Files

File Name: Supplementary Data 1
Description: Sequences of oligonucleotide primers and probes.

File Name: Supplementary Data 2
Description: Phenotypes observed in partially feminized *AsuMf*$^{-}$ mosaic males.
