# Supplementary material for: A DBHS family member regulates male determination in the filariasis vector Armigeres subalbatus
Source: Nat Commun. 2023 Apr 21;14:2292. doi: 10.1038/s41467-023-37983-y (PMC10121658; doi:10.1038/s41467-023-37983-y)
Supplement: Supplementary file 6 — Reporting Summary [file 41467_2023_37983_MOESM6_ESM.pdf]

nature portfolio

Corresponding author(s): Zhijian Jake Tu
Anthony A. James
Xiao-Guang Chen

Last updated by author(s): Mar 20, 2023

# Reporting Summary

Nature Portfolio wishes to improve the reproducibility of the work that we publish. This form provides structure for consistency and transparency in reporting. For further information on Nature Portfolio policies, see our Editorial Policies and the Editorial Policy Checklist.

## Statistics

For all statistical analyses, confirm that the following items are present in the figure legend, table legend, main text, or Methods section.

| n/a | Confirmed | |
|---|---|---|
| ☐ | ☒ | The exact sample size (*n*) for each experimental group/condition, given as a discrete number and unit of measurement |
| ☐ | ☒ | A statement on whether measurements were taken from distinct samples or whether the same sample was measured repeatedly |
| ☐ | ☒ | The statistical test(s) used AND whether they are one- or two-sided<br>*Only common tests should be described solely by name; describe more complex techniques in the Methods section.* |
| ☒ | ☐ | A description of all covariates tested |
| ☒ | ☐ | A description of any assumptions or corrections, such as tests of normality and adjustment for multiple comparisons |
| ☒ | ☐ | A full description of the statistical parameters including central tendency (e.g. means) or other basic estimates (e.g. regression coefficient) AND variation (e.g. standard deviation) or associated estimates of uncertainty (e.g. confidence intervals) |
| ☐ | ☒ | For null hypothesis testing, the test statistic (e.g. *F*, *t*, *r*) with confidence intervals, effect sizes, degrees of freedom and *P* value noted<br>*Give P values as exact values whenever suitable.* |
| ☒ | ☐ | For Bayesian analysis, information on the choice of priors and Markov chain Monte Carlo settings |
| ☒ | ☐ | For hierarchical and complex designs, identification of the appropriate level for tests and full reporting of outcomes |
| ☒ | ☐ | Estimates of effect sizes (e.g. Cohen's *d*, Pearson's *r*), indicating how they were calculated |

*Our web collection on statistics for biologists contains articles on many of the points above.*

## Software and code

Policy information about availability of computer code

| | |
|---|---|
| Data collection | SMZ1000 stereomicroscope (Nikon, Tokyo, Japan), AB17500 svstem (Thermo Fisher, Waltham, MA, USA) |
| Data analysis | Significant differences among the data groups were analysed in GraphPad Prism 8.<br>Hisat2 v2.2.1<br>Trinity v2.11.0<br>BlastX v2.11.0+<br>tBLASTn v2.11.0<br>featurecount v2.0.3<br>EdgeR v3.2.4<br>pheatmap v1.0.12<br>hmmsearch v3.3.1<br>MUSCLE v3.8.1551<br>TrimAl v.4.rev15<br>IQ-TREE 2 (v2.0.3)<br>Mega X (v10.1.8)<br>OrthoFinder v2.5.4 |

For manuscripts utilizing custom algorithms or software that are central to the research but not yet described in published literature, software must be made available to editors and reviewers. We strongly encourage code deposition in a community repository (e.g. GitHub). See the Nature Portfolio guidelines for submitting code & software for further information.

# Data

Policy information about availability of data

All manuscripts must include a data availability statement. This statement should provide the following information, where applicable:

- Accession codes, unique identifiers, or web links for publicly available datasets
- A description of any restrictions on data availability
- For clinical datasets or third party data, please ensure that the statement adheres to our policy

The full sequences of four AsuMf isoforms, three Asudsx isoforms, and two Asufru isoforms are deposited in GenBank under accession number ON427922 [https://www.ncbi.nlm.nih.gov/nuccore/ON427922] for AsuMf1, ON427923 [https://www.ncbi.nlm.nih.gov/nuccore/ON427923] for AsuMf2, ON427924 [https://www.ncbi.nlm.nih.gov/nuccore/ON427924] for AsuMf3, ON427925 [https://www.ncbi.nlm.nih.gov/nuccore/ON427925] for AsuMf4, ON427927 [https://www.ncbi.nlm.nih.gov/nuccore/ON427927] for AsudsxF1, ON427928 [https://www.ncbi.nlm.nih.gov/nuccore/ON427928] for AsudsxF2, ON427929 [https://www.ncbi.nlm.nih.gov/nuccore/ON427929] for AsudsxF3, ON427930 [https://www.ncbi.nlm.nih.gov/nuccore/ON427930] for AsudsxM, ON427931 [https://www.ncbi.nlm.nih.gov/nuccore/ON427931] for AsufruF, and ON427932 [https://www.ncbi.nlm.nih.gov/nuccore/ON427932] for AsufruM. All resulting High-throughput sequencing data have been deposited in the NCBI SRA database (PRJNA834573) [https://www.ncbi.nlm.nih.gov/bioproject/PRJNA834573/]. The NOPS domain (PF08075 [https://www.ebi.ac.uk/interpro/entry/pfam/PF08075/] was obtained from Pfam database v35.0. All other data are available in the main text or supplementary materials. Source data are provided in this paper.

# Human research participants

Policy information about studies involving human research participants and Sex and Gender in Research.

| | |
|---|---|
| Reporting on sex and gender | NA |
| Population characteristics | NA |
| Recruitment | NA |
| Ethics oversight | NA |

Note that full information on the approval of the study protocol must also be provided in the manuscript.

# Field-specific reporting

Please select the one below that is the best fit for your research. If you are not sure, read the appropriate sections before making your selection.

☒ Life sciences ☐ Behavioural & social sciences ☐ Ecological, evolutionary & environmental sciences

For a reference copy of the document with all sections, see nature.com/documents/nr-reporting-summary-flat.pdf

# Life sciences study design

All studies must disclose on these points even when the disclosure is negative.

| | |
|---|---|
| Sample size | Sample size was chosen consistent with the previous literature reporting similar assays (e.g., Science, 2015, 348(6240): 1268-1270; Insect biochemistry and molecular biology, 2020, 118: 103311). Simple sizes for PCR, real-time PCR, gene knockout, and RNA-seq were designed for at least three samples in each group for analysis. Samples sizes information is provided In main text (Figure 1 legend, Figure 3 legend), and Supplemental Material (Materials and Methods section, and legends of Fig. S2, Fig. S4, and Fig. S7. |
| Data exclusions | No data were excluded. |
| Replication | All experiments were replicated three or more times. |
| Randomization | Mosquitoes of same eclosion time were separated into different groups randomly in each experiment. |
| Blinding | No blinding occurred during these studies. Experimental design did not require blinding because assessed variables are not confounded by the evaluator. |

# Reporting for specific materials, systems and methods

We require information from authors about some types of materials, experimental systems and methods used in many studies. Here, indicate whether each material, system or method listed is relevant to your study. If you are not sure if a list item applies to your research, read the appropriate section before selecting a response.

## Materials & experimental systems

| n/a | Involved in the study |
|---|---|
| ☒ | ☐ Antibodies |
| ☒ | ☐ Eukaryotic cell lines |
| ☒ | ☐ Palaeontology and archaeology |
| ☐ | ☒ Animals and other organisms |
| ☒ | ☐ Clinical data |
| ☒ | ☐ Dual use research of concern |

## Methods

| n/a | Involved in the study |
|---|---|
| ☒ | ☐ ChIP-seq |
| ☒ | ☐ Flow cytometry |
| ☒ | ☐ MRI-based neuroimaging |

# Animals and other research organisms

Policy information about studies involving animals; ARRIVE guidelines recommended for reporting animal research, and Sex and Gender in Research

| | |
|---|---|
| Laboratory animals | The Armigeres subalbatus GZ strain (Guangzhou Guangdong Province, China) was established in the laboratory in 2018 and reared in 30-cm3 nylon cages in the insectary at 28 ± 1℃ with 70-80% humidity and a 12:12h (light: dark) light cycles. Larvae were fed with finely-ground fish food mixed 1:1 with yeast powder, and adults were fed after emergence with a 10% glucose solution and mated freely. Female adults were blood-fed with defibrinated sheep blood 3 days post-emergence for egg production. To validate the male-specificity of AsuMf, genomic DNA was extracted from pools of five male and female adults respectively, with four replicates for each sex. To examine the transcription of AsuMf, doublesex (dsx), and fruitless (fru), total RNA was extracted from a range of developmental samples, including approximately 200 embryos collected at each stage (i.e., 0-2h, 2-4h, 4-8h, 8-12h, 12-24h, 24-48h post-oviposition), as well as 30 1st and 2nd star larvae, 20 3rd and 4th instar larvae, 15 sex-mixed pupae, 15 male adults, and 15 female adults. RNA-seq samples include approximately 200 embryos collected at each stage (i.e., 0-1h, 2-4h, 4-8h, 8-12h post-oviposition), along with 15 sex-mixed pupae, 15 male adults, and 15 female adults. For 5' and 3' RACE, total RNA was extracted from ~200 embryos, 4–8 and 12–16 hours post deposition, and 15 male adults with TRIzol® Reagent. FISH was performed on Ar. subalbatus (GZ strain) mitotic chromosomes derived from fourth instar larvae. In addition to the RNAseq experiments described earlier, four mosaically-feminized AsuMf – individuals that were two days post-eclosion were also selected for RNA-seq. |
| Wild animals | No wild animals. |
| Reporting on sex | For mosquito feeding blood experiment, mosquito should be female. Other experiment includes male and female. |
| Field-collected samples | No field-collected samples. |
| Ethics oversight | The study did not require an ethical approval. |

Note that full information on the approval of the study protocol must also be provided in the manuscript.
